# Light-induced quantum friction of carbon nanotubes in water

**DOI:** 10.1038/s41586-026-10632-2

**Published:** 2026-06-10

**Authors:** Tanuja Kistwal, Krishan Kanhaiya, Adrian Buchmann, Chen Ma, Jana Nikolić, Julia Ackermann, Phillip Galonska, Sanjana S. Nalige, Vahideh Sardari, Aishwarya Sudarsan, Martina Havenith, Marialore Sulpizi, Sebastian Kruss

**Affiliations:** 1 Department of Chemistry and Biochemistry, Ruhr-University Bochum, Bochum, Germany; 2 Department of Chemistry, Amity Institute of Applied Science, Amity University, Noida, India; 3 Department of Physics and Astronomy, Ruhr-University Bochum, Bochum, Germany; 4 Department of Chemical Engineering, Indian Institute of Technology, Hyderabad, India; 5 Fraunhofer Institute of Microelectronic Circuits and Systems, Duisburg, Germany

**Keywords:** Nanoscience and technology, Carbon nanotubes and fullerenes, Optical spectroscopy, Excited states

## Abstract

Friction slows down moving objects at both macroscopic and microscopic scales^1^. At the electronic level, quantum friction describes direct transfer of momentum between a liquid and the electrons of a solid^2^. Owing to its microscopic nature, this phenomenon remains experimentally challenging to capture^3^. Here we show that near-infrared fluorescent single-walled carbon nanotubes (SWCNTs) exhibit light-induced quantum friction in water. It is measured by observing an excitation-power-dependent linear decrease of around 50% in the diffusion constants of functionalized SWCNTs in aqueous solution. This effect disappears when excitons are localized, as in the case of SWCNTs with quantum defects. We further show that the chemical manipulation of exciton concentration by molecules that increase or decrease SWCNT fluorescence also modulates the diffusion constant by up to a factor of 2. Optical pump terahertz (THz) probe spectroscopy shows an instantaneous response (around 30 cm^−1^) that we assign to direct exciton–water coupling in the range of water Debye modes. It is followed by an increasing (>100 ps) response in the range of intermolecular translational modes of the hydrogen bond network of water (>100 cm^−1^), resembling heating. Classical molecular dynamics simulations further support a mechanism in which the fluctuating dipole moments of excitons create frictional forces. These findings establish light-induced quantum friction between excitons in SWCNTs and water and show that electronic excitations can be used to control nanoscale motion and fluid properties.

## Abstract (web summary)

Near-infrared fluorescent carbon nanotubes exhibit light-induced quantum friction in water, in which exciton interactions slow nanoscale motion and enable optical control of diffusion and fluid dynamics.

## Main

Friction is a well-known phenomenon, with the first quantitative description dating back to Leonardo da Vinci (Amonton’s law). To move an object over a surface, a force proportional to the normal force (weight) is necessary. At the nanoscale, friction becomes more complex because of surface topography^1^. A fundamentally different type of friction has been theoretically proposed and coined quantum friction^2^.

It describes non-adiabatic coupling between collective modes of solvent dipoles and electronic modes of materials such as graphene, graphite and carbon nanotubes (CNTs)^2,4,5^. The friction is expected to increase when the surface response function of the substrate overlaps with the low-frequency spectrum of the solvent, including librational, intermolecular stretch and Debye modes^4^. Experimental studies have provided evidence supporting coupling beyond the Born–Oppenheimer approximation between classical water dynamics and the quantum dynamics of confined delocalized electrons. This is demonstrated by anomalies in hydrodynamic friction at water–carbon interfaces^6,7^ and by the rapid cooling of hot electrons in graphene in water^3^. Optical pump terahertz (THz) probe experiments showed faster cooling in water compared with other solvents, which was attributed to near-field radiative heat transfer (NFRHT) between graphene surface plasmon modes and water charge fluctuations in the frequency range of the librational mode of water in the THz region^3^. THz spectroscopy can probe solute–solvent interactions^8^, including those driven by charge fluctuations^5^. Notably, these charge-fluctuation-driven interactions extend beyond the primary hydration shell^9^, influencing solvation dynamics in a broader sense. The anomalously high water friction on graphite, as well as the unique slippage behaviour observed in CNTs, has been attributed to THz plasmon modes^2,4^.

Semiconducting single-walled carbon nanotubes (SWCNTs) are one-dimensional nanomaterials that fluoresce in the near-infrared (NIR) tissue transparency window^10,11^. Their fluorescence is best described by electron–hole pairs called excitons^12^, which diffuse along the axis of the SWCNTs for around 100 nm (refs. ^13,14^). Excitons are affected by changes in the surrounding dielectric environment caused by bundling^15^, surfactants^16^ or DNA wrapping^17^. SWCNTs themselves are hydrophobic^18^, but adsorption of surfactants, peptides, proteins^19,20^ or π-stacking of nucleic acids^21^ makes them water-soluble. Their surface chemistry can be further tuned by covalent functionalization, which introduces a low number of σ-bonds into the *sp*^2^ hybridized carbon lattice (*sp*^3^ quantum defects). They act as local traps for excitons and create new photophysics^22–25^. These optoelectronic properties of SWCNTs are highly sensitive to their chemical environment, which makes them ideal building blocks for (bio)sensors^18^ that can image chemical signalling by cells^26,27^, for cancer or pathogen diagnostics^28,29^, or to image plant stress^30,31^. The fluorescence changes (that is, exciton decay or energy shift) of these biosensors have been attributed to conformational changes and changes in local solvation^5^.

Here, we study whether excitons affect the diffusion of fluorescent SWCNTs in water. We use physical manipulation by changing (light) excitation, and chemical control by adding analytes or changing surface chemistry to identify how excitons affect friction and, consequently, diffusion. Based on THz spectroscopy to explore exciton–water interactions and molecular dynamics simulations, we propose a mechanism for the observed phenomena.

**Fig. 1:**
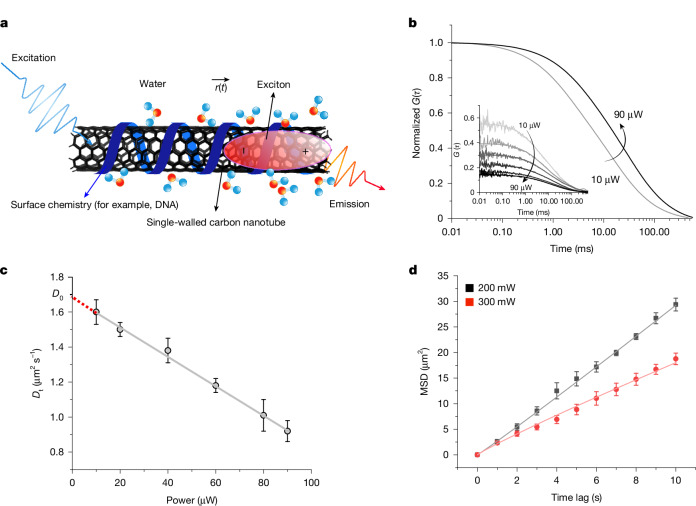
Light-induced diffusion changes of CNTs in water. **a**, Schematic of experimental design. SWCNTs modified with a biopolymer such as DNA are water-soluble. Upon optical excitation, excitons are created that decay by either emitting NIR photons or dissipating energy. The SWCNTs diffuse due to Brownian motion. **b**, Inset, FCS of (GT)_10_-SWCNTs shows an excitation power-dependent change of the fluorescence (>900 nm) autocorrelation function (*λ*_exc_ = 480 nm). The normalized and fitted (equation (4) and Supplementary Table 1) autocorrelation functions indicate slower diffusion with increasing power. **c**, Corresponding diffusion constants of (GT)_10_-SWCNTs at different excitation power (*n* = 3, mean ± s.d.). *D*_0_ represents the translational diffusion constant (*D*_t_)  extrapolated (red dashed line) to zero excitation power from the linear fit (grey) of the data (*R*^2^ = 0.996). **d**, Ensemble time-averaged MSD plots of single DOC-SWCNTs tracked in a wide-field microscope (>5 particles, >100 frames each) show slower diffusion at higher light intensity (mean ± s.e., *n* = 4).

**Extended Data Fig. 1:**
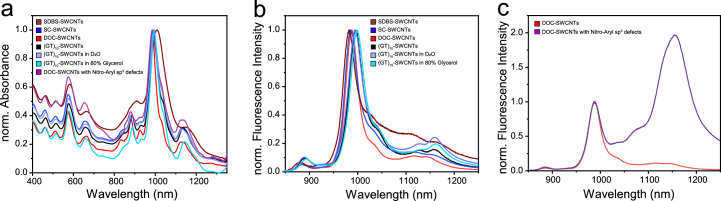
Spectra of SWCNT samples in different surfactants. a) Normalized absorbance and b) normalized fluorescence spectra of SDBS functionalized SWCNTs (SDBS-SWCNTs) (brown), SC functionalized SWCNTs (SC-SWCNTs) (dark blue), DOC functionalized SWCNTs (DOC-SWCNTs) (red), DNA functionalized SWCNTs (GT)_10_-SWCNTs in PBS buffer (black),(GT)_10_-SWCNTs in PBS (neon blue), (GT)_10_-SWCNTs in 80% glycerol (cyan). c) Emission spectra of DOC-SWCNTs with (wine) and without sp^3^ (Nitro-Aryl) quantum defects (red).

**Extended Data Fig. 2:**
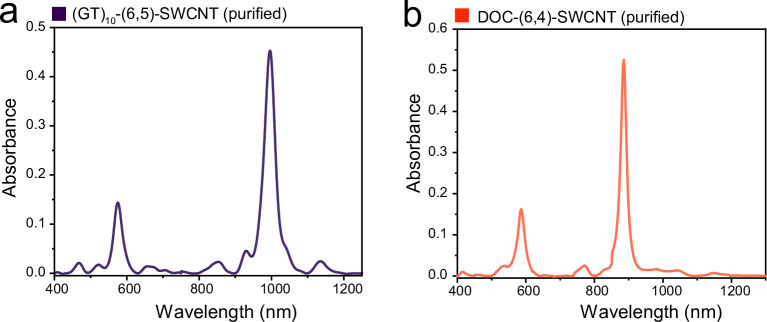
Absorbance spectra of chirality enriched SWCNT samples. a) (GT)_10_-(6,5)-SWCNTs. b) DOC-(6,4)-SWCNTs. Samples were purified with ATPE.

We conducted single-molecule fluorescence measurements to explore the diffusion behaviour of SWCNTs in water under light excitation (Fig. 1a). For this purpose, the hydrophobic SWCNTs (mainly semiconducting (6,5)-chirality) were functionalized with single-stranded DNA ((GT)_10_) or surfactants (deoxycholate (DOC), sodium cholate (SC) and sodium dodecyl benzene sulfonate (SDBS)) (Extended Data Figs. 1 and 2 and Supplementary Figs. 1–5). Moreover, we prepared SWCNTs with nitro-aryl *sp*^3^ quantum defects, which trap and localize excitons^23,25^.

In NIR FCS (fluorescence correlation spectroscopy) measurements, we observed a decrease in the initial fluorescence autocorrelation amplitude *G*(0) for increasing laser power (ranging from 10 μW to 90 μW) for (GT)_10_-SWCNTs (Fig. 1b, inset), which can be expected for an NIR fluorophore of the length of a SWCNT (600 nm) with low quantum yield^18^. The normalized and fitted (equation (4)) correlation amplitude at low and high laser power (Fig. 1b) showed that diffusion slowed down with higher power. By fitting the diffusion constants at different laser powers with a linear equation, we extrapolated the translational diffusion constant (*D*_t_) value (1.7 μm^2^ s^−1^) for (GT)_10_-SWCNTs at zero power (Fig. 1c). This value is comparable to previously reported diffusion constants of 0.4–2.3 µm^2^ s^−1^, depending on the conditions and the lengths of the SWCNTs^32,33^. We performed a variety of control experiments to rule out effects by changes in confocal volume, heating, sample purity and so on (Supplementary Figs. 6–9 and Supplementary Tables 1 and 2). All these results indicate that light excitation increases friction between SWCNTs and water and consequently slows down SWCNT diffusion.

**Extended Data Fig. 3:**
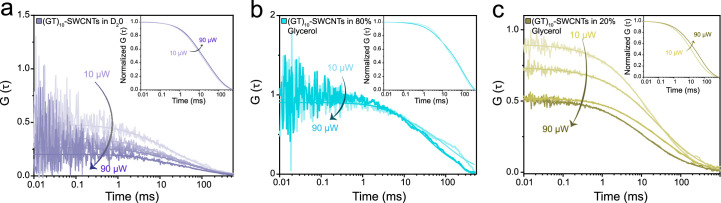
Solvent effects on diffusion. Excitation power-dependent change (10 μW – 90 μW) of fluorescence autocorrelation functions of SWCNTs in different solvents. a) (GT)_10_-SWCNTs in D_2_O-based PBS (Table ST2), b) (GT)_10_-SWCNTs in 80% glycerol in water (Table ST3), c) (GT)_10_-SWCNTs in 20% glycerol in water (Table ST4).

Next, we changed the solvent from water (H_2_O) to heavy water (D_2_O) (only solvents that provide colloidal stability are possible). (GT)_10_-SWCNTs in a D_2_O-based phosphate-buffered saline (PBS) buffer also showed a power-dependent diffusion behaviour (Extended Data Fig. 3a and Supplementary Table 3), however, less pronounced. The same decrease of the effect was found for glycerol–water mixtures (Extended Data Fig. 3b,c and Supplementary Tables 4 and 5). These findings are in line with more pronounced cooling dynamics by quantum friction between graphene and water compared with other solvents such as methanol and D_2_O (ref. ^3^). Hence, these findings suggest that efficient coupling to water THz modes is relevant for the decrease of diffusion.

**Extended Data Fig. 4:**
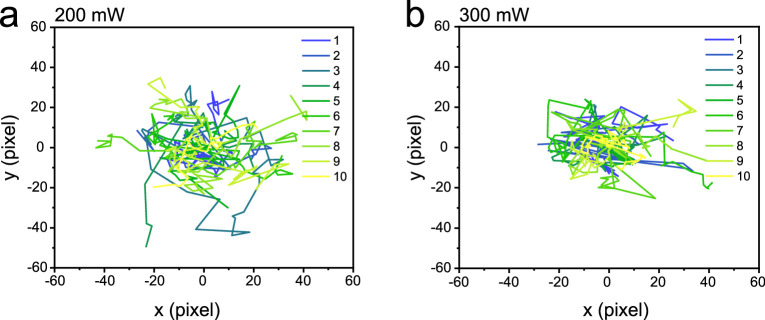
Particle trajectories. Single-particle trajectories of DOC-SWCNTs diffusing in aqueous solution under (a) 200 mW and (b) 300 mW laser power. The labels 1 to 10 indicate individual SWCNT trajectories (t = 10 s each).

Another question is whether light-induced friction at the nanoscale can have effects on larger microscopic or macroscopic length scales. Imaging of polystyrene (PS) beads (5 µm diameter) in an SWCNT solution showed slower movements (Supplementary Fig. 10). However, the relationship between viscosity and friction is most likely complex, and, therefore, single SWCNT tracking (using wide-field microscopy) is a more direct way to quantify movements on the ≫1 µm scale (Extended Data Fig. 4 and Supplementary Fig. 11). Wide-field particle tracking (Fig. 1d, and Supplementary Table T6) of SWCNTs showed that light decreases diffusion, leading to smaller regions that are explored by the random walk of the SWCNT. This kind of tracking can, in principle, be extended to longer SWCNTs with lengths above the resolution limit to study the role of rotational and anisotropic diffusion or SWCNT undulations (Supplementary Fig. 12).

**Fig. 2:**
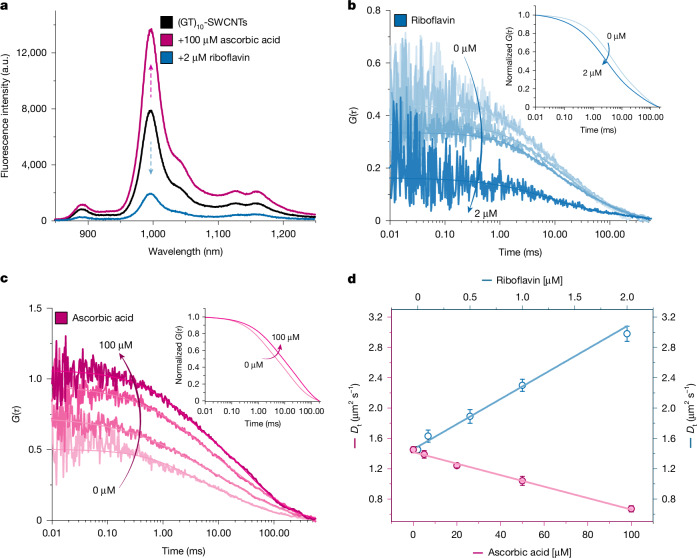
Chemical manipulation of light-induced friction. **a**, Fluorescence spectrum of (GT)_10_-SWCNTs without an analyte (black), with 100 μM ascorbic acid (deep pink), and 2 μM riboflavin (sapphire blue) in PBS buffer. **b**,**c**, Riboflavin (0–2 μM) (**b**) and ascorbic acid (0–100 μM) (**c**) change the autocorrelation curves of (GT)_10_-SWCNTs in a concentration-dependent manner. The insets show the normalized and fitted autocorrelation function. **d**, Diffusion constants of (GT)_10_-SWCNTs in response to different concentrations of ascorbic acid and riboflavin (*n* = 3, mean ± s.d.). Fits are linear equations (*R*^2^ = 0.98 for ascorbic acid and *R*^2^ = 0.99 for riboflavin).

Increasing the light excitation is one way to vary the exciton concentration. Another way is to change how fast or efficiently excitons decay. This mechanism, which allows tuning the properties of SWCNTs, is the basis for their use as molecular sensors and biosensors^18^. Thus, we added analytes that change the fluorescence quantum yield to study the chemical manipulation of diffusion. The fluorescence of (GT)_10_-SWCNTs increases in the presence of ascorbic acid and decreases in the presence of riboflavin (Fig. 2a). Previously, we found that fluorescence changes are anti-correlated to changes in low-frequency THz absorption, which suggests a coupling of charge fluctuations in SWCNTs to charge density fluctuations in the hydration layer^5^. In agreement with this hypothesis, riboflavin shifted the normalized autocorrelation curves (Fig. 2b) and reduced the diffusion time (Supplementary Table 7). By contrast, ascorbic acid slowed down the diffusion of SWCNTs and increased the diffusion time (Fig. 2c and Supplementary Table 8). For different analyte concentrations, diffusion increased linearly for analytes that decreased the quantum yield and vice versa (Fig. 2d). In both cases, the diffusion constants changed by a factor of around 2 (Fig. 2d and Supplementary Tables 7 and 8) without changes in the absorption spectra after addition of ascorbic or riboflavin (Supplementary Fig. 13).

**Fig. 3:**
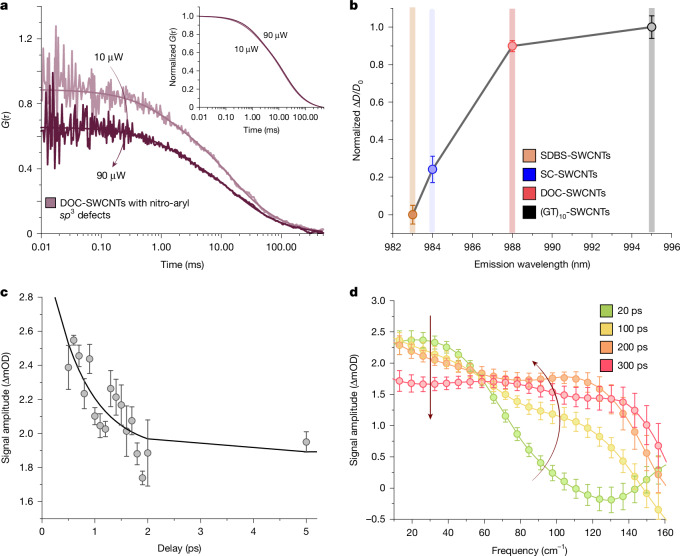
The organic corona shields light-induced friction and excitons change THz absorption by water. **a**, Excitation power-dependent fluorescence autocorrelation functions of DOC-SWCNTs with nitro-aryl *sp*^3^ quantum defects (wine) that serve as exciton traps show no power-dependent diffusion. The inset shows the normalized and fitted autocorrelation function. **b**, Normalized diffusion constant changes increase with emission wavelength for different functionalized SWCNTs (mean ± s.d., *n* = 3). Lines serve as visual guides. **c**, OPTP measurements of an aqueous DOC-SWCNTs solution. Plot of the maximum amplitudes of ΔmOD at a given initial time delay below 60 cm^−^^1^. The fitted exponential is also shown as a solid line, yielding a decay time of 0.71 ± 0.24 ps (mean ± s.e., *n* = 22). **d**, Vertical slices at representative longer pump–probe time delays (mean ± s.e., *n* = 22; the arrows serve as a visual guide to the eye).

**Extended Data Fig. 5:**
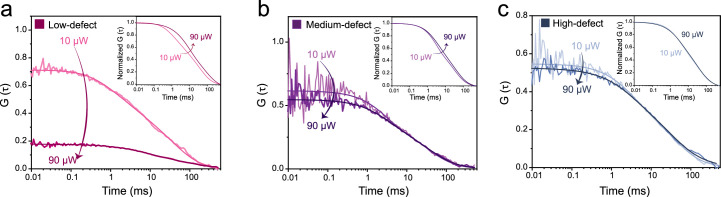
FCS experiments of (GT)_10_-SWCNTs with less deep (guanine) quantum defects. Comparison between a) low (reaction with 5 μM Rose Bengal), b) medium (10 μM Rose Bengal) and c) high (20 μM Rose Bengal) guanine defect density (samples prepared as described before^68^). The insets show the normalized and fitted autocorrelation functions.

In the next step, we modified the direct chemical environment and organic corona around a SWCNT to see how this affects light-induced changes of SWCNT diffusion. In the case of DOC-functionalized SWCNTs (DOC-SWCNTs), we observed a similar trend in power dependency as for (GT)_10_-SWCNTs (Supplementary Fig. 14a), and the diffusion time increased (Supplementary Table 9). By contrast, DOC-SWCNTs with nitro-aryl *sp*^3^ defects did not change their diffusion on exposure to light, and we observed no dependency on excitation power (Fig. 3a and Supplementary Table 10). This is in line with the expectation that trapped and localized (non-moving) excitons are not seen by the solvent as fluctuating dipoles. We also performed experiments with another type of quantum defect (guanine quantum defects). They are less deep exciton traps and are expected to slow down exciton diffusion and mobility^34^. The power dependence of diffusion decreased with increasing quantum defect density (Extended Data Fig. 5), which suggests that exciton mobility is more important than local polarizability and charge fluctuations.

Surfactants such as SC and SDBS change the chemical environment seen by the SWCNT. They exhibited the same power-dependent trend as when DNA was used for functionalization (Supplementary Fig. 14b,c). However, different coronas should affect the hydrodynamic radius as well, and diffusion was faster for DNA- and SDBS-coated SWCNTs compared with DOC and SC (Supplementary Fig. 14d).

**Extended Data Fig. 6:**
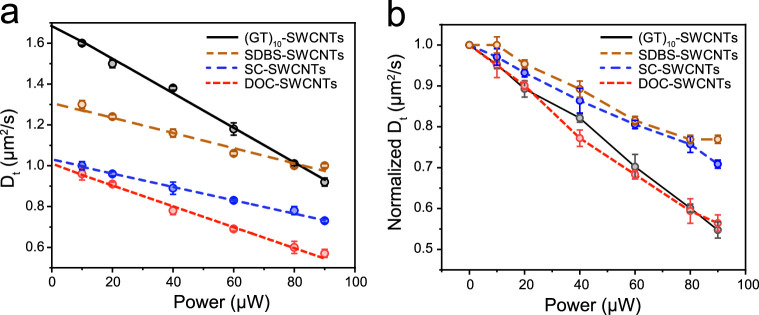
Impact of surface chemistry/corona shielding on diffusion. a) Diffusion constants as a function of excitation power. The diffusion constants at zero power were fitted by linear equations (Table ST12). b) Normalized diffusion constants as a function of power for (GT)_10_-SWCNTs (black), SDBS-SWCNTs (brown), SC-SWCNTs (blue), and DOC-SWCNTs (red) (n = 3, mean ± SD).

To disentangle the effects of hydrodynamic radius and light excitation on diffusion, we measured the power-dependent diffusion constants for different types of functionalized SWCNTs (Extended Data Fig. 6a). The extrapolated diffusion constants at zero power represent the impact of the hydrodynamic radius and SWCNTs length on diffusion. By contrast, the normalized excitation power-dependent changes in diffusion reflect light-induced changes (Extended Data Fig. 6b). This normalized change of the diffusion constant correlated with the SWCNTs emission wavelength (Fig. 3b). A redshift in fluorescence emission is expected if the SWCNT is exposed to more water^35^. Therefore, a less dense corona (DOC, DNA) allows the exciton to ‘see’ more water, which suggests that the coupling of the exciton in the SWCNT with water is responsible for the change in diffusion.

**Extended Data Fig. 7:**
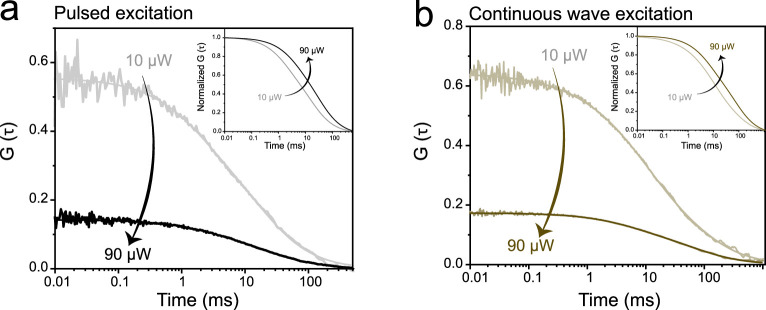
Comparison of pulsed and continuous laser excitation for (GT)_10_-SWCNTs at low (10 µW) and high (90 µW) excitation power. a) Autocorrelation trace for pulsed excitation (similar to Fig. 1b). b) Autocorrelation trace for continuous wave (CW) excitation. Inset: Normalized curves demonstrating the impact on diffusion.

Another question that arises is how SWCNT chirality and thus curvature affect light-induced changes in diffusion. We extrapolated the diffusion constant and determined the value at zero excitation power (*D*_0_) to be 1.00 μm^2^ s^−1^ for DOC-(6,4)-SWCNTs and 0.97 μm^2^ s^−1^ for DOC-SWCNTs (with mainly (6,5)-SWCNTs). Thus, for these (similar) SWCNT chiralities, the effect on diffusion was similar (Supplementary Fig. 15a,b), but it could be different for larger diameter SWCNTs. Slowing down of diffusion in FCS measurements was also observed for both pulsed and continuous wave (CW) excitation (Extended Data Fig. 7). All these results show that the changes in diffusion are directly related to the presence of excitons. Friction and diffusion are linked to each other inversely by the Stokes–Einstein equation, and thus, exciton-induced friction could be an explanation.

To probe the coupling between excitons and water, we conducted optical pump terahertz probe (OPTP)^36^ measurements of DOC-SWCNTs. We observed an increase in THz absorption in the entire spectral range at all timescales (Fig. 3c,d and Supplementary Fig. 16a,b). The observed OPTP spectrum can be dissected into three distinct responses. An initial, instantaneous signal below 60 cm^−^^1^ (Fig. 3c) increased linearly in amplitude with increasing optical pump power (Supplementary Fig. 16d) and decayed within <1 ps (Supplementary Fig. 17). We interpret it as exciton relaxation to the bandgap energy level and energy dissipation to water. The decay times were faster than reported for dry gelatine/SWCNT films^37^, which is similar to the faster cooling of hot electrons in graphene by quantum friction in water^3^. This process should not involve reorientation of the nuclei in the water network, which would take a longer response time. The OPTP spectrum (on timescales >1 ps) can be further dissected into two additional responses (Fig. 3d). A decreasing signal below 50 cm^−^^1^ and a subsequent increasing signal >100 cm^−^^1^, which becomes dominant for pump–probe delays above 200 ps (Supplementary Fig. 18).

For a quantitative description, we carried out singular value decomposition of the time-dependent spectra (Supplementary Figs. 19 and 20). The first component had a maximum around 30 cm^−1^ (1 THz) and persisted during the lifetime of the excitons (10 ps up to around 100 ps; refs. ^38–40^). Such a feature had not been observed in THz OPTP spectra of other dyes after excitation^41^. The second component (100–120 cm^−1^) continuously increased with increasing pump–probe delay time. It resembles OPTP changes expected for heating of water (Supplementary Fig. 18). This spectral response is expected for energy transfer from thermalized phonons to water by translational modes or propagating acoustic phonons^42^.

Thus, we interpret the 30 cm^−^^1^ feature as direct coupling between the exciton and water, which appears instantaneously and decreases with decreasing exciton concentration. We propose that the 30 cm^−^^1^ feature reports NFRHT by a coupling that could also serve as pathway to exchange momentum by quantum friction. It is followed by a heat signature (>100 cm^−1^). This mechanistic picture aligns best with the experimental results, which showed that the change in the diffusion constant is proportional to the exciton concentration (Figs. 1c and 2d).

**Fig. 4:**
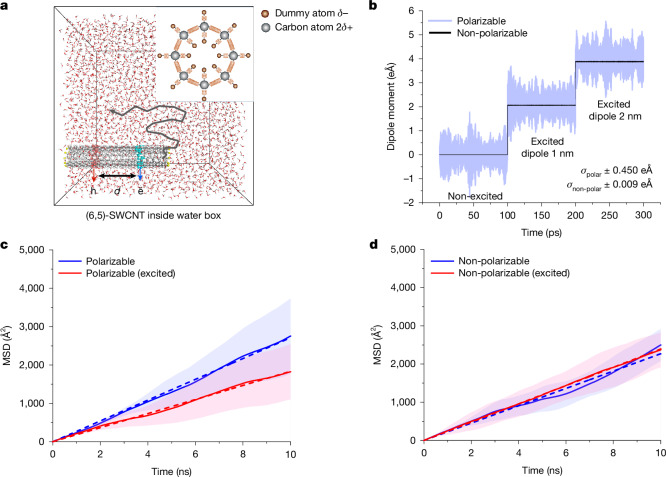
Exciton-related charge fluctuations change diffusion. **a**, Molecular dynamics simulation of excited SWCNTs. A (6,5)-SWCNT (length 4.1 nm) is allowed to move freely (black trace) in a three-dimensional periodic water box. The ends of the SWCNT are capped with hydrogens (yellow). Excitons are modelled by placing charges in the blue (e, electron) and red (h, hole) annular regions separated by the distance. Inset, schematic of the SWCNT cross-section. For each carbon atom (grey, charge 2*δ*+), two dummy or virtual atoms (brown, charge *δ*− each) are placed at a distance of 0.6 Å to mimic the electrostatics of a π-electron cloud. The virtual atoms provide polarizability in response to solvent and ion dynamics. In the current model, a charge of Δ*δ* = 0.22 *e* was chosen for the exciton. **b**, Dipole moment fluctuations of polarizable (blue) and non-polarizable (black) models of excitons in SWCNTs (without excitation in the first 100 ps, with excitation and 1 nm or 2 nm dipole in the next 100 ps periods). *σ* denotes the standard deviation of the fluctuations (200,000 data points for each condition). **c**,**d**, Molecular dynamics simulation of SWCNT diffusion for polarizable (**c**) and non-polarizable (**d**) models with and without excitation. The mean square displacement (MSD) is plotted against time (solid lines). Dashed lines indicate linear fits. The shaded region indicates the standard deviation (*n* = 6 for excited and *n* = 3 for non-excited). Here, the mean of 1 nm dipole and 2 nm dipole (exciton) simulations is shown.

**Extended Data Fig. 8:**
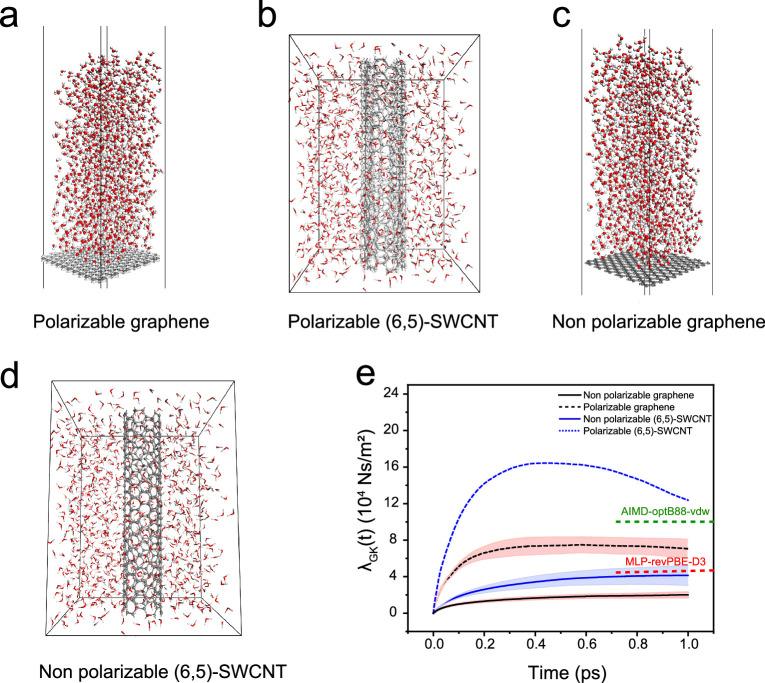
Interfacial friction of graphene and (6,5)-SWCNTs with water. a) Polarizable graphene-water interface modelled with IFF-CVFF. b) Polarizable SWCNT-water interface modelled with IFF-CVFF. In the polarizable models, a carbon atom (grey balls) is attached to two additional negatively virtual atoms (white balls attached to carbon atoms in panels (a and b) to mimic a ${\rm{\pi }}$ electron cloud. c) Non-polarizable graphene-water interface modeled with CVFF. d) Non-polarizable SWCNT-water interface modeled with CVFF. In the non-polarizable models, a carbon atom (grey balls) is attached only to adjacent carbon atoms, and thus it has no coulombic interaction with the solvent. In panels (a and c), the graphene water interface system is made non-periodic by leaving a vacuum space inside the 3D periodic simulation box. In panels b and d, there is no water inside the nanotube, and the system is 3D periodic. e) Comparison of Green Kubo friction coefficient (λ_GK_) for the polarizable and nonpolarizable graphene sheet and along (6, 5)-SWCNT axis.

**Extended Data Fig. 9:**
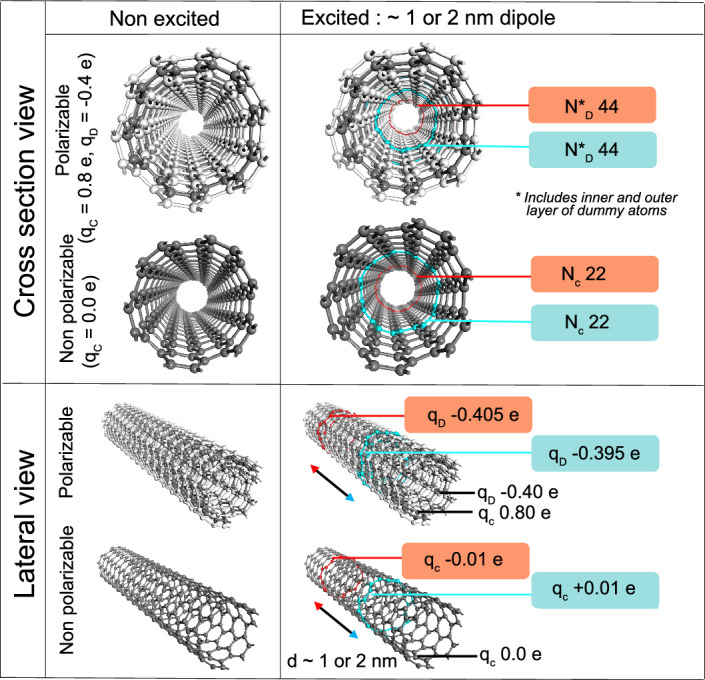
Design parameters for modelling excitons in SWCNTs. The periodic box and the water molecules are not shown to reveal the (6,5)-SWCNT and charge distribution to model the exciton in polarizable and non polarizable models with cross sectional and lateral views. N: number of atoms whose charges have been altered to model an exciton. q: charge on the altered carbon, subscript D: dummy atoms charge modified in polarizable model, subscript C: carbon atom charges modified in non-polarizable model. The double headed arrows denote the separation of 1 nm or 2 nm between the positively (red) and negatively (blue) charged annular regions. In the non-excited case or grey regions, the non-polarizable carbon atoms carry a net zero charge, whereas in the polarizable models, carbon-cores carry + 0.8 e charge. Two dummy atoms are attached to each carbon core on either side of the surface plane, an arrangement inspired by the pi-electron cloud of aromatic rings. The dummy atoms carry −0.4 e charges in the ground state to maintain charge neutrality of the overall carbon-atom (C-atom = 1 C-Core + 2 Dummy atoms). Upon excitation, the charge on the dummy atom is altered by ± 0.005 to create an exciton with the bound state of an electron and a hole pair delocalized over carbon atoms in an annular region separated by a distance d’ (d = 1 nm or 2 nm). In other words, the polarizable model creates dummy charges + 0.01 (0.005 inside and outside) at 22 carbon atoms on one side and −0.01 at 22 carbon atoms on the other side. This leads to a charge of + 0.22 e and −0.22 e at a defined distance (1 nm or 2 nm) mimicking an exciton. We chose 0.22 to account for the fact that in the experiments, there are not always excitons present (in the experiment), and the SWCNTs (in the experiments) are much longer. However, this is only a parameter and can be changed in the simulation. Note that the 22 carbon atoms represent the simplest geometry to distribute charges in a symmetric way around a (6,5)-SWCNT carbon lattice.

We next used classical molecular dynamics simulations to investigate how a delocalized electron–hole pair (that is, exciton) influences the interaction between SWCNTs and water (Fig. 4a). The (6,5)-SWCNTs are described by a new classical polarizable model^43,44^ (Extended Data Figs. 8a,b and 9 and Supplementary Table 17), which reproduces friction at the wall–water interface at the accuracy level of atomistic calculations, including electronic structure effects (Extended Data Fig. 8e). The formation of an electron–hole pair (exciton) on optical excitation is described by the addition of positive and negative delocalized charges (Extended Data Fig. 9) with a varying spatial separation of about 1 nm and 2 nm (expected size of the exciton)^14^.

These classical electrons and holes are obtained by adjusting the charges of dummy atoms attached to the carbon atoms (Drude-like oscillators that mimic the carbon π-orbitals in our model), thus creating a negative charge in the blue annular region (electron) and a positive charge in the red annular region (hole) (Fig. 4a and Extended Data Fig. 9). The dipoles interact with the surrounding water and show fluctuations of the order of ±0.45 eÅ (Fig. 4b). The dynamical nature of the excitons in the polarizable model makes a strong coupling to water possible. For comparison, a non-polarizable model was also considered (Extended Data Fig. 8c,d), without Drude-like oscillators and in which each annular region is composed of 22 carbon atoms with a charge of ±0.01 *e* each to generate a static dipole of the same size and magnitude (Extended Data Fig. 9).

**Extended Data Fig. 10:**
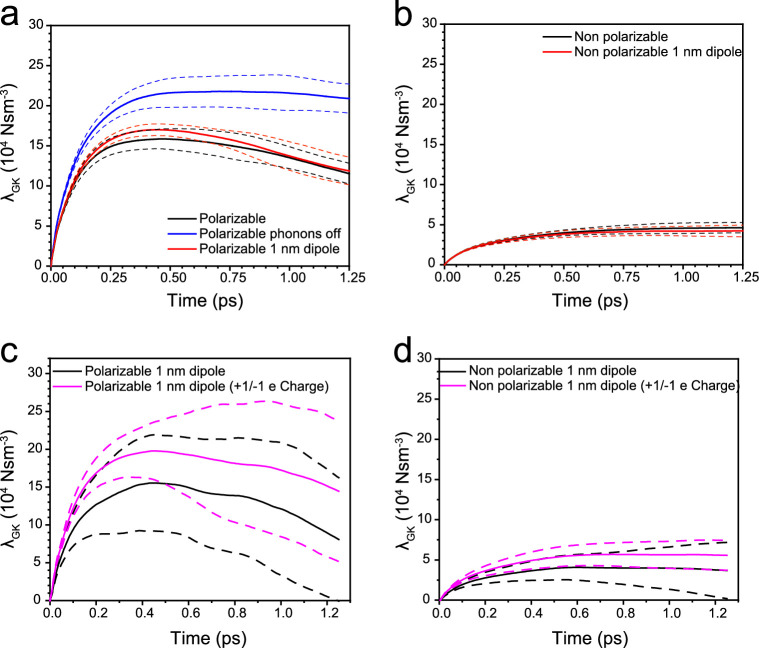
Comparison of Green-Kubo friction coefficient for polarizable and non-polarizable SWCNTs and different exciton charges. a) Friction coefficient for polarizable SWCNTs without excitation, with excitation (1 nm dipole) and with excitation (1 nm dipole, fixed carbon atoms = switched off phonons). b) Friction coefficient for non polarizable SWCNTs with (1 nm dipole) and without excitation. The average friction coefficient is depicted with solid lines, and the uncertainty region, based on the standard deviation calculated from > n = 1600 replicas, is denoted by dashed lines. In (a), (b) the excitons had a charge of ± 0.22 e. c) Friction coeffecient of polarizable and d) non polarizable SWCNTs with an exciton of ± 0.22 e (black) or ± 1.0 e (magenta) charge. Dotted lines show the uncertainty regime based on the standard deviation (n = 500 trajectories). The simulated friction coefficient in water is around four times higher for the polarizable (a) compared to the nonpolarizable model (b). The friction coefficient increases with increasing charge either in the polarizable (c) or nonpolarizable (d) model.

The coupling between the exciton dipole and the water modes results in increased friction coefficients, and they were about three to four times larger for the excited polarizable SWCNT than for the excited non-polarizable SWCNT (Extended Data Fig. 10a,b). The effects on friction did further increase (Extended Data Fig. 10c,d) when the standard exciton charge (±0.22 *e*) was increased (±1 *e*).

The simulation results indicate that a dipole is not a sufficient condition to enhance friction. The key element to couple charge dynamics to water dynamics is the dynamic nature of the exciton in SWCNTs (polarizability), resulting in enhanced friction (Extended Data Fig. 10 and Supplementary Fig. 21). In the polarizable model, the diffusion constant (Fig. 4c) decreased by > 30%. By contrast, in the non-polarizable model, the diffusion constant did not change on excitation (Fig. 4d). Notably, in experiments, the SWCNT length is much longer than that in our simulations, and there is an additional organic corona. Nevertheless, the numbers are in good agreement if we consider the length dependence of translational diffusion^45^. Given the small simulation box size (around 4 nm), the simulations cannot distinguish between local polarizability and exciton diffusion. However, the experiments with quantum defects that localize excitons (Fig. 3a) or reduce their mobility (Extended Data Fig. 5) suggest that spatial mobility or diffusivity is more important for slowing down diffusion than local polarizability. Therefore, these simplistic molecular dynamics simulations support the experimental findings and pinpoint to the parameters that slow down SWCNT diffusion on excitation. We want to point out that a full theoretical description would involve a full, rigorous description of the coupling of an exciton with the solvent fluctuations, which is a challenge that still needs to be addressed.

## Conclusion

Our study shows that light changes the diffusion of SWCNTs in water. We propose that this can be attributed at least in part to quantum friction. In this picture, exciton–water coupling generates a drag force that slows down the Brownian motion of SWCNTs. We demonstrated that either physical (light intensity) or chemical (molecules that change the fluorescence quantum yield) manipulation changes diffusion (Figs. 1 and 2). This observation points to a crucial role of the exciton concentration. Furthermore, the mobility of the exciton (Fig. 3a) and the organic corona between the carbon lattice and water (Fig. 3b) affected the magnitude of the effect. Localization and reduced exciton diffusion decrease the light-induced changes in diffusion. It suggests that the high mobility of the exciton plays a crucial part. THz OPTP measurements provided insights into the microscopic origins of this phenomenon. We identified a THz feature (30 cm^−1^) that is not present in other systems and represents the direct coupling between excitons and water^5^. It decays similarly to the expected exciton decay kinetics. Therefore, we propose that this interaction represents a pathway for quantum friction, which slows down SWCNT diffusion. Molecular dynamics simulations suggest that the polarizability of SWCNTs and the dynamic nature of the exciton dipole are central for coupling to the solvent and changes in friction (Fig. 4). Therefore, the exciton plays the central part in this effect, and the complex exciton dynamics in SWCNTs, such as thermal detrapping or dark excitons, could affect it^23,46^. Advances in engineering of exciton diffusion (for example, quantum defects) could be used to further manipulate or tailor this light-induced friction.

Our findings show that exciton-mediated friction directly affects the motion of a nanoscale object. Its mechanism is different from those known from laser traps or optical tweezers, because it is chemically tunable (Fig. 2d) and is observed for much lower laser or light intensities^47^. Thus, light-induced slowing down of Brownian motion can be physically or chemically manipulated to affect the movement of a nanomaterial in an aqueous solution. Given the anisotropy of SWCNTs, we can anticipate future approaches to control the movement of microswimmers or nanorobots. Moreover, there are fundamental implications, for example, about how light affects the kinetics of chemical reactions. The findings also raise the question of whether the effect could be present in other systems with high exciton mobility/charge fluctuations. In this work, both materials (water and SWCNTs) were freely diffusing. For immobilized SWCNTs, we could also expect light-induced effects on water transport around or through the SWCNTs with exciting potential for nanofluidics. Overall, we show that light can slow down diffusion of nanomaterials in water and that this effect is mediated by the excited-state charge fluctuations (excitons) interacting with the solvent.

## Methods

### Preparation of SWCNTs

If not stated otherwise, all chemicals were purchased from Sigma Aldrich (Germany). Unless specifically stated, all experiments were performed with (6,5)-enriched SWCNTs (Sigma Aldrich, Signis SG65i, CoMoCAT synthesis technology). For DNA-functionalized SWCNTs 150 µl of 2 mg ml^−1^ single-stranded DNA (for example, (GT)_10_) in 1× PBS buffer (pH 7.4) was mixed with 75 µl of 2 mg ml^−1^ SWCNT in PBS and 75 µl PBS, followed by tip sonication (Fisher Scientific, FB120, 120 W, amplitude 35%, 9 s pulse on and 1 s off, 15 min). The obtained solution was centrifuged for 30 min at maximum speed (21,000*g*), the supernatant was collected, and the procedure was repeated two more times. The final supernatant was stored at 4 °C until further experiments were performed. For (GT)_10_-SWCNT experiments in D_2_O, the PBS buffer was prepared with D_2_O instead of H_2_O.

The separation via aqueous two phase extraction (ATPE) of (6,4)-SWCNTs was performed according to the following protocol^48^. (DOC)-SWCNTs were mixed with polyethylene glycol (PEG) (molecular weight 6 kDa, 8% w/v), dextran (Carl Roth, molecular weight 70 kDa, 4% w/v), and the surfactants DOC (0.025% w/v), SDS (0.5% w/w) and SC (ranging from 0.5% to 0.9% w/w in 0.1% increments). The chiralities of SWCNTs in the two phases could be adjusted by adding HCl. Then, a one-step approach was used by adding a specific volume of HCl (hydrogen chloride) and NaClO (sodium hypochlorite) with 10–15% available chlorine for pH-driven and electronic separation, allowing the collection of monochiral (6,4)-SWCNTs in the bottom phase (B3). The solution was then dialysed (using a 300 kDa dialysis bag, Spectra/Por, Spectrum Laboratories) against a 1% DOC solution to remove dextran and obtain a stable 1% DOC-(6,4)-SWCNT solution.

For DOC-SWCNTs, 150 µl 2% (m/v) DOC in H_2_O was mixed with 150 µl of 2 mg ml^−1^ SWCNTs (in H_2_O), followed by tip sonication and centrifugation similar to the conditions for (GT)_10_-SWCNTs preparation. The acquired supernatant was stored at 4 °C. SDBS- and SC-functionalized SWCNTs were prepared according to the same procedure as DOC-SWCNTs.

Quantum defect introduction was performed according to a previously developed protocol^49^. Briefly, 20 µl of 4 mM 4-nitrobenzol diazonium tetrafluoroborate diazonium salt (dissolved in water) was added to 20 ml of 10 nM SDBS-SWCNTs solution. Then the mixture was irradiated with green light (550 nm) while stirring for 15 min. The obtained solution was mixed with the same volume of acetonitrile (ACN) and, consequently, the SWCNTs precipitated. The pellet was then washed with H_2_O two or three times to remove residual SDBS and ACN. Finally, the acquired precipitate was redispersed in 1% DOC by 15 min tip sonication followed by centrifugation for 30 min at 21,000 *g*. The collected supernatant was used for the experiments. The length of SWCNTs prepared by this procedure is about 600 nm (ref. ^25^).

All samples were colloidally stable in aqueous solution without signs of aggregation as confirmed by absorbance (Extended Data Figs. 1a and 2, and Supplementary Fig. 1), one-dimensional (1D) (Extended Data Fig. 1b,c) and two-dimensional (2D) fluorescence spectroscopy (Supplementary Figs. 2 and 3) and atomic force microscopy (average SWCNT length of around 600 nm; Supplementary Figs. 4 and 5). We also prepared chirality-pure (6,5)- and (6,4)-SWCNTs to exclude effects from impurities (Extended Data Fig. 2 and Supplementary Fig. 1).

### NIR spectroscopy

#### One-dimensional fluorescence spectra

One-dimensional spectra of 0.5 nM (GT)_10_-SWCNTs with or without analytes (2 μM riboflavin and 100 μM ascorbic acid in aqueous solution) or 0.5 nM DOC-, SC- and SDBS-functionalized SWCNTs were measured in a custom-built setup based on an Olympus IX73 microscope and a solid-state laser (Quantum gem-561, 561 nm). The emission spectra were captured with an Andor iDus InGaAs 491 array NIR detector coupled to a Shamrock 193i spectrometer (Andor Technology).

#### Two-dimensional fluorescence spectra

The same setup as for 1D spectra was used. However, to obtain 2D excitation–emission spectra of 2 nM SWCNTs in various surfactants and (GT)_10_-SWCNTs in 1 × PBS (D_2_O) at pH 7.4, a monochromator (MSH150) equipped with an LSE341 light source (LOT-Quantum Design) was used for tunable excitation.

#### FCS measurements

FCS measurements were performed with a MicroTime 200 system (PicoQuant), equipped with pulsed lasers at 485 nm (LDH-C-D-485) and 530 nm (LDH-D-TA-530), an Olympus IX73 inverted confocal laser scanning microscope equipped with a 60× water objective (Olympus, numerical aperture 1.2, UPlanSApo), and single-photon avalanche photodiodes (SPADs) detectors (Excelitas Technologies). We focused on (6,5)-SWCNTs because of the limited sensitivity of the detectors in our FCS setup in the NIR region >1,100 nm. Samples at a concentration of 1 nM were excited with a pulsed laser at 485 nm, operating at a frequency of 40 MHz. DOC-(6,4)-SWCNTs showed weak emission when excited at 480 nm. Consequently, we used the 532 nm excitation. As the 532 nm laser could not achieve higher power levels in pulsed mode, we used CW excitation at 532 nm for this measurement, ensuring that the excitation power remained consistent. The emitted light was separated from the excitation light through a dichroic mirror (R405/488/532/635, Semrock), passed through a 900-nm long-pass filter (Thorlabs) to block the excitation light, and then focused onto a 50-μm pinhole and directed to the SPAD detectors. For DOC-(6,4)-SWCNTs, a 800-nm long pass filter (Thorlabs) was used. The refractive index and viscosity corrections were done by adjusting the collar settings^50^.

The autocorrelation function of the fluorescence intensity *I* is defined as

1 $$G(\tau )=\frac{\langle I(t)I(t+\tau )\rangle }{\langle I{(t)}^{2}\rangle }$$

*G*(*τ*) correlates the fluctuation of the intensity of a fluorophore at time *t* and after time lag *τ*.

Fluctuations arise because of the diffusive motion of the fluorophore through the 3D Gaussian confocal volume having widths *w*_*z*_ and* w*_*xy*_. The correlation function corresponding to the diffusion is

2 $${G}_{D}({\tau })=\frac{1}{N}{\left[1+\frac{{\tau }}{{{\tau }}_{D}}\right]}^{-1}{\left[1+\frac{{\tau }}{{w}^{2}{{\tau }}_{D}}\right]}^{\frac{-1}{2}}$$

where *N* is the total number of molecules in the confocal volume and *τ*_*D*_ is the diffusion time of that system. It is linked to the diffusion constant *D* by

3 $${\tau }_{D}=\frac{{w}_{{xy}}^{2}}{4D}$$

The structural parameter $w=\frac{{w}_{x}}{{w}_{{xy}}}$ was calibrated using the known Atto 488 dye (1 nM) in water (*D*_t_ = 400 μm^2^ s^−1^) (ref. ^51^). The calculated excitation volume was 1.5 fl.

To analyse the FCS data, the software Igor Pro 6.34 A and the following equation was used for fitting:

4 $${G}_{D}(\tau )=\frac{1}{N}{\left[1+\frac{\tau }{{\tau }_{D}}\right]}^{-1}{\left[1+\frac{\tau }{{w}^{2}{\tau }_{D}}\right]}^{\frac{-1}{2}}\,\left[1+\frac{T}{1-T}\exp {\left(-\frac{\tau }{{\tau }_{t}}\right)}^{\beta }\right]$$

where *T* is the fraction of the fluorescent molecules in the dark state and ${{\tau }}_{{t}}$ signifies the corresponding lifetime. The stretching exponent *β* is a marker for the degree of heterogeneity in the associated dynamics^52^.

#### FCS control experiments

A control experiment under identical conditions was conducted with the dye Atto 488 (Supplementary Fig. 6a) and showed a slight decrease in *G*(0) value but no change in the normalized autocorrelation functions and the diffusion time (Supplementary Fig. 6b) under the same experimental conditions. This control experiment rules out effects from sample heating, which is known for surface-immobilized emitters^53,54^. Furthermore, we verified that the temperature of the samples remained constant for both 10 μW and 90 μW (Supplementary Fig. 9). Brightness of (GT)_10_-SWCNTs also increased linearly with laser power (Supplementary Fig. 7a), indicating the absence of non-linear effects such as exciton–exciton annihilation. The increase in the number of (apparent) fluorescent particles (Fig. 1b) from 3 to 9.3 in the confocal volume with higher laser power (Supplementary Table 1) can be attributed to the relatively small quantum yield of NIR fluorophores such as SWCNTs (refs. ^55,56^), which means that they are not saturated by excitation. Moreover, the diffusion behaviour of (GT)_10_-SWCNTs could be reversibly switched by changing the excitation power (Supplementary Fig. 7b). We also performed FCS measurements of chirality-purified (GT)_10_-(6,5)-SWCNTs and found that the diffusion behaviour was similar to that of the normal (GT)_10_-SWCNTs (Supplementary Fig. 8 and Supplementary Table 2), which shows that sample purity is high in all cases and does not affect diffusivity.

Although most FCS experiments employed pulsed excitation to collect more information (for example, lifetime), FCS using CW excitation showed the same results (Extended Data Fig. 7a,b and Supplementary Table 14). This finding suggests that the diffusion behaviour of SWCNT (on the ms timescale) is less affected by the excitation timing (ps timescale) but rather the overall absorbed energy.

As a control, to assess whether analytes induce aggregation or dissociation of SWCNTs, we measured the absorbance spectra. They remained unchanged for both analytes (Supplementary Fig. 13). Thus, chemical manipulation affects diffusion the same way it affects exciton concentration (quantum yield). These results allow to exclude that the change in diffusion is an optical artefact. It is distinct from trapping of objects by light with optical tweezers^57^.

We also investigated a 80% glycerol/water mixture and observed almost no changes in diffusion (Extended Data Fig. 3b and Supplementary Table 4). By contrast, for a 20% glycerol/water mixture, we observed power-dependent changes in the diffusion constant similar to those of water (Extended Data Fig. 3c and Supplementary Table 5). Previous THz spectroscopic studies demonstrated that when the glycerol concentration is below 20%, the number of water molecules hydrogen-bonded to glycerol continues to increase. By contrast, at concentrations above 40%, the number of hydrogen-bonded water molecules decreases because of the overlap of hydration shells^58^.

To further understand the entanglement of excitation and diffusion in the FCS geometry, the random walk of SWCNTs in a box with a confocal laser volume was simulated (Supplementary Fig. 22). The results (Supplementary Fig. 23) qualitatively confirmed that changes in the diffusion by excitation of excitons in moving and rotating SWCNTs lead to the power-dependent changes of the autocorrelation functions observed in the experiments (Fig. 1).

#### THz measurements

The OPTP spectrometer was described in detail previously^36^. In summary, the system uses 50 fs, 800 nm laser pulses generated by a Ti:sapphire-amplified laser to produce a broadband THz probe pulse using a two-colour air plasma filament^59^. Part of the 800-nm-wavelength laser radiation is frequency-doubled in a BBO (beta barium borate) crystal to generate 400 nm light, which serves as the optical pump. We measure the changes in THz absorption on optical excitation as a function of pump–probe delay, Δ*t*, between 0.25 ps and 300 ps by using a mechanical delay stage. To eliminate interference effects and the excitation of free charge carriers, we used a windowless, free-flowing jet with a thickness of 20 μm as the sample^36^. An 80 ml solution of SWCNTs (about 100 nM) was circulated in the jet for 96 h. A defoaming agent (BYK 025) was added to the reservoir to prevent foam generation. This defoaming agent remains as a thin film on the surface and does not interfere with the sample measurements. The THz field is detected using electro-optic sampling with a 100 µm thin gallium phosphide (GaP) crystal. For further analysis, the electric fields are Fourier-transformed. The difference in THz transmission before and after optical excitation is expressed as ΔmOD. Positive values indicate a decrease in transmission on optical excitation. The fluence of the blue light was varied from 50 mJ cm^−2^ to 120 mJ cm^−2^ and then to 200 mJ cm^−2^. As a reference, we also measured pure water at a fluence of 200 mJ cm^−2^. In the plots, we show data for a fluence of 200 mJ cm^−2^, unless stated otherwise.

#### Wide-field tracking of SWCNTs

A 2.3 µm Mylar thin film (TF−125-225-F from Fluxana) was used as a spacer and placed between two glass cover slides to create a narrow flow-chamber-like volume. Subsequently, 50 µl of a 0.1 nM purified DOC-SWCNT solution was added. Single-walled carbon nanotube (SWCNT) tracking was performed using a custom-built setup. A 561 nm laser (Cobolt Jive 500, 200 mW, 100 W cm^−^^2^) was coupled to an Olympus IX73 microscope equipped with a 100× (UPlanSApo 100×/1.35 Sil, Olympus) oil-immersion objective. Imaging in the NIR was performed with a InGaAs camera (Cheetah, Xenics 640, 640 × 512 pixel, thermoelectrically cooled). A dichroic mirror (VIS/NIR, HC BS R785 lambda/5 PV, F38-785, AHF) and a 900 nm long-pass filter (FELH0900, Thorlabs) were installed in the beam path between the objective and the cameras. The NIR images were typically acquired at 7 frames per second (fps) with a 140 ms exposure time. All analyses were conducted using Python 3.10.5. For particle tracking, the Python library trackpy was used to identify bright spots corresponding to individual SWCNTs. We analysed only traces above a certain length (typically 100 frames). The analysis determines the *x* and *y* centre-of-mass coordinates of particle positions. From the trajectories, we calculated the ensemble time-averaged mean squared displacement (MSD)^60^.

#### Computation of friction and diffusion in water

The classical atomistic molecular dynamics simulations were run with an open source LAMMPS (Large-scale Atomic/Molecular Massively Parallel Simulator)^61^ to estimate the interfacial friction coefficient and diffusion of graphene and (6,5)-SWCNTs in explicit water. The models for graphene slab (2.5 × 2.6 nm^2^) with 1,600 water molecules and (6,5)-SWCNTs (3 × 3 × 4.1 nm^3^) with 1,100 water molecules systems were created in Material Studio^62^. For the calculation of the interfacial friction, the graphene system used is periodic in the *x*–*y* directions and non-periodic in the direction perpendicular to the surface, whereas all the CNT systems are 3D periodic with an infinite nanotube along the axial direction. Both non-polarizable and polarizable systems are analysed with harmonic consistent valence forcefield (CVFF)^63^ and interface force field-CVFF (IFF-CVFF)^64^ parameters, respectively (Supplementary Table 17), which use 12-6 LJ potential for the van der Waals interactions. In the non-polarizable model, the carbon atom C is neutral and only has LJ interactions with the water, whereas in the polarizable model, each carbon is decorated with two flexible negatively charged dummy atoms that mimic the π-orbitals and are perpendicular to the plane of C atoms. The dummy atoms are connected by harmonic bonds and angle restraints (Supplementary Table 17 for parameters). A similar simple model to include the metal polarization, which consists of a LJ potential and a harmonically coupled core–shell charge pair for every atom has been recently developed and proved to reproduce the classical image potential of adsorbed ions as well as surface, bulk and aqueous interfacial properties in agreement with experiments^44^. Here, two layers of virtual atoms sandwich the carbon layer in between to form a single graphene sheet or SWCNTs (Extended Data Fig. 8a,b). The dummy atoms mimic the π-electron cloud and add polarizability to the carbon atoms^43^. The polarizable carbon carries a partial positive charge (+2*δ*) and the two dummy atoms carry a negative half charge (−*δ*), so the overall C atom is neutral. However, there is an additional dipole contribution to each C atom. Hence, polarizable graphene/SWCNTs also have a columbic interaction with the surrounding water.

The Green Kubo (GK) friction coefficient has been calculated to estimate the strength of the interfacial interaction of water with the graphitic surfaces^65,66^ (Extended Data Fig. 10e), according to the formula

5 $${\lambda }_{\mathrm{GK}}=\frac{1}{{An}{K}_{{\rm{B}}}T}{\int }_{0}^{\infty }\langle \mathrm{FL}({\rm{t}})\mathrm{FL}(0)\rangle $$

where *A* is the area of the surface, *n* is the number of dimensions (*n* = 2 for graphene and 1 for CNT), *K*_B_ is the Boltzmann constant, *T* is the temperature and FL is the lateral force acting on the surface for graphene or the force along the axial direction for the CNT. The integral of the autocorrelation of FL is used to compute the GK friction coefficient as per equation (5). The friction coefficient for non-polarizable graphene and (6,5)-SWCNT was computed with the CVFF parameters, and for polarizable graphene and (6,5)-SWCNTs was computed with the IFF-CVFF polarizable model^44,64^.

We observe a higher friction coefficient of around 6.5 × 10^4^ N s m^−^^3^ at the graphene interface with the polarizable model as compared with 2 × 10^4^ N s m^−^^3^ for non-polarizable graphene, which is also the typical value observed with other force fields^4^. Notably, the value for the friction coefficient obtained with our polarizable model is in very good agreement with the ab initio estimates of 4.5 × 10^4^ N s m^−^^3^ (ref. ^66^) and 9.5 × 10^4^ N s m^−^^3^ (ref. ^67^) obtained with revPBE-D3 and optB88-vdw functional, respectively (Extended Data Fig. 8e). We also observed that the friction coefficient increases for water in contact with the external surface of SWCNTs from 6.5 × 10^4^ N s m^−^^3^ for the non-polarizable model, to 15 × 10^4^ N s m^−^^3^ for the polarizable model. The result for the polarizable model is in good agreement with ab initio molecular dynamics results from a previous study^67^. Hence, the new polarizable model permits reproducing electronic structure level accuracy at the cost of simple classical force field simulations, introducing the interaction of the polarizable electron cloud with the polar solvent. With the new and improved IFF-CVFF polarizable model, we also estimated the diffusion behaviour of (6,5)-SWCNTs. The latest IFF-CVFF polarizable graphite model (Supplementary Table 17) has been validated with rigour by reproducing bulk properties such as density and bulk modulus, and interfacial properties such as surface energy, hydration energy and water contact angle, which are in excellent agreement with experimental observations and are suitable for model graphitic materials in various applications.

The diffusion constant was computed with a (6,5)-SWCNT of length 4.1 nm placed inside a cubical 3D periodic box of 140,000 water molecules modelled with flexible SPC parameters (CVFF). After pre-equilibration of the simulation box in an isothermal–isobaric (NPT) ensemble, the simulation trajectory was run for another 20 ns with a timestep of 0.5 fs, and a coordinate snapshot was generated every 1 ps. The (6,5)-SWCNT was end-capped with hydrogen atoms and allowed to diffuse inside the box unconstrained with the NPT ensemble at 298 K and 1 atm. Hydrogen parameters are borrowed from the CVFF models. The trajectory was analysed to compute the MSD of the centre of mass of the SWCNT with time. The slope (*m*) of the MSD compared with time plot was used to evaluate the diffusion constant *D* = *m*/6. The diffusion constant was calculated for both non-excited and excited SWCNTs.

The process of exciting the SWCNT, in molecular dynamic simulations with a classical potential, is modelled by introducing an exciton by the addition of an axial dipole along the SWCNT axis, as described in the main text. The charges of 44 virtual/dummy (pi cloud) atoms are modified by ± 0.005 *e* so that the total charge is 0.22 *e* (44 virtual sites × 0.005 *e* per virtual site) in the polarizable case, whereas, for the non polarizable SWCNT model, the annular each region is composed of 22 carbon atoms with a charge of ± 0.01 *e* each to generate the excited state nanotube (Extended Data Fig. 9). This initial choice was motivated by introducing a moderate perturbation to maintain the stability of the simulation system and avoid crashing by overpolarization. Exact ± 1 would correspond to exactly one exciton present always throughout the experiment. However, smaller values are more likely because of lower exciton density (due to the average of excited and non-excited time periods in pulsed as well as CW excitation schemes and the longer SWCNT length in experiments). We also investigated the friction coefficient for dipole-free excitation of the SWCNT (Extended Data Fig. 10, Supplementary Fig. 21), in which the two outer rings (blue) carry an additional −0.005 *e* charge on the 44 atoms in each ring, behaving as a delocalized electron. The delocalized hole is modelled using a central ring (red) composed of 44 atoms, each carrying an extra charge of + 0.01 *e*. In this charge configuration, the SWCNT dipole moment is negligible compared with the excited configuration shown in Extended Data Fig. 9, in which two rings describe a delocalized exciton. The polarizable nature of the electron cloud and excitons is analysed using molecular dynamics. The dynamic nature is captured mildly by separate simulations with 1 nm and 2 nm dipoles, then averaging the MSD. The true translational nature of the exciton along the length of the SWCNT is beyond the scope of molecular dynamics runs, as these studies require (1) extremely long SWCNTs that are hundreds of nanometres long and (2) a special implementation of varying charges with time on atoms in standard simulators such as LAMMPS, both of which are beyond the scope of this work.

## Online content

Any methods, additional references, Nature Portfolio reporting summaries, source data, extended data, supplementary information, acknowledgements, peer review information; details of author contributions and competing interests; and statements of data and code availability are available at 10.1038/s41586-026-10632-2.

## Supplementary information

Supplementary Information This file contains Supplementary Figs. 1–23, Supplementary Tables 1–17, Supplementary Methods (Particle Tracking and FCS Simulation) and additional references.

Peer Review File

## Data Availability

All code and data are publicly available at 10.17877/RESOLV-2025-M8SI9UCF.
