# Supplementary material for: Light-induced quantum friction of carbon nanotubes in water
Source: Nature. 2026 Jun 10;654(8120):941–7. doi: 10.1038/s41586-026-10632-2 (PMC13293881; doi:10.1038/s41586-026-10632-2)
Supplement: Supplementary file 1 — This file contains Supplementary Figs. 1–23, Supplementary Tables 1–17, Supplementary Methods (Particle Tracking and FCS Simulation) and additional references. [file 41586_2026_10632_MOESM1_ESM.pdf]

nature portfolio

https://doi.org/10.1038/s41586-026-10632-2

Supplementary information

Light-induced quantum friction of carbon nanotubes in water

In the format provided by the
authors and unedited

Supplementary information

Light-induced quantum friction of carbon nanotubes in water

Tanuja Kistwal[1,4+], Krishan Kanhaiya[3,5+], Adrian Buchmann[1], Chen Ma[1], Jana Nikolić[1], Julia Ackermann[2], Phillip Galonska[1], Sanjana S. Nalige[1], Vahideh Sardari[1], Aishwarya Sudarsan[1], Martina Havenith[1], Marialore Sulpizi[3*], Sebastian Kruss[1,2*]*

[1] Department of Chemistry and Biochemistry, Ruhr-University Bochum, Universitätsstraße 150, 44801 Bochum, Germany.

[2] Fraunhofer Institute of Microelectronic Circuits and Systems, 47057 Duisburg, Germany.

[3] Department of Physics and Astronomy, Ruhr-University Bochum, Universitätsstraße 150, 44801 Bochum, Germany.

[4] Department of Chemistry, Amity Institute of Applied Science, Amity University, Noida, 201303 Uttar Pradesh, India

[5] Department of Chemical Engineering, Indian Institute of Technology, Hyderabad, 502284, Telangana, India

+ These authors contributed equally

* Correspondence: sebastian.kruss@rub.de; Marialore.Sulpizi@rub.de; martina.havenith@rub.de

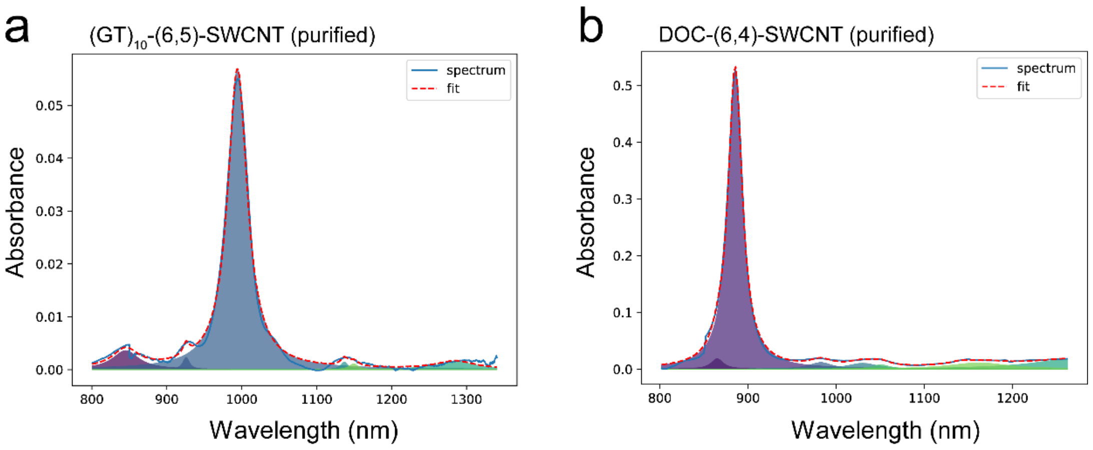

Figure S1. Fitted absorbance spectra of chirality enriched SWCNT samples (E_{11} region). *a) $(GT)_{10}$-(6,5)-SWCNTs b) DOC-(6,4)-SWCNTs. The fit is based on multiple Lorentzians and minimizing the difference to the data.*

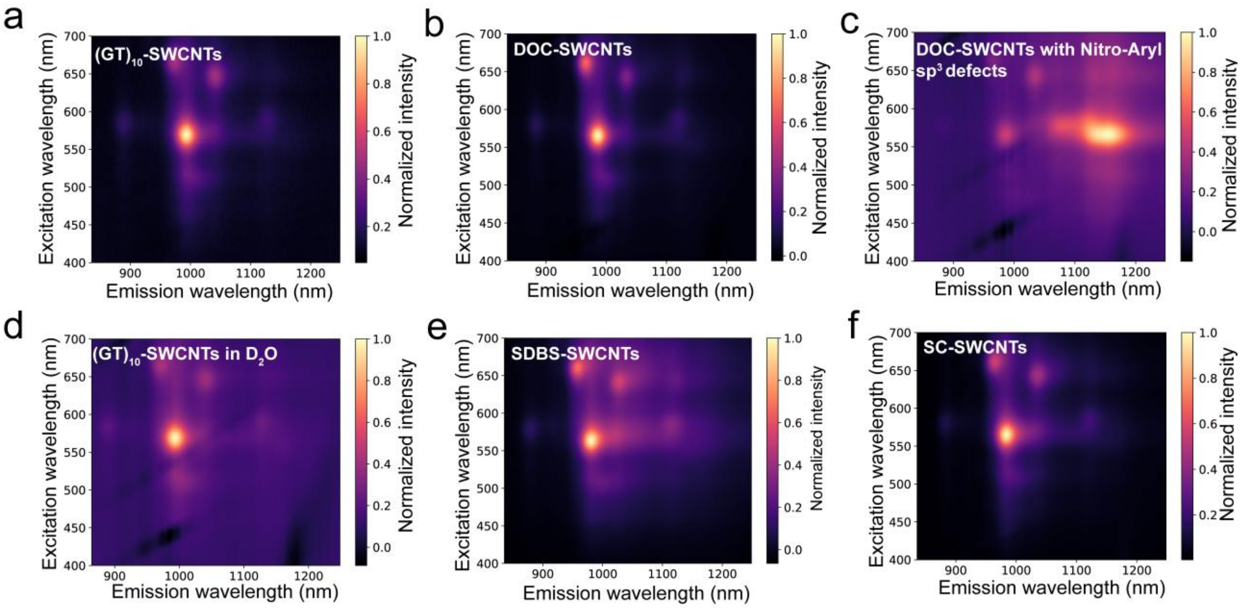

Figure S2. 2D excitation-emission spectra of SWCNT samples. *a) $(GT)_{10}$-SWCNTs, b) DOC-SWCNTs, c) DOC-SWCNTs with Nitro-Aryl sp^3 quantum defects, d) $(GT)_{10}$-SWCNTs in D_2O based PBS, e) SDBS-SWCNTs, f) SC-SWCNTs. The concentration of SWCNTs was 2 nM.*

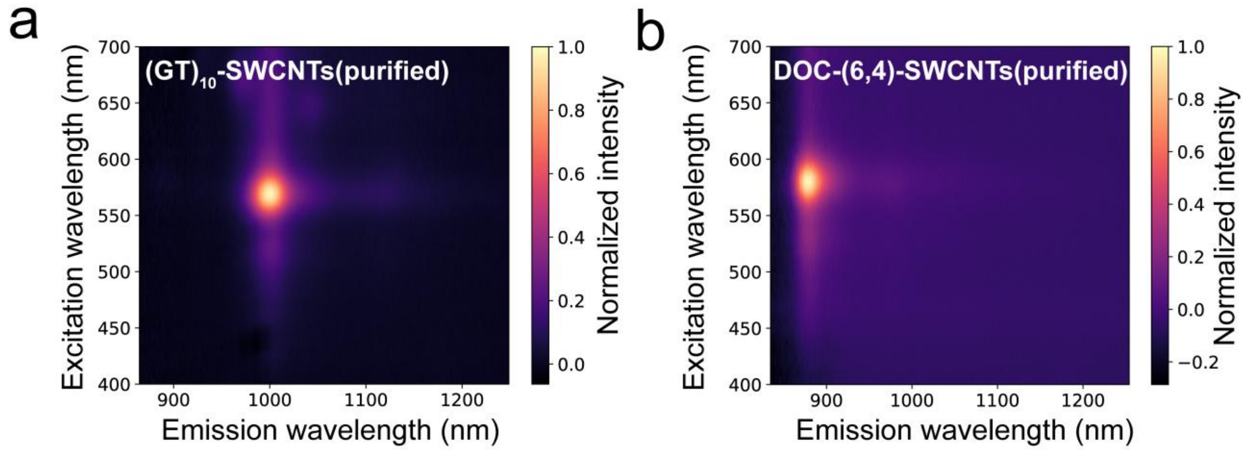

Figure S3. 2D excitation-emission spectra of chirality enriched SWCNT samples. *a) $(GT)_{10}$-(6,5)-SWCNTs. b) DOC-(6,4)-SWCNTs.*

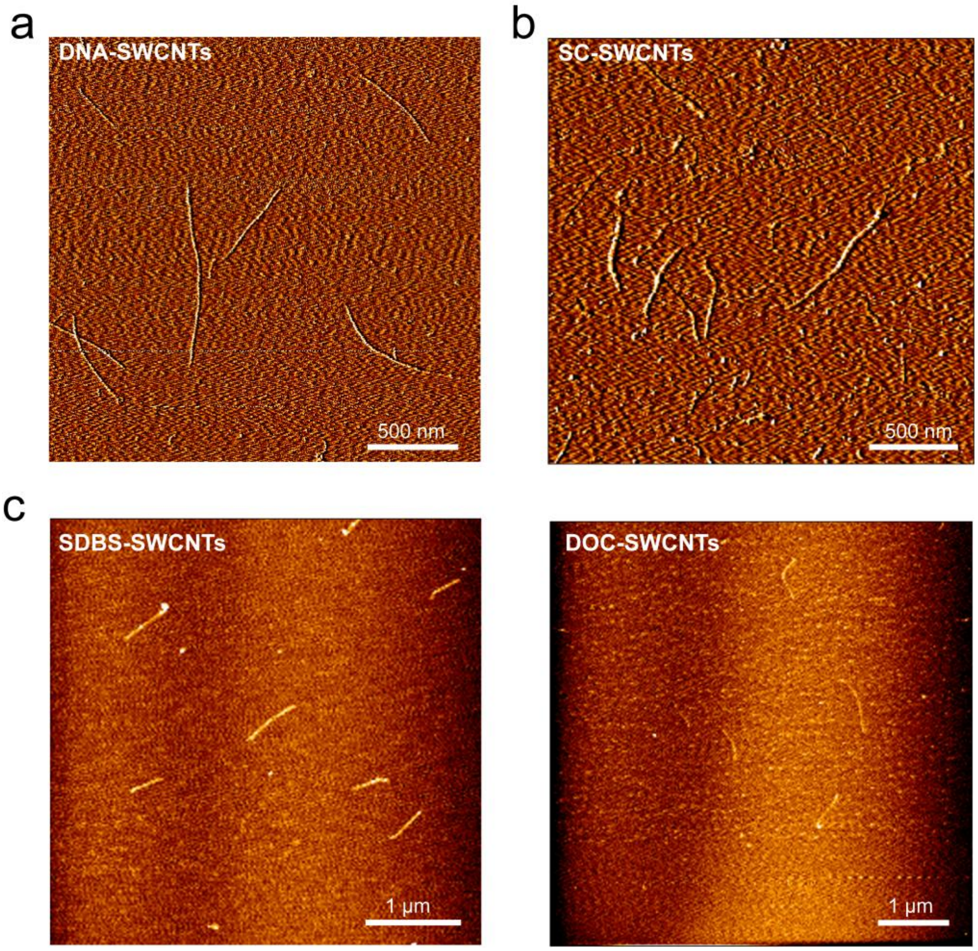

Figure S4. AFM (Atomic force microscopy) images of SWCNTs. *a) $(GT)_{10}$-SWCNTs, b) SC-SWCNTs, c) SDBS-SWCNTs, d) DOC-SWCNTs. Note that images a and b were captured using the AC tapping mode (Amplitude Modulation Tapping Mode) of the AFM, while images c and d were taken using the Qi (Quantitative Imaging Mode) measurement mode of the AFM, and SWCNTs (2 nm) are spin-coated onto the mica surface and then washed with water.*

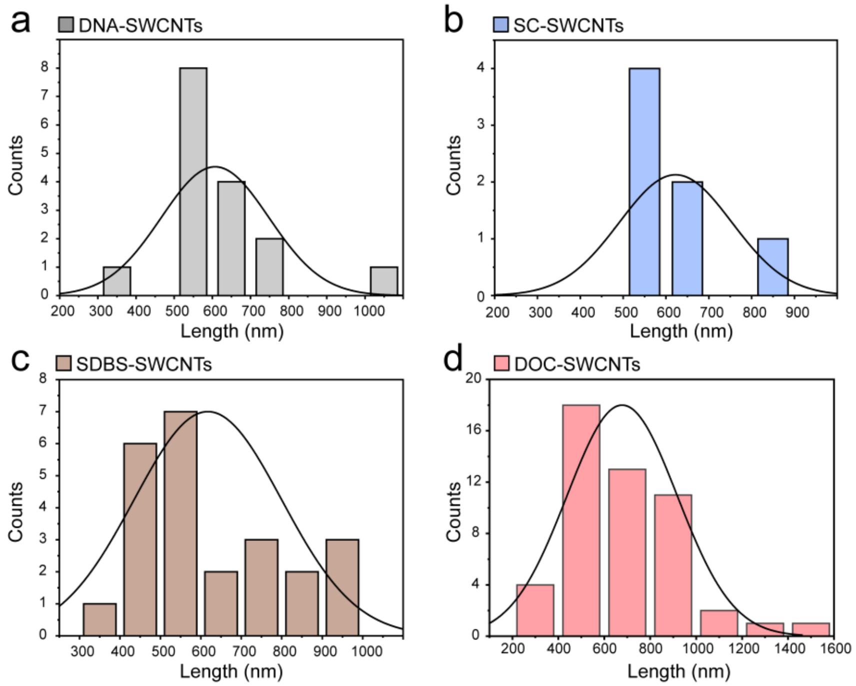

Figure S5. Length distribution of SWCNTs measured by AFM. *a) $(GT)_{10}$-SWCNTs (mean length 607 ± 140 nm), b) SC-SWCNTs (mean length 622 ± 131 nm), c) SDBS-SWCNTs (mean length 617 ± 180 nm), d) DOC-SWCNTs (mean length 677 ± 240 nm). The blank solid lines indicate Gaussian fits that were used to extract the mean ± SE for each distribution. ~ 30 particles are measured for each sample. Length was determined manually in Image J software.*

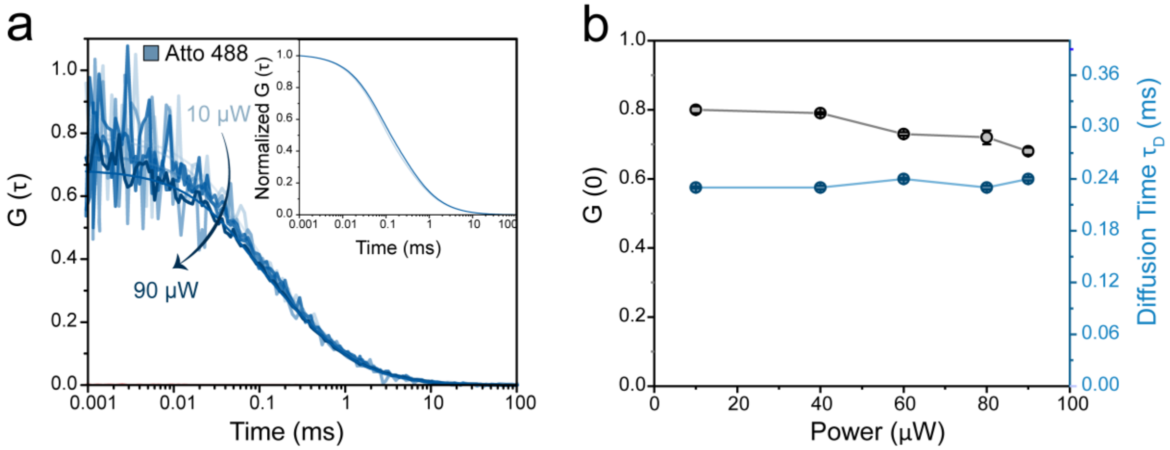

Figure S6. FCS control experiment with Atto 488 dye. *a) Fluorescence correlation curves of Atto 488 dye (1 nM) in water with increasing excitation power. b) Variation of initial correlation amplitude G (0) and diffusion time (τ_D) as a function of excitation power (n=3, mean ± SD).*

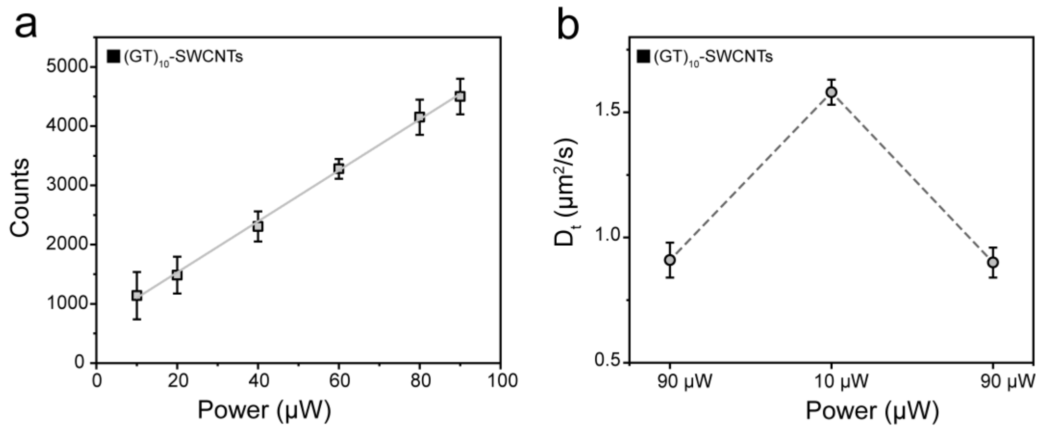

***Figure S7. Fluorescence intensity scales linearly with excitation power and the change of the diffusion constant is reversible**. Molecular brightness of $(GT)_{10}$-SWCNTs (1 nM) as a function of excitation power (Microtime 200 setup, λ_{exc}=480 nm), and fitted by a linear equation (grey) (R^2 = 0.997) (n=3, mean ± SD) b) The diffusion constant of $(GT)_{10}$-SWCNTs can be reversibly changed with excitation power (dashed grey lines show a visual representation) (n=3, mean ± SD).*

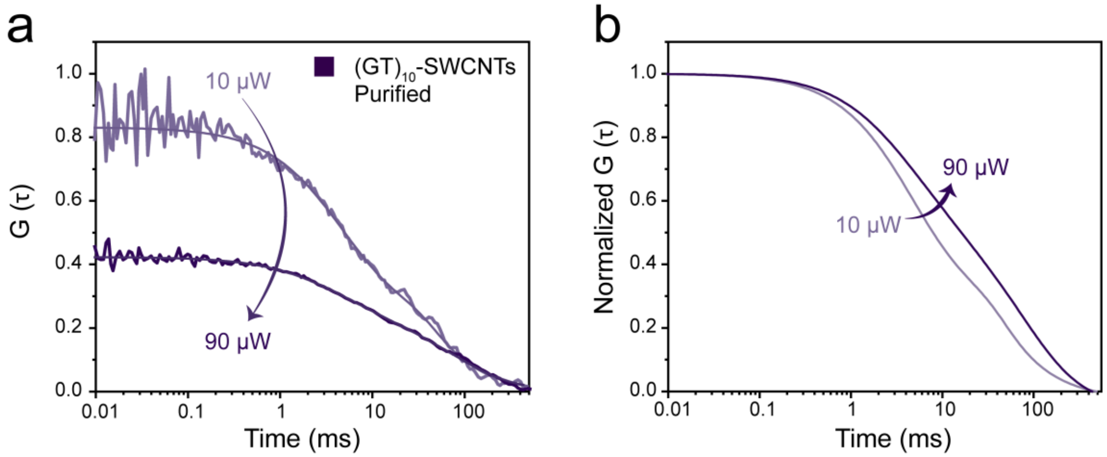

***Figure S8. Influence of excitation power on fluorescence autocorrelation functions of purified $(GT)_{10}$-(6,5)-SWCNTs**. a) Autocorrelation and b) Normalized autocorrelation of purified $(GT)_{10}$-SWCNTs with 10 µW and 90 µW.*

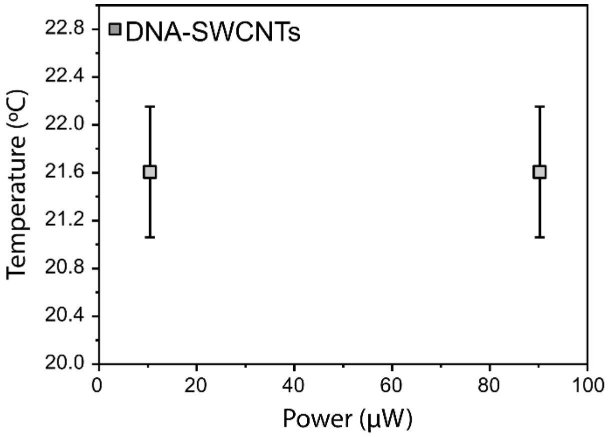

***Figure S9*. Temperature remains constant across power levels:** *A comparison of temperature change at 10 µW and 90 µW laser power in (GT)₁₀-SWCNT samples in PBS (n=5, mean ± SD). Temperature sensors recorded the temperature before and after the experiment under two distinct powers.*

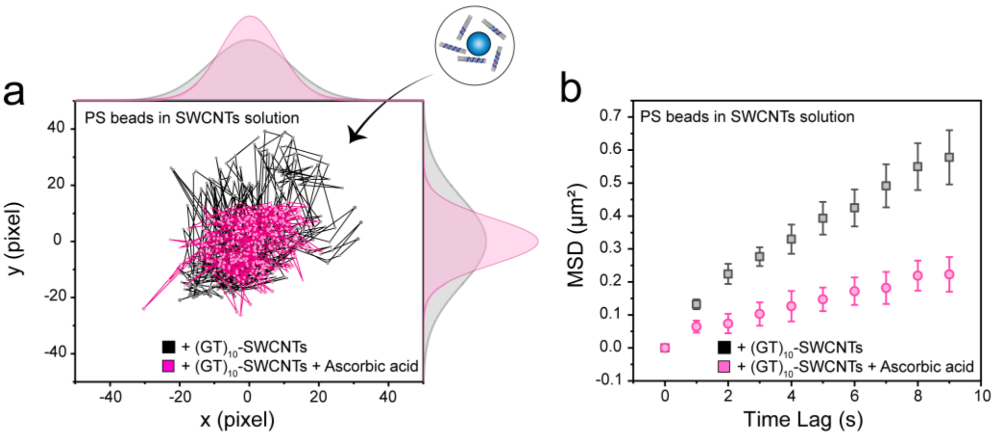

Figure S10. Macroscopic viscosity changes by quantum friction. *a) Single particle trajectories of (fluorescent) polystyrene (PS) beads (5 µm) diffusing in a PBS buffer solution of (GT)₁₀-SWCNTs with and without ascorbic acid (100 µM), which increases exciton concentration (see Figure 2a). Gaussian histograms show the distribution of x and y positions. b) Ensemble time-averaged MSD plots for PS beads indicate slower diffusion in the presence of more excitons (+ascorbic acid) (mean ± SE, n=4 independent experiments with > 20 PS beads each).*

Tracking of rhodamine B labeled polystyrene beads in a SWCNT solution

5 µm PS beads (Sigma Aldrich product number: 79633) were used for particle tracking. First, 100 µL of the solution containing 1 part PS beads to 9 parts PBS 200 was pipetted out. Next, 30 µL of RhB dye (Sigma-Aldrich, CAS No: 81-88-9) was pipetted from a freshly prepared stock solution (1 mM) and added to the PS beads solution. Following this, 770 µL of PBS was added, bringing the final volume of the sample to 1 mL. After vortexing the mixture, the sample was incubated in the dark for 1 hour to allow the dye to adsorb onto the beads. The next step involved washing out the excess dye from the suspension by centrifuging the sample twice for 5 minutes each time.

For particle tracking microscopy, we used the Leica THUNDER Imager 3D Cell Culture & Infinity Laser Scanner, employing fluorescence as the contrast method with an excitation wavelength of 575 nm and filter cube CYR7101. A 63x oil immersion objective was utilized for imaging. The sample was placed in a glass petri dish, which was then covered with a glass slide. The total sample volume was 200 µL, consisting of 186 µL of RhB PS bead solution and 14 µL of 70 nM $(GT)_{10}$-SWCNTs with or without 1 µL of 20 mM ascorbic acid. Images were captured at a frame rate of 10 frames per second. After collecting the videos, we processed the data using a Python script based on the trackpy package. From the trajectories, we then calculated the ensemble time-averaged mean squared displacement (MSD).

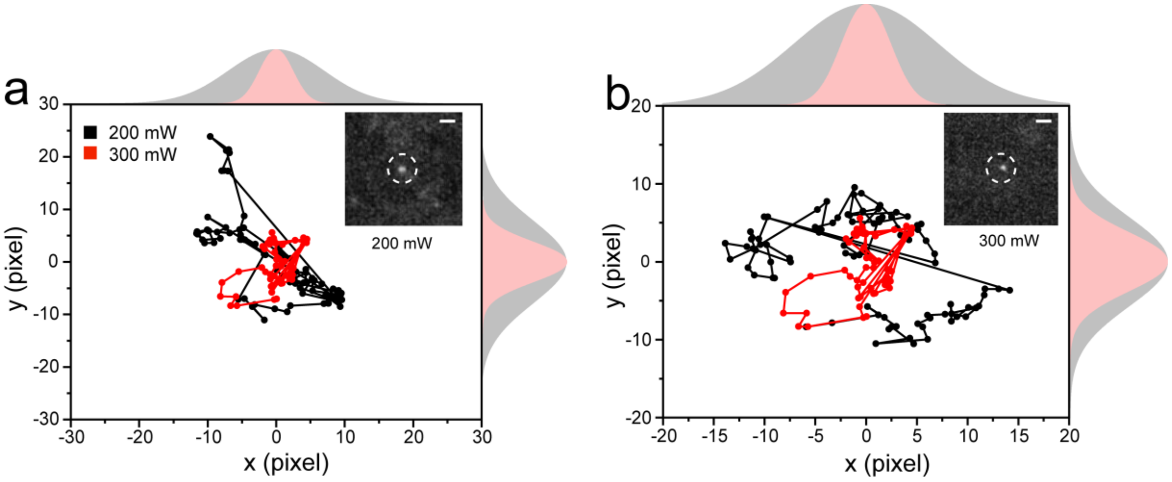

Figure S11. Wide field single SWCNT tracking. *a,b) Exemplary single-particle trajectories of DOC-SWCNTs diffusing in an aqueous solution with 200 and 300 mW laser excitation in a wide field microscopy setup. Gaussian functions are used to fit the position histograms (Scale bar: 1 µm). The inset shows the image of the single SWCNT (dashed circle).*

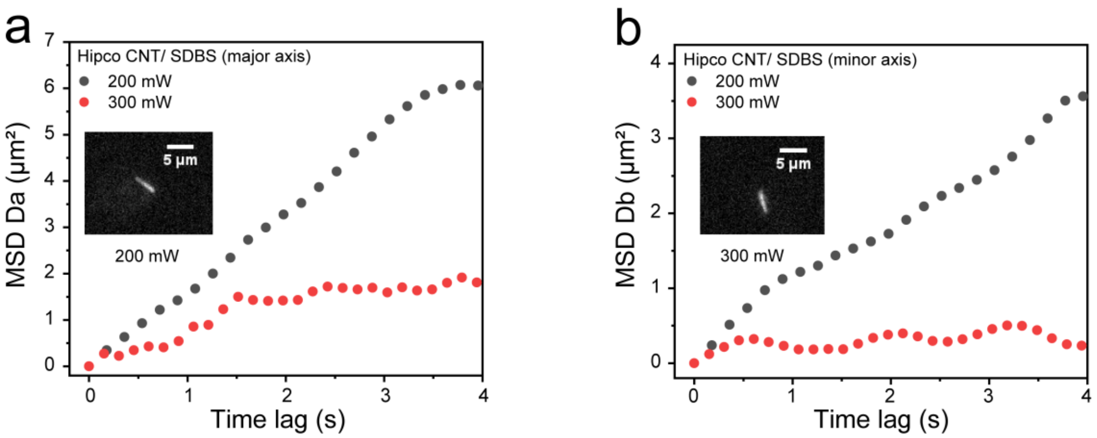

***Figure S12. Anisotropic diffusion of HiPCO SWCNTs under varying excitation power**. a) Time average mean square displacement (TA-MSDs) of a 4.4 µm HiPco SWCNT in 0.5 % SDBS under linearly polarized excitation at 200 mW and 300 mW. a) TA-MSDs along major axis and b) TA-MSDs along minor axis diffusion. The insets show images of the SWCNTs. The center of mass was identified by fitting an ellipsoid to the SWCNT. Undulations of the SWCNT also affect the analysis of the trajectories.*

For particles of size above the resolution limit the analysis required adjustments. For example, any moving anisotropic particle will have at least two translational diffusion constants: one along the principal body axis (D_a) and another perpendicular to it (D_b). Furthermore, localization of such asymmetrical particles necessitates adopting the assumption of a diffusing ellipsoid in a two-dimensional context and extracting the centroid positions (x, y) of this ellipsoid. The standard laboratory frame coordinates (x, y) and conventional mean squared displacements (MSDs) do not capture the true anisotropic diffusion present in cylindrical particles.[1]

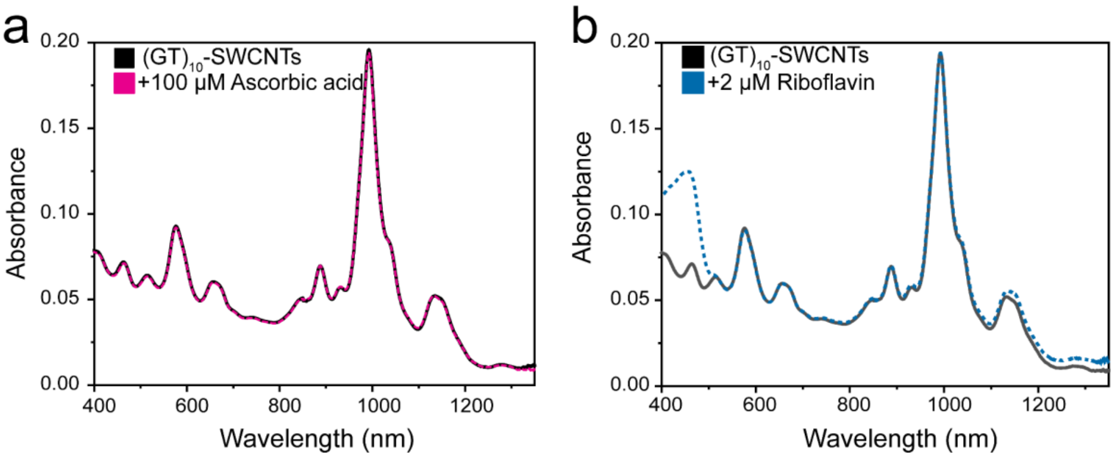

***Figure S13. Absorbance spectra of $(GT)_{10}$-SWCNTs in response to ascorbic acid and riboflavin**. The absorbance of $(GT)_{10}$-SWCNTs demonstrates no change when a) ascorbic acid (100 µM) or b) riboflavin (2 µM) is added. Note that riboflavin has a colour and therefore the spectrum in b with riboflavin contains an additional peak in the blue region. The relevant feature of the (6,5)-SWCNTs at around 1000 nm is not affected by the analyte, which shows that the analytes do not induce aggregation. They only change the fluorescence quantum yield (Figure 2a).*

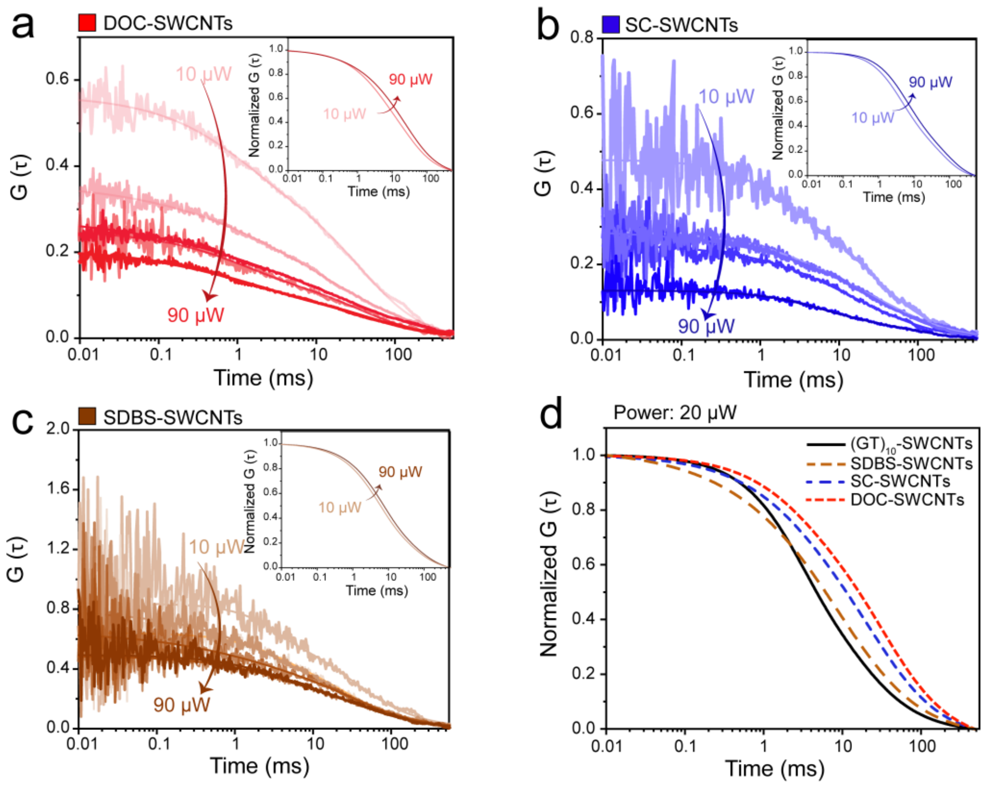

Figure S14. The organic corona can shield quantum friction. *Excitation power-dependent change (10 μW – 90 μW) of fluorescence autocorrelation functions of SWCNTs solubilized in different surfactants that create different organic coronas. The inset shows the normalized and fitted autocorrelation functions. See SI tables for fit functions. a) DOC-SWCNTs (red) b) SC-SWCNTs (blue) c) SDBS-SWCNTs (brown). d) Normalized autocorrelation functions of functionalized SWCNTs at 20 μW excitation power.*

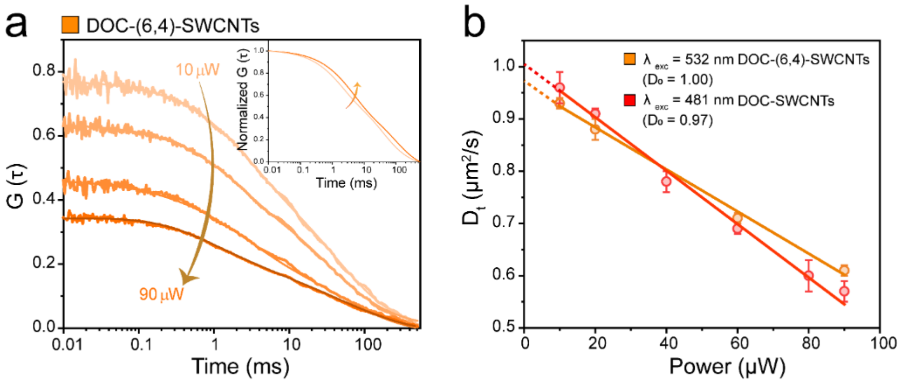

Figure S15. Comparison between DOC-(6,4)-SWCNTs and DOC-(6,5)-SWCNTs. *a) Fluorescence autocorrelation curves of DOC-(6,4)-SWCNTs (inset shows the normalized and fitted autocorrelation function) for an increasing excitation power. b) Diffusion constants of DOC-(6,4)-SWCNTs (orange) and DOC-SWCNTs (red, with mainly (6,5)-chirality) at different excitation power (n=3, mean ± SD). The fit is a linear equation and the dotted fit (black) represents the extrapolation of the diffusion constant (D_0) to zero excitation power.*

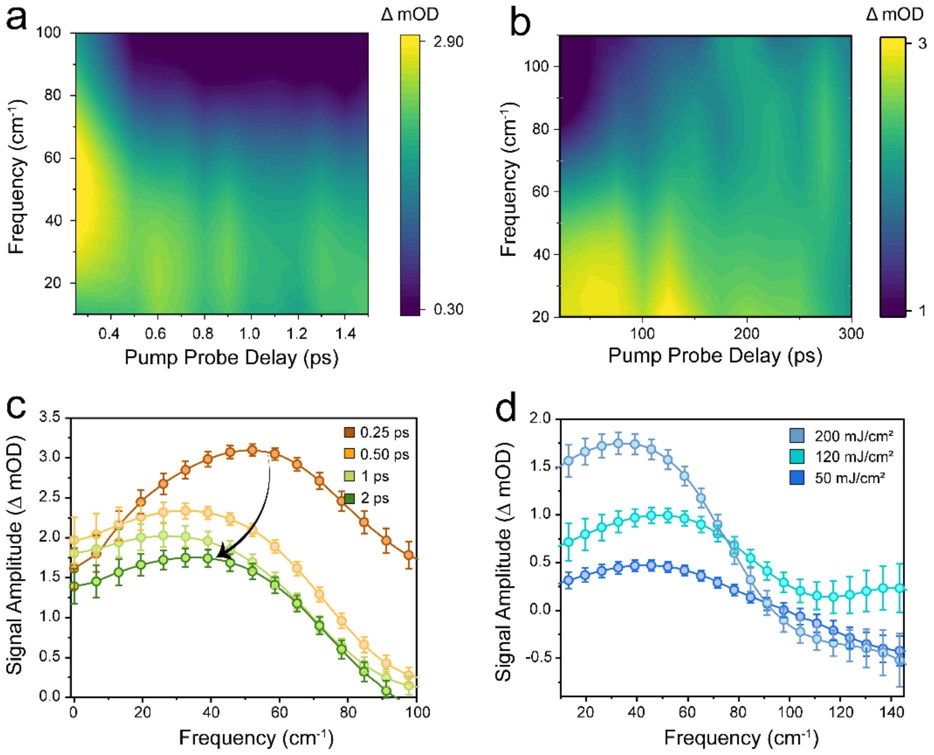

Figure S16. THz spectroscopy of DOC-SWCNTs in water: Temporal dynamics and fluence dependence. *a,b) The measured change in THz absorbance (Δ mOD) upon optical excitation versus without exciton excitation (400 nm) is plotted against pump-probe delays. c) Transient THz spectra (corresponding to vertical slices of the map at representative pump-probe time delays (0.25, 0.5, 1, and 2 ps). The arrow serves as a guide to the eye. d) Comparison of transient THz spectra at a pump probe delay of 2 ps for distinct fluences (200, 120, and 50 mJ/cm^2) (mean ± SE, n=22).*

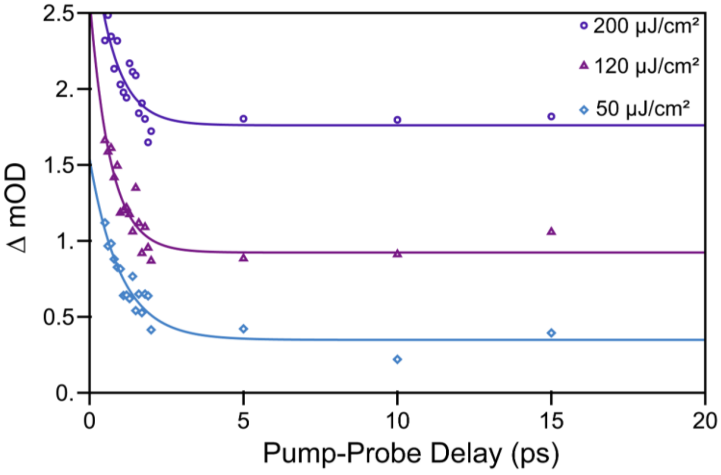

Figure S17. Pump–probe optical density changes: *Change of optical density of DOC-SWCNTs at a frequency of 26 cm^{-1} as a function of pump-probe delays. Plotted are the results for three distinct optical pump powers. The amplitudes were fitted (solid line) to the exponential function $\Delta mOD = c + A \exp(-t/\tau)$. The extracted decay times are τ = 1.0 ps for a flux of 50 $\mu J/cm^2$ and 0.7 ps for a flux of either 120 or 200 $\mu J/cm^2$.*

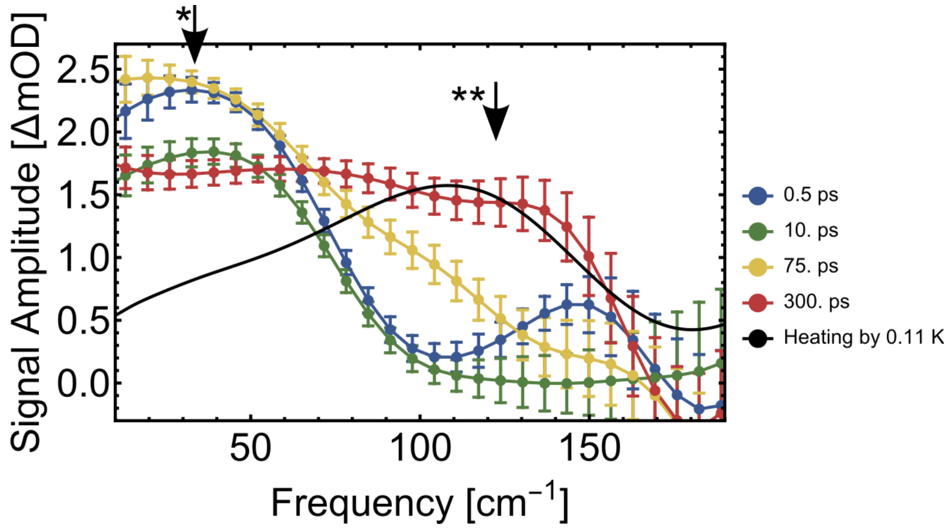

Figure S18. Optical Pump THz Probe (OPTP) spectroscopy of SWCNTs in water. *Plotted is the difference in THz absorption of an aqueous DOC-SWCNT solution as a function of time delay after optical excitation. One can compare the changes to the changes when heating a sample of bulk water e.g. by 0.11 K (from 10°C to 10.11°C, black line and modelled based on experimental steady-state spectra reported before2,3). The two arrows indicate the feature that we interpret as exciton-water coupling (*) and thermal heating (**) (mean ± SE, n=22).*

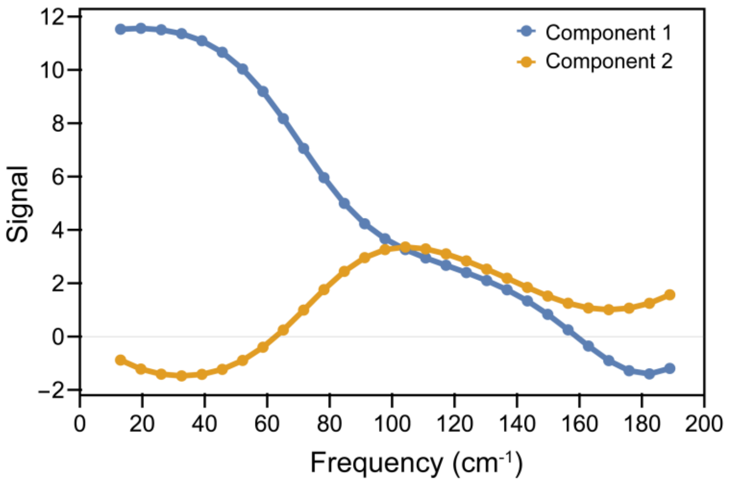

Figure S19. Singular value decomposition (SVD) of OPTP spectra of DOC-SWCNTs in water. *The first component (blue) has a maximum below 30 cm^{-1} (around 1 THz). The maximum of the second feature is centred at 100-120 cm^{-1}, resembling the spectrum of translational modes of the hydrogen bond network of water similar to heating of bulk water. SVD was performed (in Mathematica 14) with the spectra from 0.5 to 300 ps for a total of 31 time points from 13 cm^{-1} to 190 cm^{-1} (28 points each). The first two components represent 78% of the total weight of all components in the SVD.*

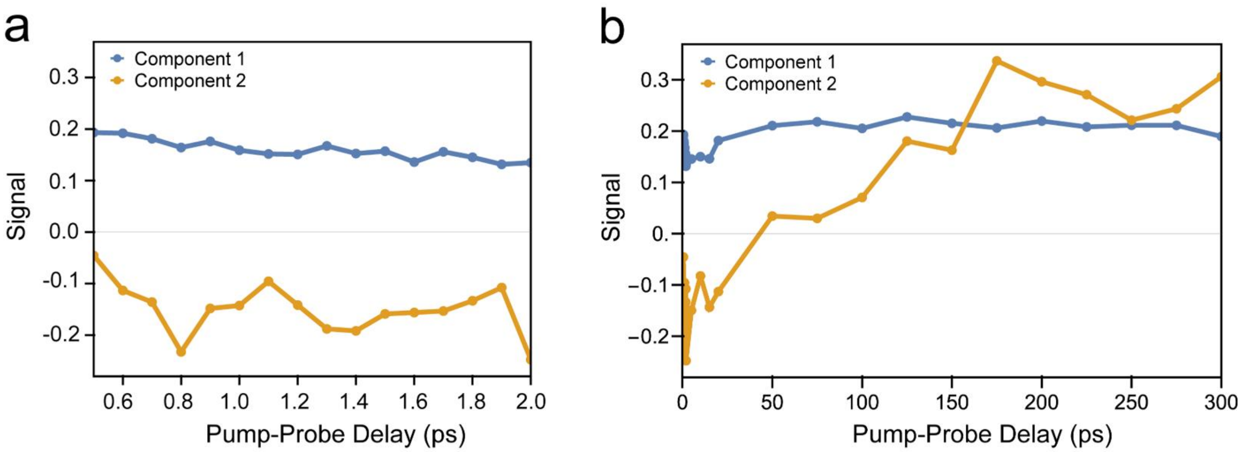

Figure S20. Temporal and spectral contributions of the two THz features. *Partial contribution of component 1 (blue) and component 2 (orange) from SVD as a function of time delay between the optical pump and the THz probe.*

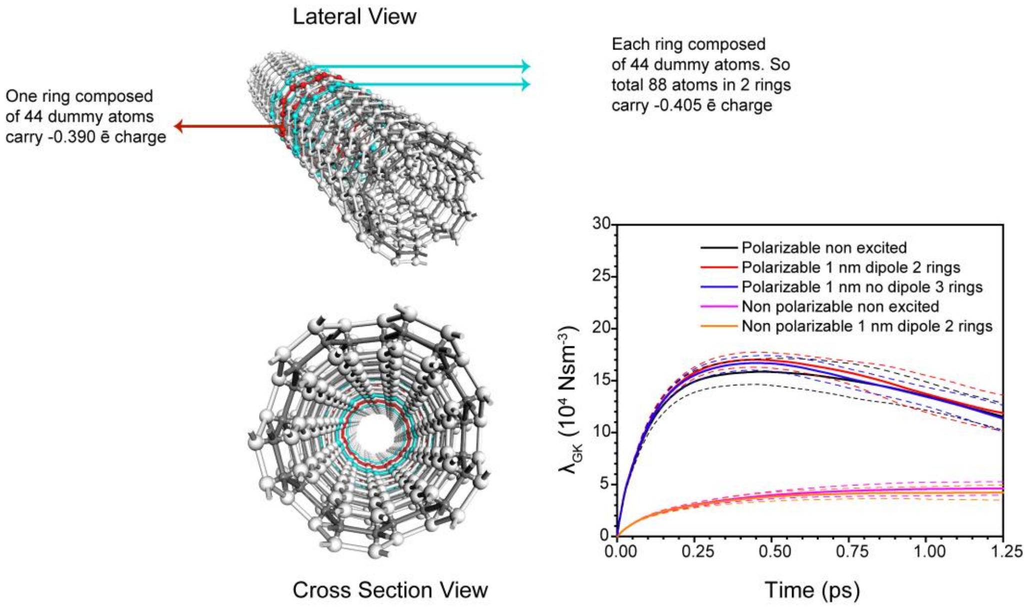

Figure S21. Friction Coefficient (calculated with the Green-Kubo formula) of dipole-free polarizable excitons*. Lateral and cross-sectional views of the SWCNT. Water is hidden for clarity. The two outer rings (blue) carry an additional -0.005 e charge on the 44 atoms in each ring, behaving as a delocalised electron. The delocalised hole is modelled by a central ring (red) composed of 44 atoms, each carrying an extra charge of +0.01 e. The distance between the centre and the outer rings was chosen to be around 0.5 nm. Note that the charge of the dummy atom is compensated by a +0.4 charge at each carbon atom. In this charge configuration, the net SWCNT dipole moment is zero compared to the configuration shown in Extended Data Figure 23, in which two rings describe the exciton. The dipole-free polarizable (3-ring) exciton (blue graph) and the normal (2-ring) polarizable exciton with net dipole (red graph) show similar friction coefficients. The uncertainty in the friction coefficient of dipole-free polarizable exciton is depicted by dotted blue lines and based on the standard deviation of 2000 replicas.*

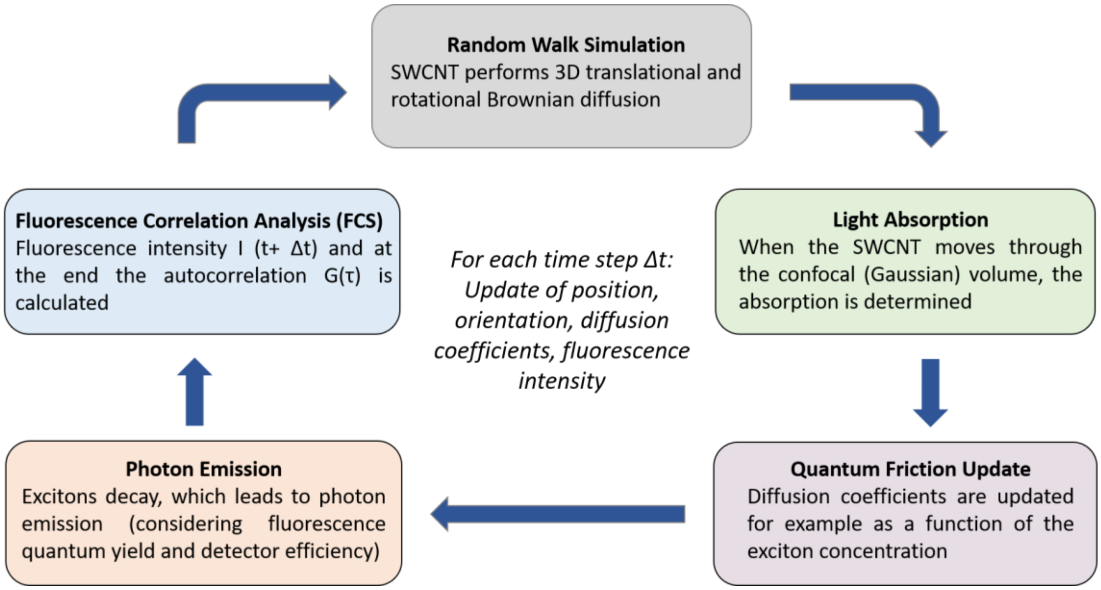

Figure S22. Schematic workflow of the SWCNT fluorescence correlation spectroscopy (FCS) simulation. *This schematic outlines the FCS simulation. It models the SWCNT's three-dimensional translational and rotational Brownian diffusion. As the SWCNT moves through the confocal Gaussian detection volume, the simulation calculates light absorption. The resulting exciton concentration can affect the quantum friction and thus diffusion constants. Photon emission is simulated according to exciton decay, fluorescence quantum yield, and detector efficiency. The resulting fluorescence intensity trace is analyzed to derive the autocorrelation function G(τ).*

FCS Simulation of SWCNTs

We performed Brownian dynamics simulations to model the anisotropic translational and rotational motion of a (6,5) SWCNT, diameter d = 0.78 nm, length L = 600 nm) dispersed in water under polarized optical excitation. The nanotube was treated as a rigid cylinder governed by slender-body hydrodynamics,[2] yielding direction-dependent translational and rotational diffusion coefficients at T = 298 K and water viscosity η = 0.89 mPa·s. The stochastic equations of motion were integrated using the Euler-Maruyama scheme in three dimensions with periodic boundary conditions for example in a 4 × 4 × 4 μm^3 cubic box to emulate bulk fluid behavior at the confocal scale.

Excitation incorporates orientation-dependent absorption via the $\cos^2\theta$ factor for linear polarization along the lab axis, with an effective cross-section σ_abs = 2.11 × 10^{18} m^2 at 480 nm (from NIR literature). Pulsed laser parameters (10 μW average power, 40 MHz repetition, ~0.25 pJ/pulse) yield a maximum exciton generation rate of 2.22 × 10^8 s^{-1} at the focus. To account for exciton-induced damping of SWCNT motion

(quantum friction via exciton-phonon drag), the translational and rotational diffusion coefficients were dynamically adjusted at each simulation step according to the instantaneous exciton generation rate The current exciton generation rate (R_{Power}) was calculated from the laser intensity within the confocal volume and normalized by the reference excitation rate (R_{ref}) corresponding to a laser power of 90 µW. We observed experimentally a decrease by around 50 % for the diffusion constant (figure 1), which is thus reproduced by this linear relation:

$$D_{eff} = D_0(1 - 0.5 \times \frac{R_{exciton}}{(R_{exciton,\ ref} \times 0.5)})$$

where D_0 is the diffusion coefficient in the absence of excitation-induced effects. Note that the second 0.5 factor is due to the (mean) orientation dependent excitation of SWCNTs when using polarized light. This very simplified approximation assumes a proportional decrease in diffusion with increasing exciton concentration and can be refined and other hypotheses tested in future work. For example, the different diffusion constants (translation, rotation, long axis, short axis) could be affected in different ways. The FCS simulation is provided as Python code in a folder inside the repository link.

Exciton number estimation

The exciton number estimation is provided as Python code in a folder inside the repository link.

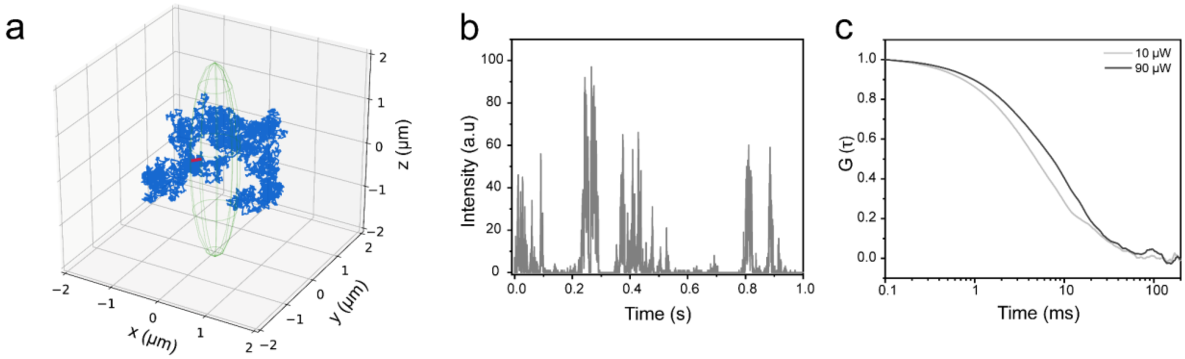

***Figure S23**. **Simulated diffusion of a SWCNT within the confocal volume.** a) Simulated diffusion of a 600 nm (6,5)-SWCNT through a confocal volume (effective volume: $\omega_0 \approx 0.48$ µm, $z_0 \approx 2.21$ µm; simulation box here: 4 × 4 × 4 µm³). a) Simulated trajectory (blue) of a SWCNT diffusing through the confocal volume (green wireframe). Orientation of the SWCNT at the last time point is shown in red. b) Corresponding fluorescence intensity trace. c) Autocorrelation functions for 10 µW (grey) and 90 µW (black) excitation power. The shift to the right (slower diffusion) fits qualitatively the experimental results (e.g., in Figure 1).*

FCS fit parameters

Table ST1. **$(GT)_{10}$-SWCNT FCS fitting parameters with increasing excitation power.** Off-state fraction *(T),* off state lifetime (τ_t) and stretching exponent *(β)* as obtained from fitting of PL correlation curves recorded at various excitation powers (mean ± SD, n=3).

Power (μW)	τ_d (ms)	D ($\mu m^2/s$)	τ_t (ms)	T	β	N	Brightness
10	14 ± 1.02	1.60 ± 0.07	2.0 ± 0.15	0.28 ± 0.008	0.80 ± 0.012	3 ± 0.31	1138 ± 400
20	16 ± 0.89	1.50 ± 0.04	2.0	0.28	0.72 ± 0.021	4 ± 0.61	1485± 310
40	18 ± 1.00	1.38 ± 0.07	2.0	0.28	0.61 ± 0.051	4.8 ± 0.49	2306 ± 256
60	20 ± 1.27	1.11 ± 0.04	2.0	0.28	0.52 ± 0.033	6.3 ± 0.25	3280 ± 166
80	22 ± 1.00	1.01 ± 0.09	2.0	0.28	0.48 ± 0.051	7.4 ± 0.32	4151 ± 298
90	24 ± 1.21	0.92 ± 0.06	2.0	0.28	0.47 ± 0.026	9.3 ± 0.21	4500 ± 300

Table ST2. Purified $(GT)_{10}$-SWCNTs (1 nM). *FCS fitting parameters with 10 and 90 μW power (mean ± SD, n=3).*

Power (μW)	τ_d (ms)	D ($\mu m^2/s$)	τ_t (ms)	T	β	Brightness	N
10	16 ± 1.0	1.5 ± 0.02	2 ± 0.6	0.3 ± 0.007	0.72 ± 0.002	3044 ± 200	1.6 ± 0.02
90	25 ± 1.1	1.0 ± 0.05	2	0.3	0.42 ± 0.008	7010 ± 301	4.0 ± 0.20

Table ST3. **$(GT)_{10}$-SWCNTs in D_2O (1 nM) FCS fitting parameters with increasing excitation power.** (mean ± SD, n=3)

Power (μW)	τ_d (ms)	D ($\mu m^2/s$)	τ_t (ms)	T	β	Brightness	N
10	30 ± 0.21	0.83 ± 0.008	1.0 ± 0.056	0.20 ± 0.006	0.60 ± 0.001	784 ± 11	1.5 ± 0.09
20	31 ± 0.49	0.81 ± 0.007	1.0	0.20	0.59 ± 0.002	869± 24	2.0 ± 0.23
40	30 ± 0.59	0.79 ± 0.01	1.0	0.20	0.58 ± 0.003	1033 ± 45	2.1 ± 0.12
60	31 ± 0.21	0.81 ± 0.01	1.0	0.20	0.58 ± 0.002	1195 ± 52	2.5 ± 0.14
80	31 ± 0.43	0.81 ± 0.005	1.0	0.20	0.57 ± 0.001	1378 ± 54	3.1 ± 0.12
90	31 ± 0.32	0.81 ± 0.006	1.0	0.20	0.57 ± 0.001	1474 ± 74	3.1 ± 0.24

Table ST4. **$(GT)_{10}$-SWCNTs in 80% glycerol (1 nM).** FCS fitting parameters with increasing excitation power (mean ± SD, n=3).

Power (μW)	τ_d (ms)	D ($\mu m^2/s$)	τ_t (ms)	T	β	Brightness	N
10	43 ± 1.4	0.66 ± 0.001	1 ± 1.2	0.10 ± 0.009	0.86 ± 0.008	400 ± 33	2.0 ± 0.09
90	43 ± 1.2	0.66 ± 0.002	1	0.10	0.82 ± 0.01	582 ± 31	3.0 ± 0.12

Table ST5. $(GT)_{10}$-SWCNTs in 20% glycerol (1 nM)*. FCS fitting parameters with increasing excitation power (mean ± SD, n=3).*

Power (μW)	τ_d (ms)	D ($\mu m^2/s$)	τ_t (ms)	T	β	Brightness	N
**10**	15 ± 1.20	1.67 ± 0.02	2.2 ± 0.12	0.30 ± 0.005	0.70 ± 0.010	1000 ± 200	4 ± 0.40
**40**	20 ± 1.00	1.25 ± 0.03	2.2	0.30	0.60 ± 0.012	1258 ± 160	6 ± 0.32
**80**	23 ± 1.10	1.09 ± 0.03	2.2	0.30	0.58 ± 0.024	3125 ± 193	6.3 ± 0.27

**90**	25 ± 1.00	1.00 ± 0.04	2.2	0.30	0.52 ± 0.029	4210 ± 138	7.1 ± 0.31

Table ST6. MSD fitting parameters. Diffusion constant D and power exponent n were obtained from a linear regression in log space.

**Sample**	**α**	**D ($\mu m^2/s$)**	**R^2**
DOC –SWCNT (200mW)	1.02	0.75	0.99
DOC-SWCNT (300 mW)	0.92	0.52	0.98

Table ST7. Concentration dependence of FCS data of 1 nM $(GT)_{10}$-SWCNTs exposed to riboflavin at 20 μW laser power.

**Riboflavin (μM)**	**τ_d (ms)**	**D ($\mu m^2/s$)**	**τ_t (ms)**	**T**	**β**	**N**	**Brightness**
0	16 ± 1.01	1.45 ± 0.04	2.0 ± 0.18	0.28 ± 0.008	0.72 ± 0.01	4 ± 0.98	1098 ± 70
0.1	15 ± 0.80	1.63 ± 0.08	2.0	0.28	0.70 ± 0.02	4.8 ± 0.78	1028 ± 52
0.5	12 ± 1.07	1.89 ± 0.09	2.0	0.28	0.68 ± 0.01	5.8 ± 0.32	872 ± 45
1	10 ± 0.50	2.37 ± 0.08	2.0	0.28	0.55 ± 0.01	6.5 ± 0.45	753 ± 38
2	8 ± 0.41	2.98 ± 0.14	2.0	0.28	0.45 ± 0.01	8.0 ± 0.38	510 ± 70

Table ST8. **Concentration dependence of FCS data of 1 nM $(GT)_{10}$-SWCNTs exposed to ascorbic acid at 20 μW laser power**.

**Ascorbic (μM)**	**τ_d (ms)**	**D ($\mu m^2/s$)**	**τ_t (ms)**	**T**	**β**	**N**	**Brightness**
0	16 ± 1.00	1.45 ± 0.04	2.0 ± 0.18	0.28 ± 0.008	0.72 ± 0.08	4 ± 0.12	1084 ± 213
5	18 ± 1.20	1.39 ± 0.05	2.0	0.28	0.73 ± 0.09	4.1 ± 82	1443 ± 243
20	20 ± 1.57	1.24 ± 0.03	2.0	0.28	0.78 ± 0.04	3.5 ± 0.42	2410 ± 312
50	24 ± 1.20	1.04 ± 0.06	2.0	0.28	0.82 ± 0.06	3.0 ± 21	3726 ± 501
100	37 ± 1.40	0.67 ± 0.04	2.0	0.28	0.88 ± 0.03	2.0 ± 0.82	6000 ± 458

Table ST9. DOC-SWCNTs (1 nM) FCS fitting parameters with increasing excitation power. (mean ± SD, n=3).

**Power (μW)**	**τ_d (ms)**	**D ($\mu m^2/s$)**	**τ_t (ms)**	**T**	**β**	**Brightness**	**N**
10	26 ± 1.0	0.96 ± 0.03	9.0 ± 0.21	0.50 ± 0.002	0.74 ± 0.01	1272 ± 100	1.8 ± 0.21
20	27 ± 2.1	0.91 ± 0.01	9.0	0.50	0.71 ± 0.02	2250 ± 190	2.7 ± 0.13
40	32 ± 1.3	0.78 ± 0.02	9.0	0.50	0.68 ± 0.01	2987 ± 195	2.1 ± 0.10
60	36 ± 2.5	0.69 ± 0.01	9.0	0.50	0.62 ± 0.02	4265 ± 100	3.2 ± 0.21
80	41 ± 2.8	0.60 ± 0.03	9.0	0.50	0.52 ± 0.01	4885 ± 134	4.2 ± 0.12
90	44 ± 2	0.57 ± 0.02	9.0	0.50	0.51 ± 0.01	5721 ± 145	5.0 ± 0.18

Table ST10. **DOC-SWCNTs with Nitro-Aryl sp^3 defect (1 nM) FCS fitting parameters with increasing excitation power**. (mean ± SD, n=3).

**Power (μW)**	**τ_d (ms)**	**D ($\mu m^2/s$)**	**τ_t (ms)**	**T**	**β**	**Brightness**	**N**

10	19 ± 1.1	1.32 ± 0.01	4.0 ± 0.12	0.30 ± 0.01	0.80 ± 0.01	300 ± 10	2.3 ± 0.42
20	19 ± 1.1	1.31 ± 0.02	4.0	0.30	0.80 ± 0.01	312 ± 10	2.5 ± 0.21
40	19.5 ± 1.0	1.28 ± 0.01	4.0	0.30	0.78 ± 0.02	331 ± 9	3.0 ± 0.12
60	20 ± 1.2	1.25 ± 0.02	4.0	0.30	0.78 ± 0.01	368 ± 23	3.1 ± 0.21
80	20 ± 1.0	1.22 ± 0.01	4.0	0.30	0.78 ± 0.02	412 ± 17	4.0 ± 0.24
90	20.5 ± 1.1	1.22 ± 0.01	4.0	0.30	0.78 ± 0.01	425 ± 19	4.5 ± 0.14

Table ST11. SC-SWCNTs (1 nM) FCS fitting parameters with increasing excitation power. (mean ± SD, n=3).

**Power (μW)**	τ_d **(ms)**	**D (μm²/s)**	τ_t **(ms)**	**T**	**β**	**Brightness**	**N**
10	24 ± 1.20	1.00 ± 0.02	2.0 ± 0.04	0.60 ± 0.007	0.68 ± 0.02	1228 ± 100	2.5 ± 0.18
20	26 ± 1.40	0.96 ± 0.01	2.0	0.60	0.64 ± 0.02	1460 ± 98	3.1 ± 0.19
40	28 ± 2.30	0.89 ± 0.03	2.0	0.60	0.60 ± 0.01	1880 ± 143	4.0 ± 0.16
60	30 ± 1.40	0.83 ± 0.01	2.0	0.60	0.60 ± 0.01	2071 ± 120	5.0 ± 0.27
80	32 ± 1.60	0.78± 0.02	2.0	0.60	0.58 ± 0.02	2463 ± 131	5.6 ± 0.22
90	34 ± 1.20	0.73± 0.01	2.0	0.60	0.54 ± 0.01	2635 ± 140	6.0 ± 0.32

Table ST12. SDBS-SWCNTs with sp³ defect (1 nM) FCS fitting parameters with increasing excitation power. (mean ± SD, n=3).

**Power (μW)**	τ_d **(ms)**	**D (μm²/s)**	τ_t **(ms)**	**T**	**β**	**Brightness**	**N**
10	18 ± 0.30	1.30 ± 0.02	2.3 ± 0.08	0.60 ± 0.002	0.86 ± 0.01	546 ± 23	3.0 ± 0.12
20	20 ± 0.50	1.24 ± 0.01	2.3	0.60	0.84 ± 0.01	581 ± 12	3.8 ± 0.19
40	21 ± 0.21	1.16 ± 0.01	2.3	0.60	0.80 ± 0.02	683 ± 33	4.0 ± 0.10
60	23 ± 0.42	1.06 ± 0.01	2.3	0.60	0.78 ± 0.01	803 ± 24	5.7 ± 0.09
80	25 ± 0.45	1.02 ± 0.02	2.3	0.60	0.78 ± 0.01	902 ± 32	5.8 ± 0.10
90	26 ± 0.34	1.00 ± 0.01	2.3	0.60	0.76 ± 0.01	956 ± 21	6.0 ± 0.10

Table ST13. **DOC-(6,4)-SWCNTs (1 nM) with power**. FCS fitting parameters with increasing excitation power (mean ± SD, n=3).

**Power (μW)**	τ_d **(ms)**	**D (μm²/s)**	τ_t **(ms)**	**T**	**β**	**Brightness**	**N**
10	27 ± 0.7	0.93 ± 0.01	2.5 ± 0.12	0.70 ± 0.02	0.77 ± 0.01	3533 ± 121	1.0 ± 0.10
20	28 ± 0.8	0.88 ± 0.02	2.5	0.70	0.77 ± 0.008	4221 ± 187	2.0 ± 0.13
60	35 ± 1.0	0.71 ± 0.01	2.5	0.70	0.72 ± 0.01	6550 ± 196	2.3 ± 0.12
90	41 ± 0.9	0.61 ± 0.01	2.5	0.70	0.69 ± 0.01	9122 ± 132	3.5 ± 0.21

Table ST14. Comparison of pulsed and continuous laser excitation in (GT)$_{10}$-SWCNT: *FCS fitting parameters with 10 and 90 μW power (mean ± SD, n=3).*

**Power (μW)**	τ_d **(ms)**	**D (μm²/s)**	τ_t **(ms)**	**T**	**β**	**N**	**Brightness**

**Pulsed**							
10	14 ± 1.02	1.60 ± 0.07	2.0 ± 0.15	0.28 ± 0.008	0.80 ± 0.012	3 ± 0.31	1138 ± 400
90	24 ± 1.21	0.92 ± 0.06	2.0	0.28	0.47 ± 0.026	9.3 ± 0.21	4500 ± 300
**Continuous**							
10	14 ± 0.08	1.60 ± 0.023	2.0 ± 0.078	0.31 ± 0.021	0.70 ± 0.002	2.0 ± 0.10	2015 ± 200
90	25 ± 0.98	0.90 ± 0.03	2.0	0.31	0.53 ± 0.013	7.0 ± 0.18	6290 ± 398

***Table ST15**. **(GT)$_{10}$-SWCNTs with low and medium defect densities (1 nM).** FCS fitting parameters with 10 and 90 µW power (mean ± SD, n=3).*

Power (µW)	τ_d (ms)	D (µm^2/s)	τ_t (ms)	T	β	Brightness	N
Low-defect 10	23 ± 1.3	1.09 ± 0.01	3 ± 0.8	0.30 ± 0.006	0.80 ± 0.004	2773 ± 93	2.0 ± 0.05
Low-defect 90	32 ± 2.1	0.78 ± 0.001	3	0.30	0.67 ± 0.008	8578 ± 61	5.0 ± 0.10
Medium-defect 10	21 ± 1.0	1.19 ± 0.012	3.7 ± 0.8	0.50 ± 0.004	0.72 ± 0.002	1598 ± 94	1.9 ± 0.04
Medium-defect 90	26 ± 0.8	0.96 ± 0.001	3.7	0.50	0.69 ± 0.009	2967 ± 121	3.6 ± 0.20
High-defect 10	22 ± 1.0	1.00 ± 0.003	3.2 ± 0.5	0.60 ± 0.002	0.70 ± 0.003	1238 ± 67	2.2 ± 0.03
High-defect 90	22 ± 0.5	1.00 ± 0.002	3.2	0.60	0.65 ± 0.005	1345 ± 101	4.0 ± 0.32

Table ST16. Linear fit quality for the various functionalized SWCNTs for the power dependent diffusion constant.

Surface modification	R^2
(GT)$_{10}$-SWCNTs	0.996
SDBS-SWCNTs	0.967
SC-SWCNTs	0.997
DOC-SWCNTs	0.990
DOC-(6,4)-SWCNTs	0.995

Table ST17. Polar and Non-polar model parameters. Table of parameters for classical MD of a polarizable and nonpolar CNT and flexible SCPE water model along with equation for total energy (Etotal) comprising kinetic (KE) and potential energy (PE) terms. All the models are also included as part of the supporting files.

$E_{tot}=E_{KE}+E_{PE}$ where $E_{PE}=E_{Bond}+E_{Angle}+E_{dihedral}+E_{Improper}+E_{vdw}+E_{Coulomb}$<br>$E_{Bond}=\mathbf{k}(r_{ij}-\mathbf{r_0})^2$; $E_{Angle}=\mathbf{k}(\Theta_{ijk}-\mathbf{\Theta_0})^2$; $E_{dihedral}=\mathbf{k}[1+\mathbf{d}\cos(\mathbf{n}\Phi_{ijkl})]$; $E_{Improper}=\mathbf{k}[1+\mathbf{d}\cos(\mathbf{n}\Phi_{ijkl})]$<br>$E_{Coulomb}=k\mathbf{q_i}\mathbf{q_j}/r_{ij}^2$; $E_{vdw}=\boldsymbol{\varepsilon}[(\boldsymbol{\sigma_0}/r_{ij})^{12}-2(\boldsymbol{\sigma_0}/r_{ij})^6]$			
**IFF-CVFF Polarizable Graphene/CNT Parameters**			
Mass (amu)[1]	C 10.011150	D 1.000000	
Bond<br>k: kcal/mol, r_0: Å	C-C<br>(k=480, r_0=1.558)	C-D<br>(k=150, r_0=0.60)	D-D[2]<br>(k=0, r_0=1.558)
Angle<br>k: kcal/mol, Θ_0: deg.	C-C-C<br>(k=60, Θ_0=120)	C-C-D<br>(k=25, Θ_0=90)	D-C-D<br>(k=25, Θ_0=180)
Dihedral<br>k: kcal/mol, r0: Å	C-C-C-C<br>(k=24, d= -1, n= 2)		
Non-Bond[3]<br>q:e, σ_0:Å, ε:kcal/mol	C<br>(q = 0.8, σ = 4.167, ε = 0.025)	D<br>(q = -0.4, σ = 1.800, ε = 0.045)	
**CVFF Non-polar Graphene/CNT Parameters**			
Mass (amu)[1]	C 12.011150		
Bond (k: kcal/mol, r_0: Å)	C-C (k=480, r_0=1.340)		
Angle (k: kcal/mol, Θ_0: deg.)	C-C-C (k=90, Θ_0=120)		
Dihedral (k: kcal/mol)	C-C-C-C (k=3, d= -1, n= 2)		
Improper (k: kcal/mol)	C-C-C-C (k=0.37, d= -1, n= 2)		
Non-Bond[3] (q:e, σ_0:Å, ε:kcal/mol)	C (q = 0.0, σ = 4.060, ε = 0.148)		
**Flexible SPC Water Parameters**			
Mass (amu)[1]	O 15.999400	H 1.007970	
Bond (k: kcal/mol, r_0: Å)	O-H (k=540.634, r_0=0.960)		
Angle (k: kcal/mol, Θ_0: deg.)	H-O-H (k=50, Θ_0=104.5)		
Non-Bond[3]<br>(q: e, σ_0: Å, ε: kcal/mol)	O<br>(q=-0.82, σ=3.553, ε=0.155)	H<br>(q=+0.41, σ=0.242, ε=0.025)	

[1]C=Carbon, D=Dummy atom, O=Oxygen, and H=Hydrogen, [2]Bonded interactions are added to the dummy atoms in the polarizable model to avoid calculation of 1-4 interactions to increase the flexibility of the dummy atoms in the polarizable graphite/CNT model even though some of the bonds and angles constants are 0. [3]Geometric mixing rule is applied to evaluate the ij Lennard Jonesd interaction parameters from pure component ii and jj interactions.

References

1. Charsooghi, M. A., Akhlaghi, E. A., Tavaddod, S. & Khalesifard, H. R. A MATLAB program to calculate translational and rotational diffusion coefficients of a single particle. *Comput. Phys. Commun.* **182**, 400–408 (2011).

2. Aragon, S. R. & Flamik, D. High precision transport properties of cylinders by the boundary element method. *Macromolecules* **42**, 6290–6299 (2009).
