# Supplementary material for: Light-induced quantum friction of carbon nanotubes in water
Source: Nature. 2026 Jun 10;654(8120):941–7. doi: 10.1038/s41586-026-10632-2 (PMC13293881; doi:10.1038/s41586-026-10632-2)
Supplement: Supplementary file 2 — Peer Review File [file 41586_2026_10632_MOESM2_ESM.pdf]

nature portfolio

Peer Review File

Light-induced quantum friction of carbon nanotubes in water

Corresponding Author: Professor Sebastian Kruss

Any redactions in this file are there to maintain patient confidentiality, the confidentiality of unpublished data, or to remove third-party material.

This file contains all reviewer reports in order by version, followed by all author rebuttals in order by version.

Version 0:

Reviewer comments:

Referee #1

(Remarks to the Author)
This paper studies the diffusivity of single-walled carbon nanotubes in water, using mainly fluorescence correlation spectroscopy (FCS). The diffusivity is observed to depend on the FCS laser intensity. The authors establish that the main effect of the laser intensity is to modulate the nanotube exciton concentration in the steady state, and they argue that it is a coupling between exciton dynamics and water charge fluctuations – according to a quantum friction mechanism – that is responsible for the changes in diffusivity. They further carry out molecular dynamics simulations showing that an additional dipole moment, as would be introduced by an exciton, indeed significantly slows down the diffusion of a CNT in water.

The conclusion that CNT diffusion in water can be modulated by pumping them into an electronically excited state is a very significant one, given the growing interest in controlling nanoscale fluid transport using the confining wall's electronic properties. However, the authors go even further and conclude that their "study demonstrates that quantum friction between SWCNTs and water exists". I think that the reported data is insufficient to serve as a proof of water-CNT quantum friction. I recommend that the authors revise their manuscript to draw only the conclusions that can be supported by the data.

The authors seem to be unclear on the definition of quantum friction. The abstract begins with "quantum friction describes the transfer of energy and momentum from electronically excited states in a material to a surrounding solvent". As introduced in Ref. 2 of the manuscript, quantum friction rather refers to a direct transfer of momentum between a liquid and a solid's electrons. Importantly, the solid does not need to be put in an electronically excited state for there to be quantum friction: it is rather the interaction between thermal fluctuations of the solid and the liquid that mediates the momentum transfer. If demonstrated, quantum friction involving a solid in an excited steady state (here, CNT with an exciton population) would be a new and interesting phenomenon. Now, the conclusion states "our study experimentally demonstrates that quantum friction i.e. light-induced slowing down of Brownian motion exists". This is inconsistent with the introductory statements and the cited references.

Furthermore, quantum friction relies on the interaction between charge fluctuations. The excitons in the CNTs have a static dipole moment, which could be responsible for an increase in friction, consistently with the reported MD simulations – this would then not be quantum friction, but rather dielectric friction, as first introduced in [R. Zwanzig, J. Chem. Phys. 38, 1603 (1963)]. Quantum friction would arise from an interaction between the charge fluctuations due to exciton diffusion along the CNT with the water charge fluctuations. Such a mechanism is supported by the experiments with defected CNTs, but MD simulations suggest a significant effects of a static exciton. Based on the reported data, I think it is difficult to draw a firm conclusion regarding the relative importance of dielectric and quantum friction.

Now, for the conclusion that the exciton population in CNTs modulates their diffusivity to be unambiguous, I suggest the authors clarify several points:
1. What is the purity of the samples in terms of nanotube chiral indices? Can artifacts due to the presence of metallic nanotubes in the sample by excluded?
2. FCS monitors nanotubes as they enter and exit a confocal volume. But the exciton population in a nanotube is maintained in a steady state only when the nanotube is inside the confocal volume. Thus, the diffusivity measurement and the dynamics of the exciton population are entangled. I believe that a measurement where this is not the case is required for the conclusion to be unambiguous. The authors do carry out a macroscopic viscosity measurement, but only with a (putative)

chemical modulation of exciton population. Could the authors monitor the viscosity (and temperature) of the CNT solution under varying 485 nm light intensity? More generally, a diffusivity measurement with no light intensity gradient would help make the conclusion more robust.
3. What is the role of laser pulsing in the FCS measurements?
4. MD simulations: even after a detailed reading of the methods and supplementary information, it is not clear to me how the exciton is modeled. What is the total charge of the electron and hole? Is the polarizable force field necessary to draw the qualitative conclusion? From the experimental side, what is the expected exciton concentration in the steady state as a function of light intensity, and how does it compare to the simulation?

A minor terminology point: in the introduction “simulations offer insights into the anomalously high water friction on graphite” – this insight rather comes from analytical theory.

In summary, I think that the authors report some very interesting data that suggest a coupling between CNT excitons and water dynamics. However, it is not a proof of water-CNT quantum friction, and I strongly suggest that the authors tone down the wording in this regard, in addition to addressing the above points.

Referee #2

(Remarks to the Author)
The manuscript by Havenith, Sulpizi, Kruss and co-workers describes a series of deceptively simple experiments that observe a puzzling decrease of the diffusion constant of functionalized carbon nanotubes in water. The FCS experiments observe that under optical excitation the CNT particle slow down by up to a factor of two, which is a significant effect.

In two key experiments, the observed diffusion coefficient decrease vanishes when the CNTs are functionalized with defects that suppress exciton diffusion and also when water is replaced by heavy water (D2O). These experiments (especially the D2O one) point out to interesting fundamental physics that could be contributing to these results. However, before I become fully convinced, I would like to point out to some additional questions and comments:

1. I think more characterization of CNTs used in the study is in order. What was the length distribution of the CNTs and how uniform were those tubes. Of course expecting a uniform distribution is not very realistic, but some idea of the length distribution (for example from HR-TEM studies) would be very helpful, as different CNT lengths may affect the exciton dynamics.
2. The manuscript talks about different density from the various surfactants used to wrap the CNTs. Are there any data supporting these assessments?
3. (6,5) and (6,4) CNTs are both semiconducting species, so the results presented in the manuscript that argue that CNT chirality does not affect the observed effects are not such a great test. Using a metallic CNT sample would have been much more revealing.
4. Why was Figure S7b normalized to the diffusion constant at 10uW power? Should not it be normalized to D_0 (i.e. extrapolated value at zero power)?
5. The results on chemical modification of the observed effects feel less impressive. Did the author check that ascorbic acid and riboflavin did not change association/dissociation of CNTs? For example, what would dynamic light scattering measurements show?
6. On the same note, small compounds can affect hydrogen bonding in water (i.e. exhibit chaotropic or kosmotropic behavior). Those changes can plausibly affect the diffusion dynamics significantly even in absence of any quantum friction effects.
7. The most puzzling component of the manuscript is PS bead experiment, which frankly feels like it throws some sand in the otherwise interesting and well-constructed story. Does this experiment mean that all microscale object exhibit quantum friction? Do the PS beads exhibit the same exciton dynamics? If so, why? What about the drastic differences in size, geometry, or materials properties between CNTs and PS beads? What is the actual mechanism of ascorbic acid effect on the mobility of a relatively large particle?
8. I cannot help, but feel that more insights could be gained from MD simulations, which feel almost like a missed opportunity in the way the results are presented. The MD simulations section is painfully short and misses all the possible atomistic level insights that could potentially be gained from these studies.

Finally, some additional comments on the manuscript structure. First the introduction feels meandering. It talks about using CNTs for biosensing, which is highly irrelevant to the topic of the manuscript. I suggest streamlining it. One the same token, the topic of quantum friction, which is central to this work, is not addressed in any depth. “Energy and momentum transfer to a surrounding solvent” can easily describe standard non-radiative relaxation, so a description that pays more attention to the physics of quantum friction will be very welcome. Finally, as I mentioned before, MD simulations are described in a very cursory way, even though they provide an important validation and perhaps the only way to see what is happening in this system at the atomic level.

Referee #3

(Remarks to the Author)
The manuscript titled "Light-induced quantum friction of carbon nanotubes in water" by Kruss et al. reports on deacceleration of diffusive motion of single-walled carbon nanotubes (SWCNTs) in water by light irradiation. The authors conducted fluorescence correlation spectroscopy measurements on SWCNTs with various laser powers, surfactants, and solvents to

show that coupling of excitons in SWCNTs to surrounding water molecules generates friction, i.e., light-induced quantum friction. They carried out optical-pump THz-probe measurements to investigate excitation of collective translational modes of water molecules induced by light irradiation of SWCNTs. Molecular dynamics simulations were performed to support the decrease in diffusion constant by coupling of exciton to water molecules. The authors demonstrated the visualization of diffusive motion of beads with attached fluorescent dye molecules in the SWCNT aqueous dispersion.

All the experiments were well done, and the details of methods were sufficiently described in the text. The data and results were well presented with appropriate descriptions of errors except for Figures S7 and S9. The abstract, introduction and conclusions are clear and appropriate, and the conclusions are valid.

This paper provides an interesting concept of quantum friction on one-dimensional materials induced by exciton-water coupling, which is the outstanding feature of the work and should be of significance and broad interest in the field of physics and chemistry on nanomaterials. The quantum friction by plasmon-water coupling for carbon materials was reported (refs. 2-5). The plasmon is a collective excitation, while the exciton is an excited state consisting a single electron and hole. The concept of exciton-water coupling induced friction is new and should be immediately recognized, examined and used by the researchers in the field. Thus, I think the paper potentially deserves publication. However, before publication, I recommend the revision concerning the following points to strengthen the paper.

1. Page 5
In the second paragraph, the authors rejected the possibility of sample heating by a control experiment using the dye Atto 488. Is the fluorescence quantum yield of this dye similar to that of SWCNT? If the quantum efficiency of the dye is much higher than that of SWCNT, the laser-induced heating of dye molecules and surrounding water molecules is low. (The relaxation by fluorescence does not rise temperature.)

2. Page 7
I guess that the Coulomb interaction between a dipole of water molecule and polarization of trapped/locaized exciton on diffusive (moving) SWCNT could affect friction. It is helpful to make a physical explanation why the trapped/localized exciton does not affect friction. (This comment is related to the comment 5 below.)

3. Page 7
The dielectric constant of surfactants could affect the interaction between exciton and water molecule (screening effect). Is it possible to discuss the screen effect of surfactants used in this study?

4. Page 9
In the optical-pump THz-probe measurements (Figures 3c and 3d), the transient signal lasts at least 300 ps. Were the excitons long-lived in this time scale? It is helpful to show fluorescence lifetime of excitons.

5. Page 11
The Drude-like oscillators are suitable for describing classical plasmons, but not for an electron and hole consisting of exciton, because the exciton is a bound state of them. Which modes (excitations) in exciton are used to couple the collective translational modes of water molecules?

Version 1:

Reviewer comments:

Referee #1

(Remarks to the Author)
The authors made significant revisions to the manuscript. In particular, they have carried out additional single nanotube tracking experiments that support their conclusions. However, in many places, the physical interpretations are still very fuzzy. Particularly, when it comes to interpreting OPTP measurements and MD simulations, it looks like conceptual misunderstandings lead the authors to draw conclusions that are not supported by the data.

The main issue is summed up in the very first sentence of the abstract, which has barely changed since the last version, although I explicitly mentioned it in my previous report. The authors state: “Quantum friction describes direct transfer of energy and momentum between electronic states in a material and a surrounding solvent”. Quantum friction refers specifically to the transfer of momentum between a liquid and a solid’s electrons. This momentum transfer is not necessarily accompanied by a net energy transfer. A similar microscopic mechanism is involved in the direct energy transfer between an electronically excited solid and a surrounding liquid, which is referred to as near-field radiative heat transfer (NFRHT). However, the observation of NFRHT does not imply that quantum friction is the dominant mechanism for solid-liquid momentum transfer, nor does the observation of quantum friction imply that NFRHT is the dominant mechanism for solid-liquid heat transfer. Nevertheless, the authors use these two concepts interchangeably. For instance, they conclude: “On the 0.7 ps time scale a rapid response is observed. Similar to (hot electron cooling in) graphene, we attribute this fast decay to quantum friction between SWCNTs and water”. If the decay can be attributed to anything it is to NFRHT, not quantum friction! Even if the spectra represent indeed the energy transfer pathways suggested by the authors, this does not support the interpretation of the diffusion slowdown in terms of quantum friction, unless a quantitative analysis of the timescales is made as in Ref. 7. At present, I feel that the THz measurements can be an interesting side note, but they cannot support the

overall argument.

Further confusion is generated by the implied equivalence between diffusion and friction. In its original formulation, quantum friction refers to a contribution to the friction force experienced by a liquid when flowing along a solid surface or through a nanotube. Here, the authors measure the diffusion coefficient of nanotubes in water, which is in principle linked to the water-nanotube friction coefficient through the Stokes-Einstein relation, but the link is far from obvious. This link should be made explicit in order to attribute the observed changes in diffusion to quantum friction.

The are more issues in the analysis of the MD simulations. i) On the technical side: the authors use charges of 0.2e for the model electron and hole because the simulation is unstable for higher charges, but this information is buried in the methods section. This is an important piece of information that should be in the main text. ii) The authors imply that the correlation between the CNT dipole moment and the overall dipole moment of the water in the simulation box is somehow related to water-CNT momentum transfer. It is quite unclear to me why this would be the case. In fact, the polarizable force field allows the CNT dipole moment to almost instantaneously adapt to the water configuration - it is then obvious that the water and CNT dipole moments should be more correlated than in the non-polarizable case, without any implications for CNT diffusion. iii) The authors state "These results further confirm that an electron-hole (dipole) is not a sufficient condition to enhance the friction. The key element to couple charge dynamics to water dynamics is the dynamic nature of the exciton in SWCNTs (polarizability), resulting in enhanced friction". This statement seems to draw an equivalence between exciton dynamics due to diffusion and exciton polarizability. A key experimental result is that the light-induced diffusion modulation is suppressed by defects in the CNT, which are assumed to pin the excitons. The simulations might show slower CNT diffusion when the model exciton is polarizable, but they do not explain why a pinned exciton would have no effect on CNT diffusion. Results for the MSD with a non-polarizable force field are not shown, despite the question in my previous report. Could the role of defects be more complex than exciton pinning? Overall, I urge the authors to acknowledge uncertainties in interpretation rather than to sweep them under the rug. iv) The analysis of the phonon effect in the framework of the MD simulation makes no sense to me as it is based, again, on the water-CNT dipole correlation. v) Given all the above, "the MD simulations validated the experimental findings" is a gross overstatement. I urge the authors to be more careful in distinguishing fact from guesswork.

Finally, I think that the overall presentation and clarity are not on the level that can be expected from a Nature paper. The introduction still feels "meandering" – in the words of Reviewer 2 – despite the revision. The figures could be much better organized. For instance, the single CNT tracking is an important result that could be in Figure 1, and not in Figure 4 which is mainly about simulations. Some of the data in the supplementary figures would be welcome in the main figures (e.g., the single CNT tracking trajectories; note the label issue in Fig. S29). The main figures could be a lot more information-dense to avoid a constant back-and-forth between main paper and SI. The conclusion makes some statements with no logical sense, e.g. "These findings show that exciton-mediated quantum friction directly affects the motion of a nanoscale object and that there is momentum transfer". What are the entities involved in the momentum transfer? The conclusion feels overall very weak and misses the fundamental implications of the findings for nanofluidic transport.

Referee #2

(Remarks to the Author)
The authors have done a very impressive work addressing my comments. The new data on trajectory tracking, as well as the additional modeling efforts strengthen the paper and make the argument that it presents more compelling. I also appreciate including more discussion of quantum friction that places es the results in context. I would support publication of this work at this stage.

Referee #3

(Remarks to the Author)
The manuscript has been significantly improved. It provides an interesting concept of quantum friction on one-dimensional materials induced by exciton-water coupling, as I commented in the previous report. I think this manuscript now deserves publication.

Version 2:

Reviewer comments:

Referee #1

(Remarks to the Author)
The authors have carried out substantial revision work, including additional experiments and data analysis, largely addressing the points raised in my previous report. In particular, I am happy to see that the revised manuscript is more careful in drawing conclusions, ensuring that these are better supported by the data. Below are the few points that I still find somewhat unclear or inaccurate.

1) In the Introduction "Optical pump Terahertz (THz) probe experiments showed very short time scales of cooling via quantum friction in water, which was attributed to the coupling of graphene surface plasmon modes and water charge fluctuations in the frequency range of the librational mode of water in the THz region" – there is still a confusion between

energy transfer and quantum friction. In Ref. 7, the very fast electron cooling could be attributed to direct energy transfer (near-field radiative heat transfer) between the graphene electrons and water, which is not quantum friction. Indeed, by design, OPTP can only probe energy transfer, and not momentum transfer. But because OPTP probes energy transfer between the degrees of freedom that are potentially involved in quantum friction, it can provide indirect indication of the latter.

2) The discussion of the OPTP experiments has become significantly clearer and I now agree that they qualitatively support the light-induced quantum friction mechanism put forward by the authors. But there is still a conceptual confusion, because quantum friction and NFRHT are presented as two distinct mechanisms that correspond to two different features in OPTP spectra: "Therefore, we propose that the 30 cm-1 feature provides the pathway to exchange momentum via quantum friction. Near field radiative heat transfer (NFRHT) is another possible mechanism for the exciton to exchange energy with water but would rather contribute to the second (thermal) component (>100 cm-1)". However, I insist that the only thing that OPTP measurements can reveal is NFRHT between electrons and water. I think that the reported measurements are likely indicating exactly that, and this comes in qualitative support of the importance of quantum friction in the CNT diffusion. Indeed, as discussed in Ref. 7, the usual ("classical") pathway of electron-liquid energy transfer goes through phonons, and I expect that the energy transfer from thermalized phonons to water should indeed look like water heating on 10-100 ps timescales. The faster dynamics in the low-frequency part of the spectrum could thus indeed correspond to direct energy transfer (NFRHT) between the excitons and water. NFRHT is not "just heating": it can occur between non-thermalized degrees of freedom. I strongly suggest rephrasing the statement quoted above.

3) I am still puzzled by the cross-correlation analysis of the exciton dipole and total water dipole in the MD simulations. The response letter states "The dipole-dipole correlation is only one part of the analysis and was performed to find out if there is spectral overlap. It is indeed related to the question whether energy transfer is possible in the system". I assume that the authors refer to spectral overlap between the water and the dipole's charge fluctuations. If so, then I am still convinced that this cross-correlation analysis brings no additional information regarding the possibility of energy transfer or quantum friction between CNT excitons and water. Indeed, the polarizable force field used by the authors is such that the dummy charges adapt almost instantaneously to the water configuration: the dummy charges are much faster than water, so there is no overlap between the bare charge fluctuations spectra. This is different from the setup of Bui et al, where the Drude charges evolve on timescales that are similar to water timescales. Now, because the force field is polarizable, there will necessarily be a cross-correlation between the exciton dipole and the water dipole, and I still do not see how this cross-correlation is indicative of anything. What is indeed interesting with regard to the experiment is that a polarizable dipole added to the CNT has a much greater effect on diffusion than a frozen dipole. The paper is already dense and information-rich: I suggest dropping the cross-correlation analysis (and the discussion of phonons that comes with it) and only presenting the MSD analysis of the simulations – which is sufficient to make the point!

4) It very interesting that dipole moment fluctuations impact diffusion, while a static dipole does not – and I agree that this is qualitatively in line with the experimental observation that pinning the exciton suppresses the effect on diffusion. Thus, one may wonder whether the average dipole moment is at all important. Indeed, excitons in pristine CNTs should rather have no net dipole moment. I would suggest carrying out a simulation with a dipole-free polarizable exciton (e.g. three charged rings, +,-,+) to definitively confirm the role of fluctuations versus static charge distribution.

5) "Single value decomposition" – shouldn't it be "singular value decomposition"?

Version 3:

Reviewer comments:

Referee #1

(Remarks to the Author)
I thank the authors for diligently addressing my final remarks, and I am looking forward to seeing their paper published.

Open Access This Peer Review File is licensed under a Creative Commons Attribution 4.0 International License, which permits use, sharing, adaptation, distribution and reproduction in any medium or format, as long as you give appropriate credit to the original author(s) and the source, provide a link to the Creative Commons license, and indicate if changes were made.
In cases where reviewers are anonymous, credit should be given to 'Anonymous Referee' and the source.
The images or other third party material in this Peer Review File are included in the article's Creative Commons license, unless indicated otherwise in a credit line to the material. If material is not included in the article's Creative Commons license and your intended use is not permitted by statutory regulation or exceeds the permitted use, you will need to obtain permission directly from the copyright holder.
To view a copy of this license, visit https://creativecommons.org/licenses/by/4.0/

Response to Reviewer Comments

We thank the reviewers for their insightful comments. We have addressed them in detail as follows. All newly added figures in the revised manuscript are named with an R (e.g., Figure R1), and additionally, the name in the revised manuscript or supplementary information (e.g., Figure S7) is provided for your convenience.

Referee 1:

1) “The conclusion that CNT diffusion in water can be modulated by pumping them into an electronically excited state is a very significant one, given the growing interest in controlling nanoscale fluid transport using the confining wall's electronic properties. However, the authors go even further and conclude that their "study demonstrates that quantum friction between SWCNTs and water exists". I think that the reported data is insufficient to serve as a proof of water-CNT quantum friction. I recommend that the authors revise their manuscript to draw only the conclusions that can be supported by the data.”

Response: We thank the reviewer for the insightful comments. We added a substantial amount of new experimental and simulation data and refined the conclusions. Additionally, we made an effort to clarify the relation between quantum friction, as previously reported theoretically (Kavokine et al. Nature 2022[1]), and exciton-induced friction (see specific questions and answers below).

2) “The authors seem to be unclear on the definition of quantum friction. The abstract begins with "quantum friction describes the transfer of energy and momentum from electronically excited states in a material to a surrounding solvent". As introduced in Ref. 2 of the manuscript, quantum friction rather refers to a direct transfer of momentum between a liquid and a solid's electrons. Importantly, the solid does not need to be put in an electronically excited state for there to be quantum friction: it is rather the interaction between thermal fluctuations of the solid and the liquid that mediates the momentum transfer. If demonstrated, quantum friction involving a solid in an excited steady state (here, CNT with an exciton population) would be a new and interesting phenomenon. Now, the conclusion states "our study experimentally demonstrates that quantum friction i.e. light-induced slowing down of Brownian motion exists". This is inconsistent with the introductory statements and the cited references.”

Response: Thank you very much for pointing this out. We completely agree that direct transfer of momentum/energy is defining quantum friction (as per reference 2) and does not

necessarily require electronic excitation. If an optically excited state were to interact accordingly, it would also fall under the definition of quantum friction. We have carefully refined the introduction and discussion of quantum friction and the conclusions:

Abstract

“Quantum friction describes direct transfer of energy and momentum between electronic states in a material and a surrounding solvent. Here, we show that near-infrared (NIR) fluorescent single-walled carbon nanotubes (SWCNTs) exhibit quantum friction in water. The diffusion constants of functionalized SWCNTs in aqueous solution decrease linearly by around 50 % with increasing excitation power. In contrast, SWCNTs with quantum defects that localize excitons show no power-dependent diffusion. Chemical manipulation of exciton concentration by molecules that increase or decrease SWCNT fluorescence also modulates the diffusion constant by a factor of up to 2. Optical pump Terahertz (THz) probe spectroscopy reveals transient absorption features of water (37 cm⁻¹ and above 80 cm⁻¹) on time scales of 0.7 ps and 100 ps, indicating energy dissipation from the exciton into translational modes of its hydrogen bond network. Molecular dynamics simulations further support a mechanism in which the fluctuating dipoles of excitons enhance frictional forces. These findings establish light-induced quantum friction between excitons in SWCNTs and water.”

Introduction

…It describes non-adiabatic coupling between collective modes of solvent dipoles and electronic modes of materials such as graphene, graphite, and carbon nanotubes (CNTs).[2–4] The friction is expected to increase when the substrate’s surface response function overlaps with the low-frequency spectrum of the solvent, including librational, intermolecular stretch, and Debye modes.[3]

Experimental studies have provided evidence supporting coupling beyond the Born-Oppenheimer approximation between classical water dynamics and the quantum dynamics of confined delocalized electrons. This is demonstrated by anomalies in hydrodynamic friction at water–carbon interfaces [5,6] and by the rapid cooling of hot electrons in graphene in water.[7] Optical pump Terahertz (THz) probe experiments showed very short time scales of cooling via quantum friction and it was attributed to spectral overlap of graphene surface plasmon modes and water charge fluctuations in the frequency range of the librational mode of water in the THz region.[7] The water–graphene quantum friction force is negligible if the graphene electrons are at rest, but becomes significant if they are driven at high velocity by phonons or an applied voltage.[8] In general, THz spectroscopy can serve as a powerful tool for probing the hydration shell,[9] effectively capturing dynamic solute-solvent interactions, including those driven by charge fluctuations.[4] Notably, these charge fluctuation-driven interactions extend beyond the primary hydration shell,[10] influencing broader solvation dynamics. The anomalously high water friction on graphite as well as the unique slippage behavior observed in CNTs has been attributed to THz plasmon modes.[2,3] Additionally, the water-carbon interface demonstrates remarkable thermal boundary conductance, exceeding that of other solid-liquid systems. This emphasizes the vital role of plasmon-hydron coupling in the THz frequency range for properties of graphene-based materials [7]

Semiconducting single-walled carbon nanotubes (SWCNTs) are one-dimensional nanomaterials that fluoresce in the near-infrared (NIR) tissue transparency window.[12,13] Their fluorescence is best described by electron/hole pairs called excitons,[14] which diffuse along the axis of the SWCNTs for around 100 nm.[15,16] Because the size of excitons is larger than the diameter of SWCNTs, they are significantly affected by changes in the surrounding dielectric environment caused by bundling,[17] surfactants,[18] or DNA wrapping.[19] SWCNTs are inherently hydrophobic,[21] but adsorption of surfactants, peptides, proteins,[22,23] or π-stacking of nucleic acids [24] makes them water soluble. Another way to change their surface chemistry is covalent functionalization, which introduces a low number of σ-bonds into the sp^2 hybridized carbon lattice (sp^3 quantum defects). They act as local traps for excitons and create novel photophysics [25–28]

The optoelectronic properties of SWCNTs are highly sensitive to their chemical environment, which makes them ideal building blocks for (bio)sensors[21] that are able to image chemical signaling by cells,[29,30] for cancer or pathogen diagnostics,[31,32] or to image plant stress.[33,34] The fluorescence changes (i.e. exciton decay or energy shift) of such biosensors have been attributed to conformational changes and changes in local solvation.[4]"

Conclusion

"Our study demonstrates light-induced friction between SWCNTs and water, which can be attributed at least in parts to quantum friction. Exciton-water coupling generates a drag force that slows down the Brownian motion of SWCNTs. Since Einstein's seminal work on diffusion, we know that thermal energy is linked to the stochastic movements of Brownian motion. Light-induced quantum friction changes diffusion constants by a factor of up to two. We demonstrated that either physical (light intensity) or chemical (molecules that change the fluorescence quantum yield) manipulation changes friction (Figures 1 and 2). This observation points to a central role of the exciton concentration. Additionally, the mobility of the exciton (Figure 3a) and the organic corona between the carbon lattice and water (Figure 3b) determined the magnitude of quantum friction. THz OPTP measurements provided insights into the microscopic origins of this phenomenon and indicated energy/momentum transfer on two different time scales from the exciton to translational and stretching modes of hydrogen bonds in water.[4] MD simulations highlighted that the polarizability of SWCNTs and the dynamic nature of the exciton dipole is central for coupling to the solvent and changes in friction (Figure 4b,c). These findings show that exciton-mediated quantum friction directly affects the motion of a nanoscale object and that there is momentum transfer. It is different from laser traps or optical tweezers because one can chemically change it (Figure 2d), and it takes place at much lower laser or light intensities[52]. The light-induced slowing down of Brownian motion can be physically or chemically manipulated to affect the Brownian movement of a nanomaterial in an aqueous solution. Given the anisotropy of SWCNTs, one can anticipate approaches to control the movement of microswimmers or nanorobots. Additionally, it raises the question of whether other materials that have similar excited state dynamics also exhibit quantum friction in water or other solvents."

3) “Furthermore, quantum friction relies on the interaction between charge fluctuations. The excitons in the CNTs have a static dipole moment, which could be responsible for an increase in friction, consistently with the reported MD simulations – this would then not be quantum friction, but rather dielectric friction, as first introduced in [R. Zwanzig,J. Chem. Phys. 38, 1603 (1963)]. Quantum friction would arise from an interaction between the charge fluctuations due to exciton diffusion along the CNT with the water charge fluctuations. Such a mechanism is supported by the experiments with defected CNTs, but MD simulations suggest a significant effects of a static exciton. Based on the reported data, I think it is difficult to draw a firm conclusion regarding the relative importance of dielectric and quantum friction.”

Response: We agree that dielectric friction could be part of the experimentally observed overall friction. However, dielectric friction is rather friction due to permanent dipoles. An example would be a SWCNT with a positive charge at one end and a negative charge at the other end. In contrast, excitons are fluctuating dipole moments both in time (when the exciton is excited) and space (position along the axis of the SWCNT). Excitons decay in < 1 ns and are highly mobile (diffusion length on the order of 100 nm), which means that there are no permanent dipoles. We extended the analysis and studied the role of polarizability for the exciton (see below). It turns out to be a condition for effective interaction with water. Thus, while a SWCNT (with its organic corona) can potentially experience dielectric friction, light-induced friction is different. We have performed additional experiments and analysis that further support this statement:

a) Additional simulations that address polarizability

In the simulations, excitons are represented by “dynamical” dipoles, and their charge fluctuations occur on a timescale that allows them to couple to the water collective degrees of freedom. We show the exciton dipole fluctuations in the new figure R1/4b. It shows the spatial fluctuations, which make it different from dielectric friction. In a real experiment, there would be additional temporal fluctuations when excitons are created or decay. The new figure R2/(S24) presents the Fourier Transform (FT) spectra of the cross correlation between the dipole moment of the SWCNTs with (polarizable) exciton or without exciton and water dipole moments, illustrating their coupling. The large contributions to the frequency range below 1000 cm^{-1} suggest a coupling to the collective modes of water.

We would like to stress that the dynamical nature of the dipole is a key element to explain the observed slowdown of SWCNT diffusion. We thus simulated:

1. A “dynamical” exciton model (obtained using a polarizable model for the SWCNT). It is compared to
2. A “static” exciton model with a dipole of the same size, but the charges are localized at the carbon atoms and not allowed to fluctuate (obtained using a non-polarizable model of the SWCNT). It reduces dipole fluctuations by orders of magnitude.

The “static dipole” does not couple efficiently to water collective modes (no contribution to the cross correlation as shown in figure R2/(S24). Moreover, a direct comparison between the friction induced by a “dynamical” exciton (coupled to water) and that induced by a “static” exciton (minimally coupled to water) is reported in figure R2/(S24). The friction induced by a “dynamical” dipole (“dynamical” exciton) is 3-4 times larger than the friction induced by a “static” dipole. In the case of a “dynamical” dipole, the friction is the result of a quantum phenomenon, although classically described by the simulation model, namely the coupling of the charge fluctuations to the water's low-frequency modes. In the case of a “static” dipole, only dielectric friction is present as a result of the electrostatic interactions between the “static” dipole and the water charges. We also tested the role of phonons, i.e., the carbon lattice vibrations, in SWCNT-water cross coupling. When blocking the carbon-core vibrations (no phonons), the SWCNT-water cross coupling is enhanced R3/(S25) as coupling (polarizable fix carbon) dissipation through phonons into the water is not possible, and there is more direct coupling of the SWCNT electron cloud to water in X, Y directions. The change in coupling in the Z component in R3/S25 is negligible, as it is constrained due to the periodic nature (Figure S22b) of the SWCNT used to compute the cross-correlation. This shows that there is a direct coupling between the exciton in the SWCNT and water, highlighting the presence of quantum friction. We further added a schematic that gives a better overview of the modelling parameters (see your question 7 below, Figure R12/(S23).

In summary, these new simulation results underscore that polarizability (of a SWCNT) and consequently charge fluctuations (after excitation of an exciton) are central to the observed effects and distinct from dielectric friction.

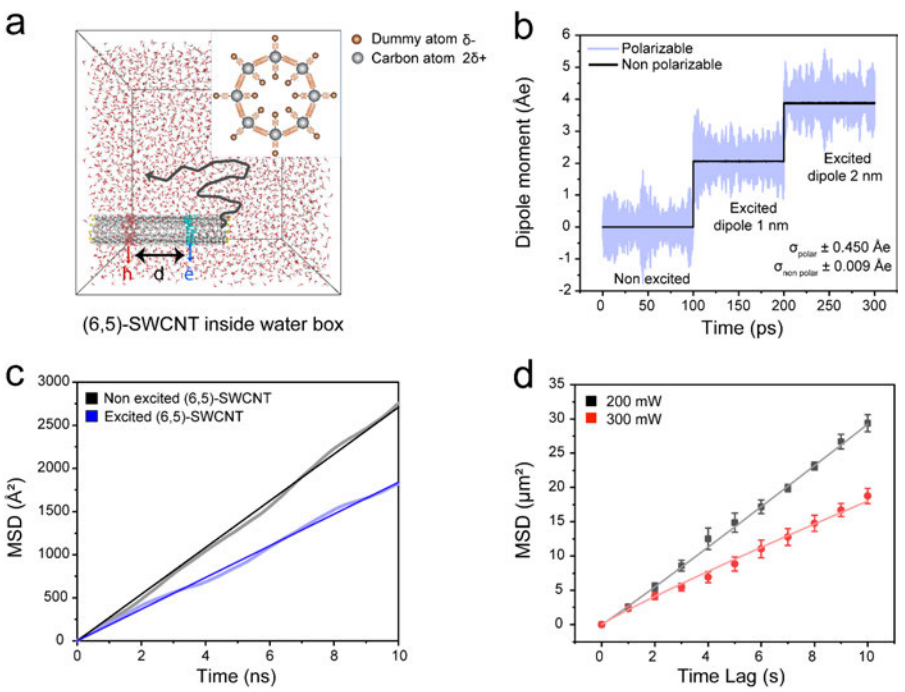

Figure R1/(revised Figure 1). Exciton-related charge fluctuations change diffusion. *a) Molecular dynamics (MD) simulation of excited SWCNTs. A (6,5)-SWCNT (length 4.1 nm) is allowed to move freely (black trace) in a three-dimensional periodic water box. The ends of the SWCNT are capped with hydrogens (yellow). Excitons are modelled by placing charges in the blue (e, electron) and red (h, hole) annular regions separated by the distance. Inset: Schematic of the SWCNT cross-section. For each carbon atom (grey, charge 2δ+), two dummy/virtual atoms (brown, charge δ- each) are placed at a distance of 0.6 Å to mimic the electrostatics of a π-electron cloud. The virtual atoms provide polarizability in response to solvent and ion dynamics. In the current model, a charge of Δδ=0.22e was chosen for the exciton to account for non-excited time periods. b) Dipole moment fluctuations of polarizable (blue) and non-polarizable (black) models of excitons in SWCNTs (with and without excitation). Sigma denotes the standard deviation of the fluctuations. c) Simulated mean square displacement (MSD) curves (semi-transparent) and linear fits for both excited (with excitons) and non-excited (without excitons) diffusing SWCNTs (d=2 nm). d) Ensemble time-averaged MSD plots of single DOC-SWCNTs tracked in a wide field microscope (> 5 particles, > 100 frames each) show slower diffusion at higher light intensity (mean ± SE, n=4).*

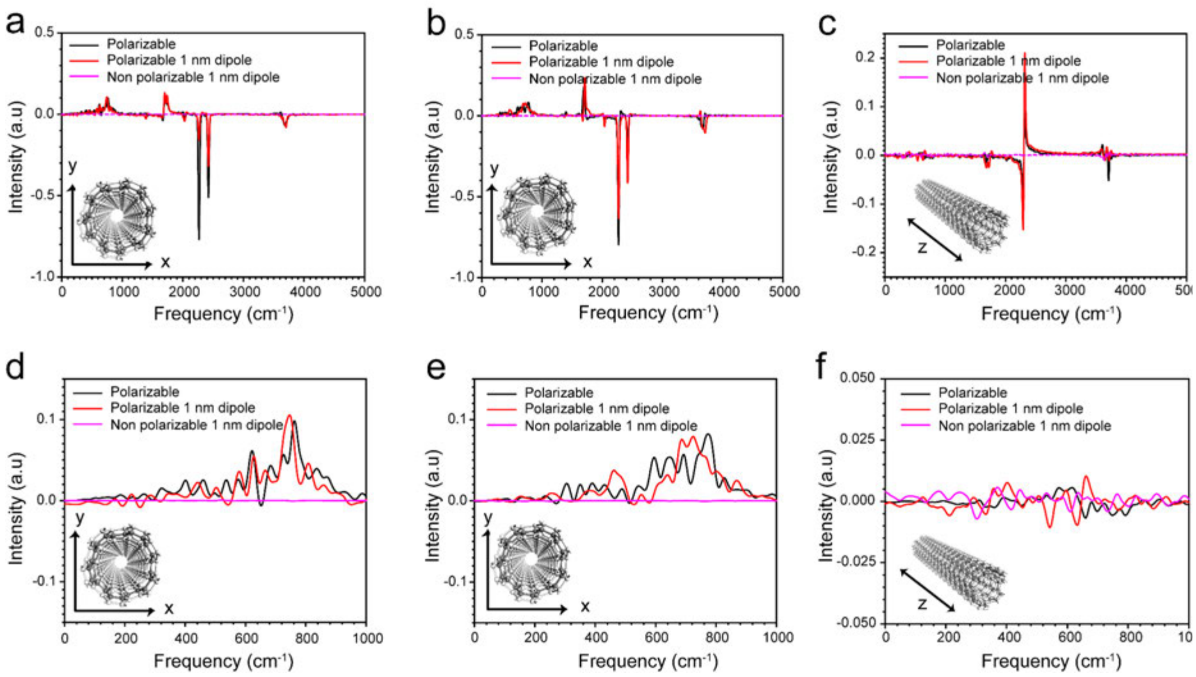

Figure R2 (S24). Spectral cross correlation of dipole moments of SWCNTs and water. (a,b,c) *x, y, and z components of polarizable, polarizable 1 nm dipole, and non-polarizable 1 nm dipole in the range of 0 to 5000 cm^{-1}.* ***(d, e, f)*** *x, y, and z components of polarizable, polarizable 1 nm dipole, and non polarizable 1 nm dipole in the range of 0 to 1000 cm^{-1}. Panels (a, d), (b, e), and (c, f) show the cross correlation in x, y, and z components, respectively. The cross correlation shows a large effect for < 1000 cm^{-1}, which is consistent with the collective modes of water.*

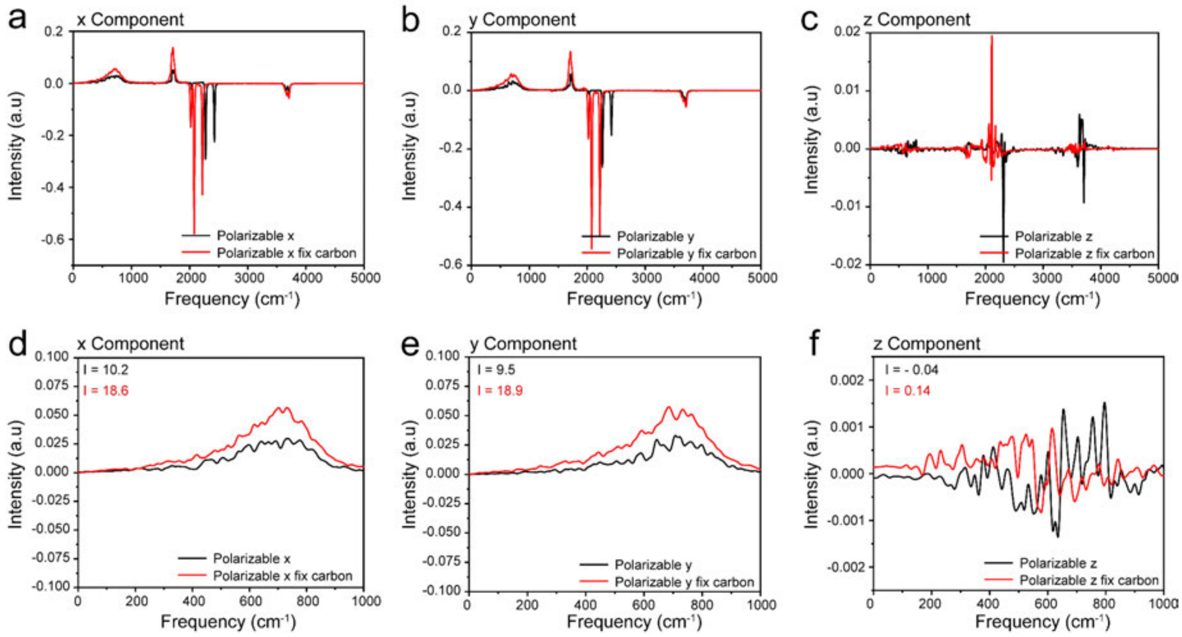

Figure R3 (S25). Intensity of spectral cross correlation of SWCNTs with water is shown for polarizable models with and without phonons (fixed carbon atoms). *In the polarizable case, both C-core and dummy atoms are allowed to vibrate, but in the polarizable fixed (fix) carbon case, the C-core is fixed and not allowed to move, while the attached dummy atoms are allowed to vibrate. Panels a-c show the cross correlation of SWCNT with water in X, Y, and Z components, respectively, and panels d-f show the zoomed in view of the same (inset values: I (black) =* $\int f_{cross\ section\ polarizable\ x}\ df_{req}$ *and I (red) =* $\int f_{cross\ section\ polarizable\ x\ fix}\ df_{req}$*). The integral of the two curves between 0 to 1000* cm^{-1} *wave numbers shows that the cross correlation effect between SWCNT and water is almost doubled in the case of X and Y, while the change is minor in the case of the Z component. In the case of electron-phonon coupling (polarizable), part of the energy of the electrons can be directly dissipated in the phonon, while without coupling (polarizable fix carbon), dissipation through the phonon is not possible, and there is more direct coupling to water. The effect in the Z component is negligible as it is constrained due to the periodic nature (Figure S22b) of the SWCNT used to compute the cross-correlation.*

b) Analysis and interpretation of THz data

In our opinion, one strength of our work is that the findings are further supported by spectroscopic evidence. In order to clearly reveal the (at least partial) contribution of quantum friction, we carried out an Optical Pump THz Probe (OPTP) study of SWCNTs in water. Previously, OPTP spectroscopy was used to prove quantum friction between water and graphene sheets.[6] An optical pump excitation pulse at 400 nm was used to quasi-instantaneously create heated electrons in graphene. The THz probe was centered in the frequency range below 1 THz, and the authors recorded the amplitude of the electrical field of the THz beam as a function of pump-probe delay. The transient signal was attributed to a direct cooling of the electron temperature, which manifests in a decrease of conductivity and thus in a decrease of the amplitude of the THz probe. The authors observed in water and deuterated water a faster cooling time of 1.4 ps to 2.0 ps, as in other polar liquids. This was taken as proof of quantum friction, since the hot electron cooling occurs via direct coupling of the electrons to the solvent.

In our study, we also pump the SWCNTs (in the THz experiments) via a pump laser at 400 nm, but we create excitons. Most importantly, the THz absorption increases (positive) in all measurements, which is the first proof of the interaction between the exciton or its decay pathways with water. The THz probe response is plotted in figure S21 and figure 3c,d. Due to several experimental improvements, we can provide not only the transient amplitude of the field but also the fully frequency resolved THz probe response in the entire frequency range below 200 cm^{-1}. This allows us to probe the THz exciton modes (< 50 cm^{-1}) as well as the hydrogen bond stretch water modes (< 200 cm^{-1}). Experimentally, we observe instantaneously -within experimental uncertainty - an initial absorption centered below 50 cm^{-1}(Figure S21c) i.e., where the coupling of the exciton and water should be most prominent. This response decays exponentially with a time constant of 0.7 ps (Figure 3c) similar to that for graphene[6], which was attributed to the cooling of electrons via quantum friction. Thus, we attribute this fast decay at least in parts to quantum friction between SWCNTs and water.

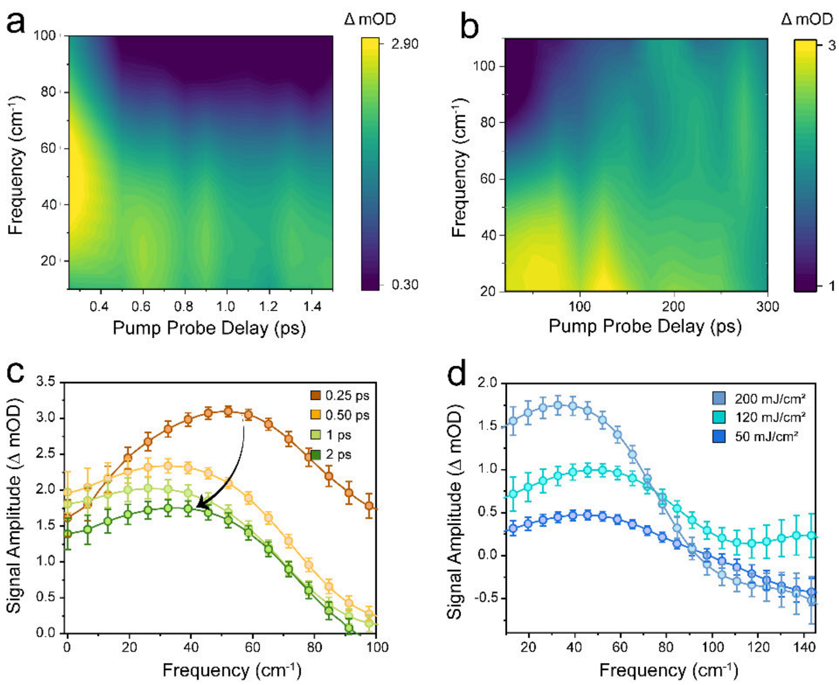

Figure S21. THz spectroscopy of DOC-SWCNTs in water: Temporal dynamics and fluence dependence. *a,b) The measured change in THz absorbance (Δ mOD) upon optical excitation versus without exciton excitation (400 nm) is plotted against pump-probe delays. c) Transient THz spectra (corresponding to vertical slices of the map at representative pump-probe time delays (0.25, 0.5, 1, and 2 ps). The arrow serves as a guide to the eye. d) Comparison of transient THz spectra at a pump probe delay of 2 ps for distinct fluences (200, 120, and 50 mJ/cm^2) (mean ± SE, n=22).*

Interestingly, at pump probe delays exceeding 100 ps, we find an additional response in the frequency range of the hydrogen bond stretch band, i.e., above 100 cm$^{-1,}$ which can be assigned to a hydrogen bond stretch mode or translational modes of the hydration water (Figure 3d). This is also the response of the hydrogen bond network in the frequency range of acoustic phonons. Thus, this delayed response could also be attributed to phonon hydration coupling. The expected time scale for the (Debye) relaxation time of a dipole moment is expected to scale with the length of the dipole moment. For bulk water, the time scale is 8 ps. Larger solutes like the SWCNTs are expected to have longer relaxation times due to increased rotational inertia and interactions with the solvent.

We have moved the more concise short time scale analysis into figure 3c and the full (original) 2D supplementary figure to SI figure S21 to have both time scales side by side, which we hope makes understanding easier.

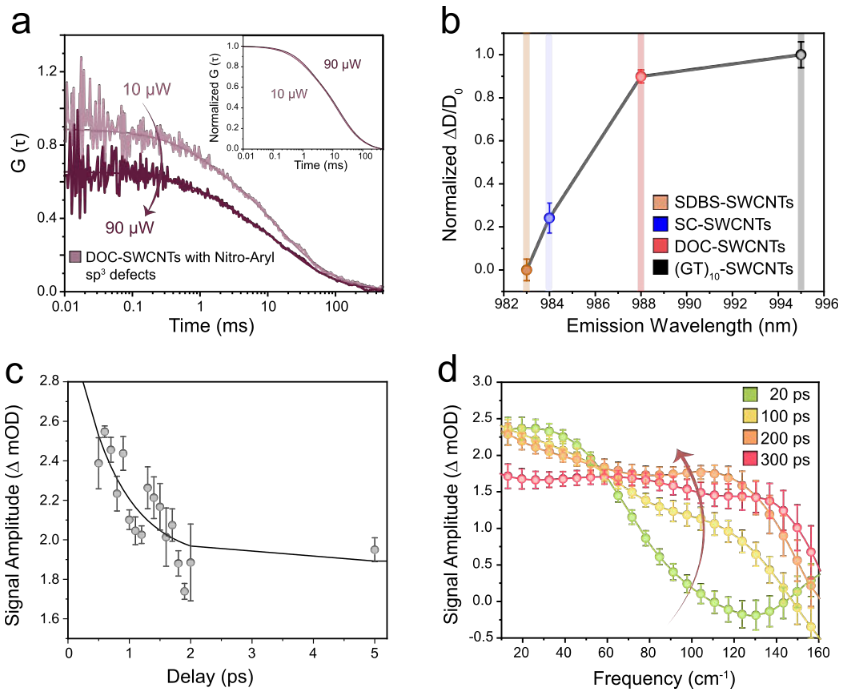

Revised figure 3. The organic corona shields quantum friction and excitons change THz absorption by water. *a) Excitation power-dependent fluorescence autocorrelation functions of DOC functionalized SWCNTs (DOC-SWCNTs) with Nitro-Aryl sp^3 quantum defects (wine) that serve as exciton traps show no power-dependent diffusion. The inset shows the normalized and fitted autocorrelation function. b) Normalized diffusion constant changes increase with emission wavelength for different functionalized SWCNTs (mean ± SD, n=3). Lines serve as visual guides. c) Optical Pump Terahertz Probe (OPTP) measurements of an aqueous DOC-SWCNTs solution. Plot of the maximum amplitudes of Δ mOD at a given initial time delay below 60 cm^{-1}. The fitted exponential is also shown as a solid line, yielding a decay time of 0.71 ± 0.24 ps. (mean ± SE, n=22). d) Vertical slices at representative pump-probe time delays (the arrow serves as a visual guide to the eye).*

Based on the time-resolved THz experiments, we thus propose that there are two distinct pathways through which the SWCNTs dissipate energy after excitation:

1) On the 0.7 ps time scale a rapid response is observed. Similar to (hot electron cooling in) graphene,[7] we attribute this fast decay to quantum friction between SWCNTs and water.

2) On the time scale > 100 ps corresponding to the exciton lifetime, we observe (Figure 3b) translational THz modes in the water network, which are attributed to momentum transfer via phonon hydration water coupling that slows down SWCNTs.

Both processes can be attributed to a mixture of quantum friction and classical friction (via phonons), which is supported by the simulations (see below).

However, not by a permanent dipole because excitons fluctuate in space and time. As observed in previous papers on graphene, both processes will contribute to energy dissipation

and are not easy to disentangle unambiguously. As discussed above, the simulations demonstrate qualitatively that without phonons, there is also coupling to water (Figure R2/(S24), R3/(S25)). Additionally, the success of the chemical manipulation experiments (Figure 2) indicates that the slower timescale significantly contributes to the friction because it takes place after the first picoseconds (>100 ps), when the excitons have already lived for a while). We have specified these different aspects in the revised paper.

4) “What is the purity of the samples in terms of nanotube chiral indices? Can artifacts due to the presence of metallic nanotubes in the sample by excluded ? ”

Response: In our study, we primarily used (6,5)-chirality enriched SWCNTs (SG65i, produced by the CoMoCAT process). These materials contain > 90 % (6,5) SWCNTs as shown by the 2D excitation and emission spectra (Figure S4) and absorption spectra (Figure S1/S2). Metallic SWCNTs are known to show features in the 400-550 nm and UV range in the absorption spectrum[12,13], but we did not observe these features (Figure R4(S2)). Thus, if there were metallic SWCNTs, they would be abundant at a very low concentration, and the indirect (e.g., quenching) effect on the fluorescence-based measurements is expected to be minimal.

We additionally performed experiments with samples that were further purified using aqueous two phase separation (ATPE) (Figure R4/(S2), R5/(S3)). As evidenced by the absorption spectra (Figure R4/(S2)), only minor peaks from other chiralities are observed. The absence of cross-peaks in the 2D spectrum, as well as the absorption spectra, indicates that <2 % of other single-walled carbon nanotubes (SWCNTs) are present, as determined by fitting multiple Gaussians to the data. (Figure R5/(S3)).

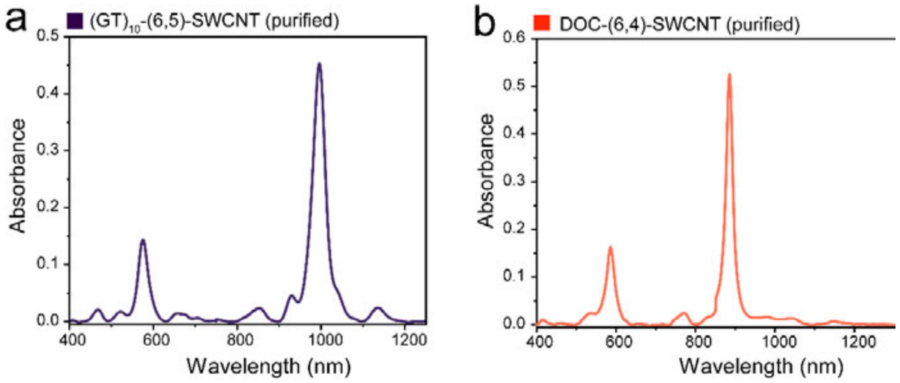

Figure R4 (S2). Absorbance spectra of chirality enriched SWCNT samples*. a) $(GT)_{10}$-(6,5)-SWCNTs b) DOC-(6,4)-SWCNTs. Samples were purified with ATPE.*

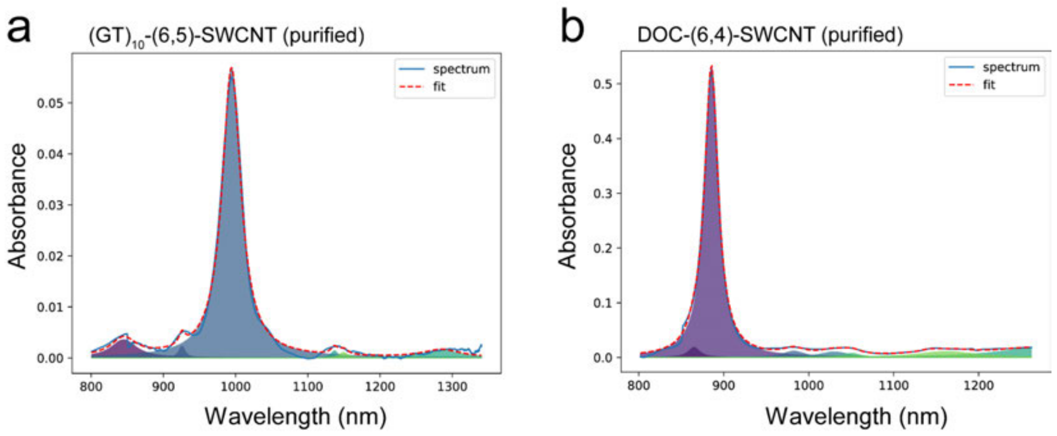

Figure R5 (S3). Fitted absorbance spectra of chirality enriched SWCNT samples (E_{11} region). *a) $(GT)_{10}$-(6,5)-SWCNTs b) DOC-(6,4)-SWCNTs. The fit is based on multiple Lorentzians and minimizing the difference to the data.*

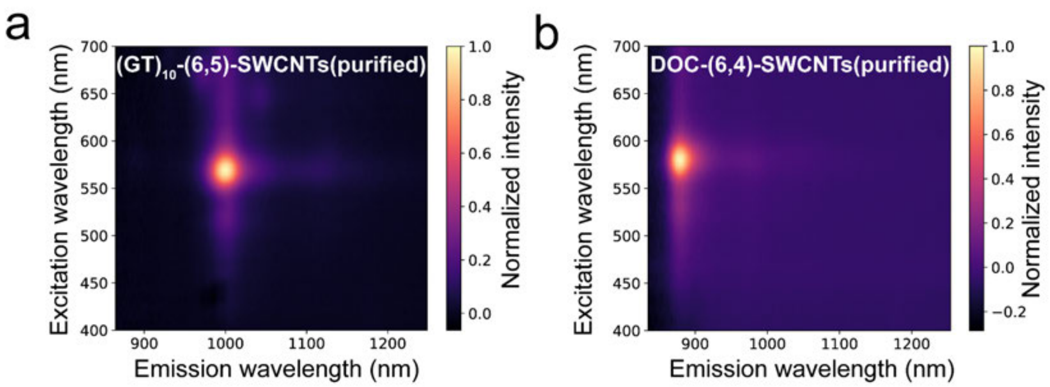

Figure R6 (S5). 2D excitation-emission spectra of enriched SWCNT samples*. a) $(GT)_{10}$-(6,5)-SWCNTs b) DOC-(6,4)-SWCNTs*

We also performed FCS measurements of the purified $(GT)_{10}$-(6,5)-SWCNTs and found that the diffusion behavior was similar to that of the normal $(GT)_{10}$-SWCNTs (Figure R7/(S11)).

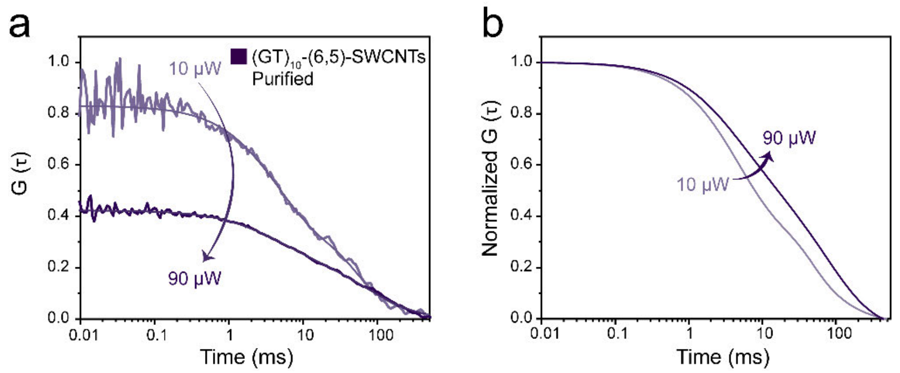

Figure R7 (S11). Influence of excitation power on fluorescence autocorrelation functions of purified $(GT)_{10}$-(6,5)-SWCNTs. *a) Autocorrelation and b) Normalized autocorrelation of purified $(GT)_{10}$-SWCNTs with 10 µW and 90 µW.*

Table R1 (ST2). Purified $(GT)_{10}$-(6,5)-SWCNTs (1 nM). *FCS fitting parameters with 10 and 90 µW power (mean ± SD, n=3).*

Power (µW)	τ_d (ms)	D (μm^2/s)	τ_t (ms)	T	β	Brightness	N
10	16 ± 1.0	1.5 ± 0.02	2 ± 0.6	0.3 ± 0.007	0.72 ± 0.002	3044 ± 200	1.6 ± 0.02
90	25 ± 1.1	1.0 ± 0.05	2	0.3	0.42 ± 0.008	7010 ± 301	4.0 ± 0.20

To summarize, our samples were very pure and contained, if at all, very low amounts of metallic SWCNTs. Further purification did not change the FCS patterns. Thus, we conclude that there is no significant interference by metallic or other chirality SWCNT species.

5) “FCS monitors nanotubes as they enter and exit a confocal volume. But the exciton population in a nanotube is maintained in a steady state only when the nanotube is inside the confocal volume. Thus, the diffusivity measurement and the dynamics of the exciton population are entangled. I believe that a measurement where this is not the case is required for the conclusion to be unambiguous. The authors do carry out a macroscopic viscosity measurement, but only with a (putative) chemical modulation of exciton population. Could the authors monitor the viscosity (and temperature) of the CNT solution under varying 485 nm light intensity? More generally, a diffusivity measurement with no light intensity gradient would help make the conclusion more robust”.

Response: We agree that there is an entanglement between diffusion and exciton dynamics in a FCS measurement geometry. The macroscopic viscosity measurements by µm-sized beads in a solution of SWCNTs served as a ‘simpler’ proxy because wide-field imaging of the fast diffusion of SWCNTs is very difficult due to diffusion in the z-dimension. We realized now that the macroscopic bead tracking experiments are indeed more complex. Quantum friction changes the diffusion of the SWCNTs, but this does not necessarily translate into a linear

relation to macroscopic viscosity and could be strongly concentration dependent, as for other semiflexible polymers.

We now performed single SWCNT experiments in a non-confocal wide-field geometry that show the same pattern as the FCS results (Figure R17/(4d)). Please refer to Reviewer 2 (response 7) for a detailed explanation of the challenges in particle tracking. Even though the bead experiments in SWCNT solutions are highly interesting, we decided to put them in the supplementary information now because of their more complex interpretation (see Reviewer 2 response 7).

We also confirmed that the temperature of the samples remained consistent at both 10 µW and 90 µW (Figure R8/ (S11)). It shows that heating is not the reason for the observed changes in diffusion. We want to note that even if there were heating, one would actually expect the opposite effect on diffusion (decrease of water viscosity with increasing temperature, leading to faster diffusion).

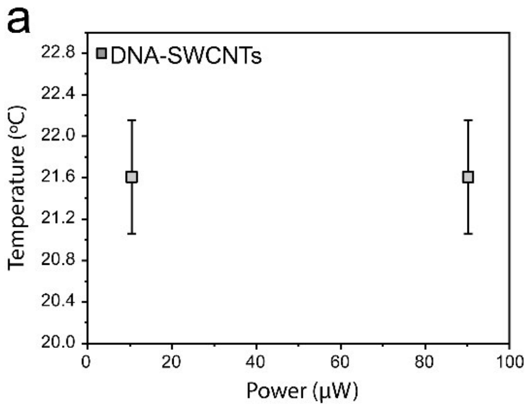

Figure R8 (S11). Temperature remains constant across power levels: *A comparison of measured temperatures at 10 µW and 90 µW laser power of $(GT)_{10}$-SWCNTs in PBS buffer (n=5, mean ± SD). Temperature sensors recorded the temperature before and after the experiment under two distinct powers.*

Additional FCS Simulation

Additionally, we created a simulation for the FCS experiments (Figure R9/(S18)). It simulates a random walk in a water box with a laser confocal volume. Excitation happens only inside this volume, considering the laser profile and polarization. It allows one to calculate the number of excitons, and from this one can calculate the emission, as well as include exciton concentration dependent quantum friction. This simulation shows that one can reproduce the experimental autocorrelation curves (Figure R10/(S19)). In the future, one could use it to

predict more complex behavior, such as anisotropic diffusion and the influence of light polarization on certain time scales. To account for exciton induced friction, the translational and rotational diffusion coefficients were adjusted in each simulation step according to the instantaneous exciton generation rate. The current exciton generation rate (R_{Power}) was calculated from the laser intensity within the confocal volume and normalized by the reference excitation rate (R_{ref}) corresponding to a laser power of 90 µW. We observed experimentally a decrease by around 50 % for the diffusion constant (figure 1), which is thus reproduced by this linear relation:

$$D_{eff} = D_0(1 - 0.5 \times \frac{R_{exciton}}{(R_{exciton,\ ref} \times 0.5)})$$

where D_0 is the diffusion coefficient in the absence of excitation-induced effects. Note that the second 0.5 factor is due to the (mean) orientation dependent excitation of SWCNTs when using polarized light. This very simplified approximation assumes a proportional decrease in diffusion with increasing exciton concentration and can be refined and other hypotheses tested in future work. For example, the different diffusion constants (translation, rotation, long axis, short axis) could be affected in different ways. Further details of the FCS simulation have been added to the supplementary information.

The current version of the FCS/random walk simulation Python code has been uploaded to our university's cloud and is available without a password for the review process: https://ruhr-uni-bochum.sciebo.de/s/3FFgJEGbG8tpgX9

If the manuscript gets published, we will provide a public GitHub link.

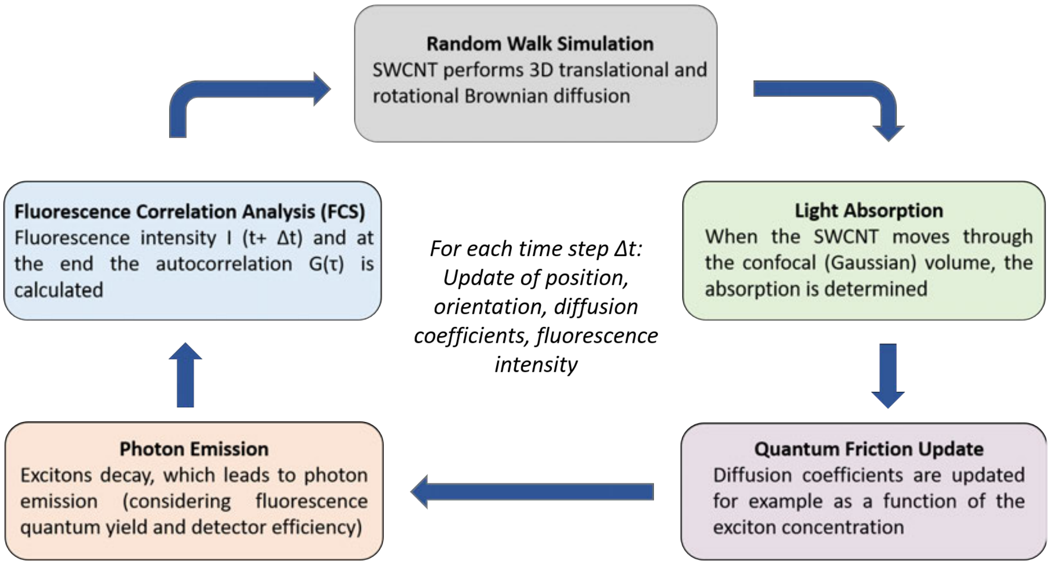

Figure R9 (S18). Schematic workflow of the SWCNT fluorescence correlation spectroscopy simulation. *This schematic outlines the SWCNT FCS simulation. It models the SWCNT's three-dimensional translational and rotational Brownian diffusion. As the SWCNT moves through the confocal Gaussian detection volume, the simulation calculates light absorption. The resulting exciton concentration can affect the quantum friction and thus diffusion constants. Photon emission is simulated according to exciton decay, fluorescence quantum yield, and detector efficiency. The resulting fluorescence intensity trace is analyzed to derive the autocorrelation function G(τ).*

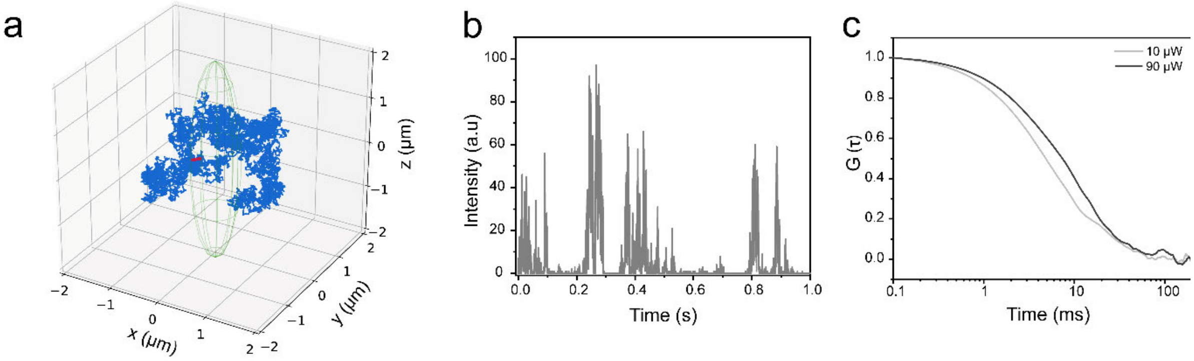

Figure R10 (S19). Simulated diffusion of a SWCNT within the confocal volume. *a) Simulated diffusion of a 600 nm (6,5)-SWCNT through a confocal volume (effective volume: $\omega_0 \approx 0.48$ μm, $z_0 \approx 2.21$ μm; simulation box here: 4 × 4 × 4 μm³). a) Exemplary simulated trajectory (blue) of a SWCNT diffusing through the confocal volume (green wireframe). Orientation of the SWCNT at the last time point is shown in red. b) Corresponding fluorescence intensity trace. c) Exemplary autocorrelation functions for 10 μW (grey) and 90 μW (black) excitation power. The shift to the right (slower diffusion) fits qualitatively the experimental results (e.g., in Figure 1).*

6) “What is the role of laser pulsing in the FCS measurements”?

Response: The use of a pulsed laser allows time-correlated single-photon counting (TCSPC). The original motivation was to perform the first FCS experiments of SWCNTs and also to use the fluorescence lifetime information. During these experiments, we noticed the strong light intensity dependence and identified the link to quantum friction (Figure 1). Autocorrelation analysis can be conducted using either continuous-wave (CW) or pulsed excitation, but the latter offers additional information. Additionally, pulsed excitation/TCSPC helps eliminate environmental background fluorescence and reduces artifacts like afterpulsing, and should therefore improve signal-to-noise ratios, leading to more accurate correlation curves.[14,15]

Therefore, most of our FCS measurements have been performed with pulsed excitation. However, we obtain qualitatively the same results with CW excitation (Figure R11/(S17)). We also did not see non-linear effects (exciton annihilation) at the used light intensities (Figure S10). Thus, we conclude that the mode of excitation does not affect the results (for the used range of light intensities and pulse rates).

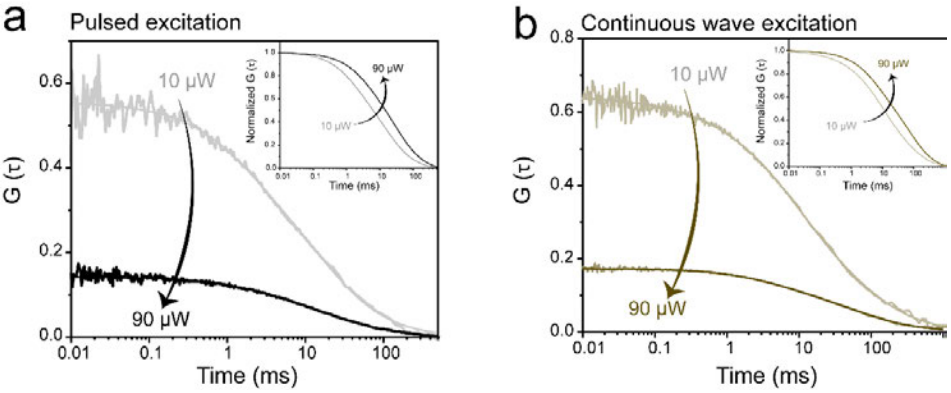

Figure R11 (S17). Comparison of pulsed and continuous laser excitation in (GT)$_{10}$-SWCNT at low (10 µW) and high (90 µW) excitation power. *(a) Autocorrelation trace for pulsed excitation. (b) Autocorrelation trace for continuous wave (CW) excitation. Inset: Normalized curves demonstrating the impact on diffusion.*

Table R2/ (ST13). Comparison of pulsed and continuous laser excitation in (GT)$_{10}$-SWCNT: *FCS fitting parameters with 10 and 90 µW power (mean ± SD, n=3).*

Power (µW)	τ_d (ms)	D (µm^2/s)	τ_t (ms)	T	β	N	Brightness
**Pulsed**							
10	14 ± 1.02	1.60 ± 0.07	2.0 ± 0.15	0.28 ± 0.008	0.80 ± 0.012	3 ± 0.31	1138 ± 400
90	24 ± 1.21	0.92 ± 0.06	2.0	0.28	0.47 ± 0.026	9.3 ± 0.21	4500 ± 300
**Continuous**							
10	14 ± 0.08	1.60 ± 0.023	2.0 ± 0.078	0.31 ± 0.021	0.70 ± 0.002	2.0 ± 0.10	2015 ± 200
90	25 ± 0.98	0.90 ± 0.03	2.0	0.31	0.53 ± 0.013	7.0 ± 0.18	6290 ± 398

This makes sense because the pulses on the <100 ps time scale, exciton decay (ns), and diffusion (ms) are on very different time scales. We also estimated the exciton concentration for the used conditions in FCS experiments and the new wide field SWCNT tracking experiments (Figure 4d, see reviewer 2 response 7). The overall exciton concentration appears to be on the same order of magnitude. The calculated exciton number for the FCS experiments increases from 2.7 to 24.9 (per pulse, 40 MHz) as the excitation power rises from 10 µW to 90 µW. Most likely due to optical losses, etc., the numbers are in reality smaller. One can imagine a SWCNT as a chain of segments in which the excitons can behave independently, and the linear power dependence of the fluorescence intensity (Figure S10) supports this picture. The Python code for calculating the exciton number for FCS and wide-field measurements is provided in our university's cloud:

https://ruhr-uni-bochum.sciebo.de/s/3FFgJEGbG8tpgX9.

If the manuscript is published, we will provide a public GitHub link.

7) "MD simulations: even after a detailed reading of the methods and supplementary information, it is not clear to me how the exciton is modeled. What is the total charge of the electron and hole? Is the polarizable force field necessary to draw the qualitative conclusion? From the experimental side, what is the expected exciton concentration in the steady state as a function of light intensity, and how does it compare to the simulation"?

Response: In short, yes, the polarizability is crucial. We have now added more details on the simulations (see Figures and response to question 3 above). The exciton is modelled as an "electron-hole" by adding negative charges (blue ring) and positive charges (red ring) with spatial separation of ~ 1 nm or ~2 nm (Figure R12/ (S23)).

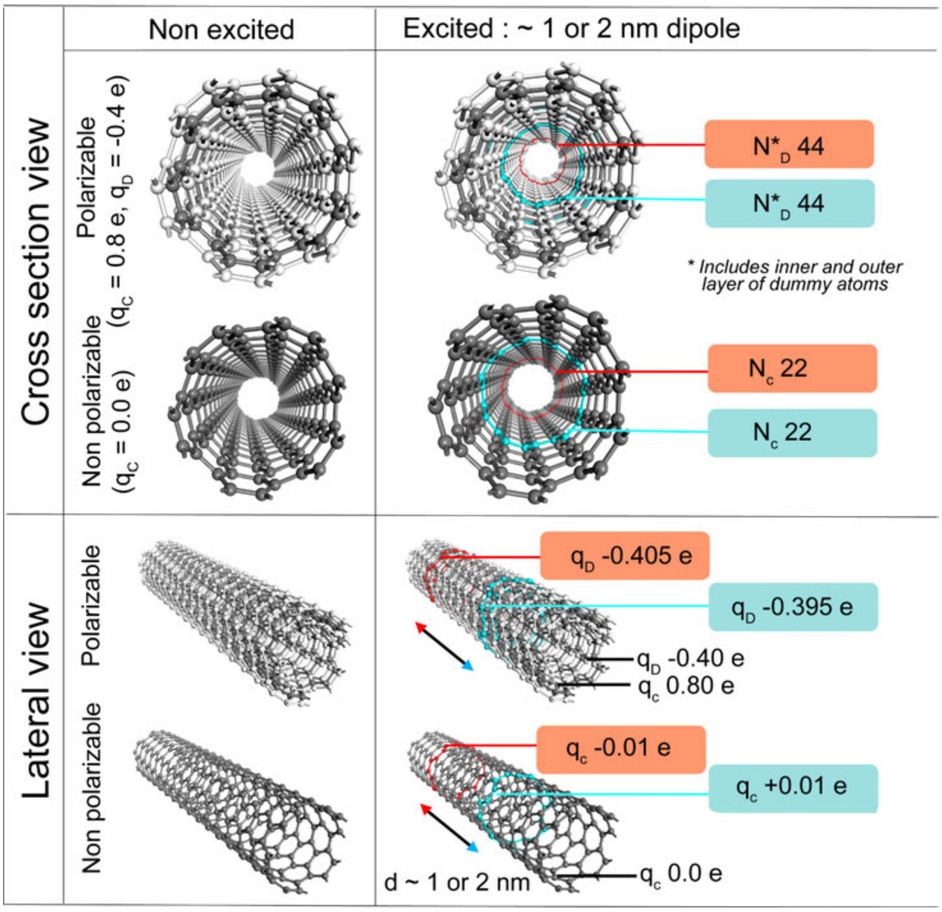

Figure R12 (S23). Design parameters for modelling excitons in SWCNTs: *The periodic box and the water molecules are not shown to reveal the (6,5)-SWCNT and charge distribution to model the exciton in polarizable and non polarizable models with cross sectional and lateral views. N: number of atoms whose charges have been altered to model an exciton. q: charge on the altered carbon, subscript D: dummy atoms charge modified in polarizable model, subscript C: carbon atom charges modified in non-polarizable model. The double headed arrows denote the separation of 1 nm or 2 nm between the positively (red) and negatively (blue) charged annular regions. In the non-excited case or grey regions, the non-polarizable carbon atoms carry a net zero charge, whereas in the polarizable models, carbon-cores carry +0.8 e charge. Two dummy atoms are attached to each carbon core on either side of the surface plane, an arrangement inspired by the pi-electron cloud of aromatic rings. The dummy atoms carry -0.4 e charges in the ground state to maintain charge neutrality of the overall carbon-atom (C-atom = 1 C-Core + 2 Dummy atoms). Upon excitation, the charge on the dummy atom is altered by ±0.005 to create an exciton with the bound state of an electron and a hole pair delocalised over carbon atoms in an annular region separated by a distance d' (d = 1 nm or 2 nm). In other words, the polarizable model creates dummy charges + 0.01 (0.005 inside and outside) at 22 carbon atoms on one side and -0.01 at 22 carbon atoms on the other side. This leads to a charge of + 0.22 e and -0.22 e at a defined distance (1 nm or 2 nm) mimicking an exciton. We chose 0.22 to account for the fact that in the experiments, there are not always excitons present, and the SWCNTs are much longer. However, this is only a parameter and can be changed in the simulation. Note that the 22 carbon atoms represent the simplest geometry to distribute charges in a symmetric way around a (6,5)-SWCNT carbon lattice.*

The spatial separation is based on estimates from the literature (e.g., an exciton size of 2.0 ± 0.7 nm).[16] In particular, the charge density is delocalized over 22 carbon atoms (on ~ 0.75 nm wide ring) or 44 dummy atoms, assigning ± 0.01 (or ± 0.005) elementary charge to each atom depending on whether non polarizable or polarizable models are considered. Analogously, the hole is modelled in the same way, but adding positive partial charges. The electron-hole excitation can be created both in the polarizable and non polarizable model. However, when the exciton is created in the non polarizable model, the charge remains more localized (distributed over the carbon atom only) and the total dipole moment associated with the excitation is "static" with fluctuations (calculated from the standard deviation (σ)) in the order of 0.009 Åe. Instead, in the polarizable model, the dipole fluctuations (after excitation) orders of magnitude larger (σ = 0.45 Åe, (Figure R1/ 4b)). The dynamic nature of the excitons in the polarizable model makes a strong coupling to water possible. This can be seen, e.g., in the cross correlation between the SWCNT dipole (when the exciton is created) and the total water dipole in the simulation box. In the Fourier transform spectrum of such correlation (Figure R2/ (S24)), non-zero contributions can be observed in the frequency range < 1000 cm^{-1}, which corresponds to the water collective modes. This frequency range, and in particular the libration band around 600-700 cm^{-1}, has been used in the THz experiments to derive the time constant for electron cooling.[6] On the other hand, in the case of a "static" exciton, as obtained in the SWCNT non polarizable model, the cross correlation (pink line) is barely visible in the graph reported in figure R2/ (S24). The coupling between the exciton dipole and the water modes results in a highly increased friction coefficient (Figure R13/ (S26)). The z-component of the friction coefficient for the excited polarizable SWCNT is ~3-4 times larger than in the case of the excited non polarizable model.

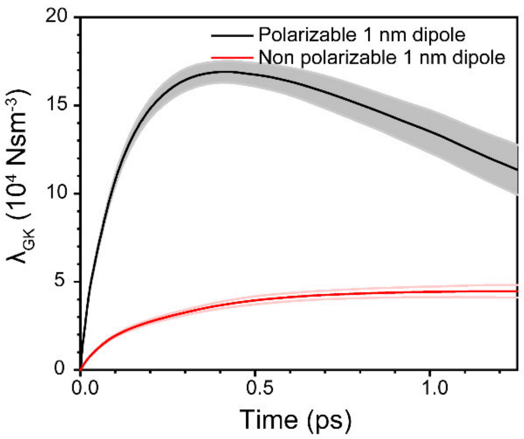

Figure R13 (S26). Comparison of Green-Kubo friction coefficient *for polarizable and non polarizable excited 1 nm dipoles along the z axis of SWCNT.*

This further confirms that the presence of separated electron-hole charge is not a sufficient condition to enhance the friction, and it is indeed the dynamic polarizable nature of the excited state in a SWCNT that is the key element to couple charge dynamics to water dynamics. This is also in agreement with the known long diffusion lengths of excitons in SWCNTs (around 100 nm).

In the simulation, given the relatively small size of the simulation box, a single exciton is considered. Therefore, no interaction between excitons (e.g., annihilation) is considered. In the experiments, we also did not find non-linearities that would indicate exciton-exciton interactions (Figure S10). In the simulation, the single exciton doesn't decay, and it can be considered in a steady state delocalised over carbon atoms in an annular region.

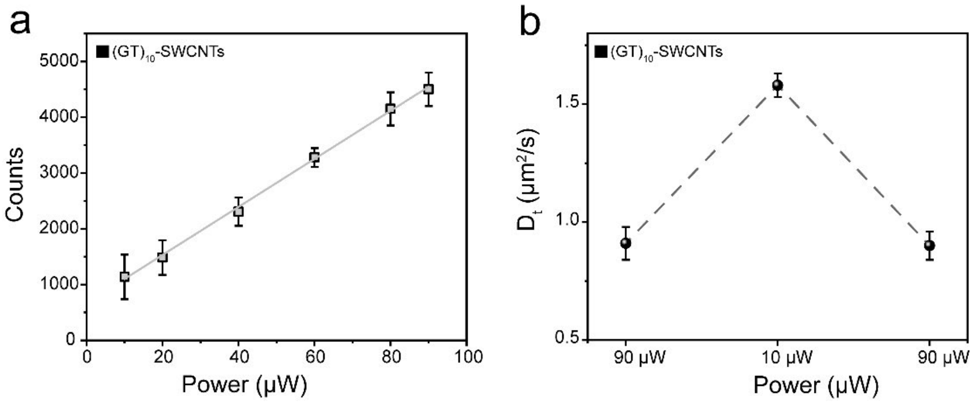

Figure S10. Fluorescence intensity scales linearly with excitation power and the change of the diffusion constant is reversible. *Molecular brightness of $(GT)_{10}$-SWCNTs (1 nM) as a function of excitation power (Microtime 200 setup, λ_{exc}= 480 nm), and fitted by a linear equation (grey) (R2 = 0.997) (n=3, mean ± SD) b) The diffusion constant of $(GT)_{10}$-SWCNTs can be reversibly changed with excitation power (dashed grey lines show a visual representation) (n=3, mean ± SD).*

We estimated the number of excitons with certain assumptions about the FCS geometry[17] in the answer to question 6. Given the short length (4.1 nm) of the SWCNT in the simulation and the fact that the exciton does not decay, its concentration is higher in the simulation (e.g., factor 5 for 90 µW in FCS experiments). Therefore, the simulation mimics the immediate time after a laser pulse, but the CW experiments (figure R11) and the wide field tracking experiments suggest that it is rather the total power and not the timing that causes the slowing down of diffusion.

In summary, for coupling to water, the exciton related charge fluctuations along the SWCNT axis due to its polarizability are crucial.

8) “A minor terminology point: in the introduction “simulations offer insights into the anomalously high water friction on graphite” – this insights rather comes from analytical theory.”

Response: Thank you very much for this hint. We modified this sentence accordingly.

9) ”In summary, I think that the authors report some very interesting data that suggest a coupling between CNT excitons and water dynamics. However, it is not a proof of water-CNT quantum friction and I strongly suggest that the authors tone down the wording in this regard, in addition to addressing the above points.”

Response: We thank the reviewer for the positive assessment. We believe that addressing the questions of the reviewers significantly improved the paper. The combination of more experimental controls, extension and clarification of the simulations, and interpretation of the THz data provides strong support that the excitons couple directly to water. In the revised version, we now discuss this more detailed picture of the possible pathways for energy dissipation.

Referee 2:

1) “The manuscript by Havenith, Sulpizi, Kruss and co-workers describes a series of deceptively simple experiments that observe a puzzling decrease of the diffusion constant of functionalized carbon nanotubes in water. The FCS experiments observe that under optical excitation the CNT particle slow down by up to a factor of two, which is a significant effect. In two key experiments, the observed diffusion coefficient decrease vanishes when the CNTs are functionalized with defects that suppress exciton diffusion and also when water is replaced by heavy water (D2O). These experiments (especially the D2O one) point out to interesting fundamental physics that could be contributing to these results. However, before I become fully convinced, I would like to point out to some additional questions and comments.”

Response: We thank the reviewer for the positive assessment.

2) “I think more characterization of CNTs used in the study is in order. What was the length distribution of the CNTs and how uniform were those tubes. Of course expecting a uniform distribution is not very realistic, but some idea of the length distribution (for example from HR-TEM studies) would be very helpful, as different CNT lengths may affect the exciton dynamics.”

Response: One can expect a length distribution of around 600 nm for such samples, as has been shown before.[18,19] We now added additional characterization by using Atomic Force Microscopy (AFM) because achieving a high contrast in HR-TEM of SWCNTs is challenging (in our experience). The results obtained from AFM are now included in the revised manuscript (Figure R14, R15/ (S6,S7)).

Samples were also characterized by absorption, 1D and 2D fluorescence spectroscopy, showing a very high purity (Figure S1, S4). We also prepared additionally chirality purified samples following question 4 of reviewer 1 and provided a full characterization (absorption, 1D and 2D fluorescence spectroscopy, FCS experiments).

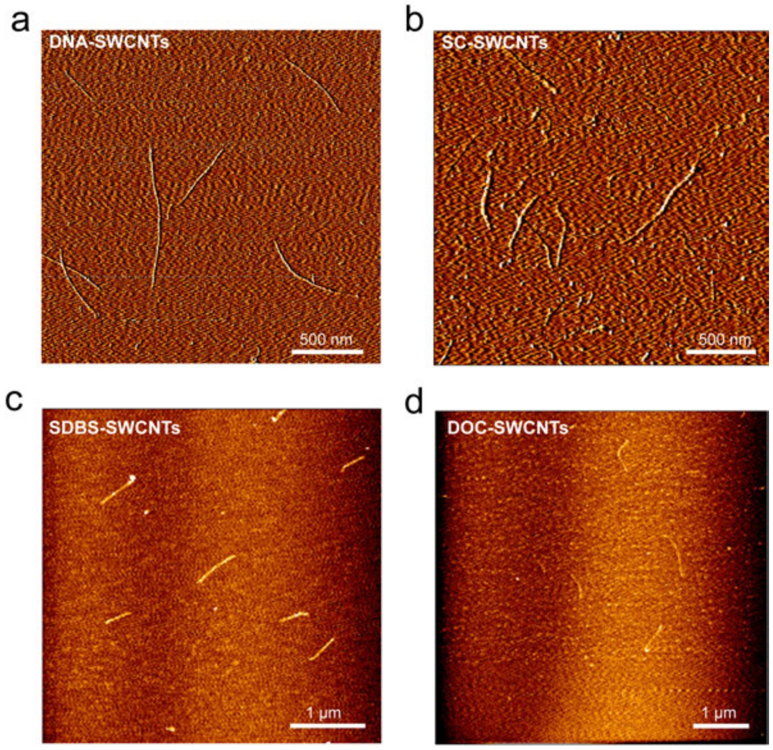

Figure R14 (S6). AFM (Atomic force microscopy) images of SWCNTs. *a) $(GT)_{10}$-SWCNTs, b) SC-SWCNTs, c) SDBS-SWCNTs, d) DOC-SWCNTs. Note that images a and b were captured using the AC tapping mode (Amplitude Modulation Tapping Mode) of the AFM, while images c and d were taken using the Qi (Quantitative Imaging Mode) measurement mode of the AFM, and SWCNTs (2 nm) are spin-coated onto the mica surface and then washed with water.*

The length distribution is different for the different surfactants, with mean values between 600 nm and 800 nm. We want to note that the length distribution could impact the obtained data. However, this does not affect the overall conclusions because the effects would rather reduce/diminish the observed effects but not exaggerate them:

a) In the analysis of the impact of the organic corona (Figure 3b) on shielding of quantum friction, we normalize to the diffusion constant at zero exciton concentration (D_0). The reason

for that is that both the length distribution and the hydrodynamic radius could be different for different surfactants/samples. We thus focused on the relative change in diffusion.

b) Longer SWCNTs are known to be brighter than shorter ones. Given that fluorescence quantum yields of fluorophores such as SWCNTs are very low, it could be that increasing laser intensity in FCS experiments means that one starts to ‘see’ shorter SWCNTs. This would mean faster diffusion. We observe the opposite effect (slowing down).

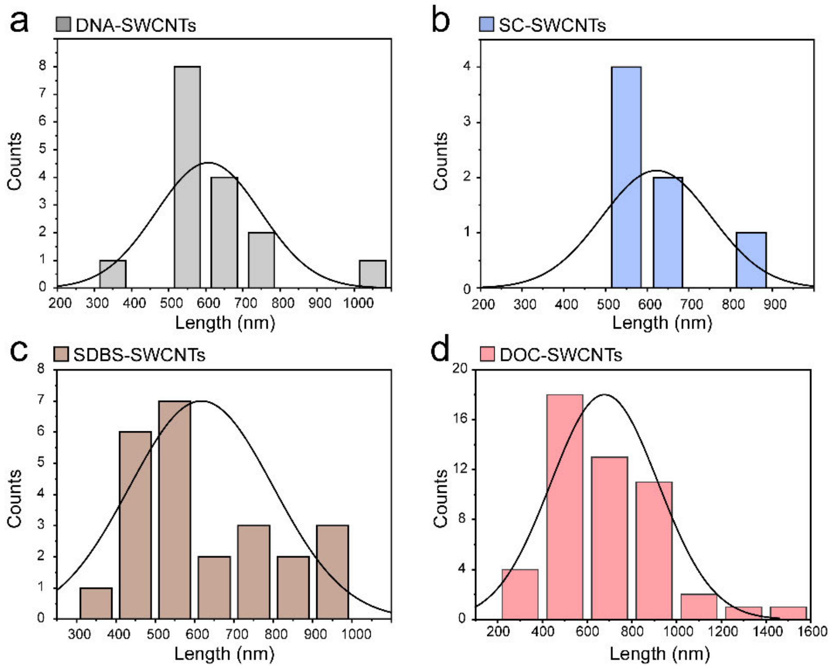

Figure R15 (S7). Length distribution of SWCNTs measured by AFM*. a) $(GT)_{10}$-SWCNTs (mean length 607 ± 140 nm), b) SC-SWCNTs (mean length 622 ± 131 nm), c) SDBS-SWCNTs (mean length 617 ± 180 nm), d) DOC-SWCNTs (mean length 677 ± 240 nm). The blank solid lines indicate Gaussian fits that were used to extract the mean ± SE for each distribution. ~ 30 particles are measured for each sample. Length was determined manually in Image J software.*

In summary, the used samples have the expected length distribution and did not show signs of bundling, aggregation etc. So far, SWCNT samples always have a length distribution, but in the future, one could use or work on length purification protocols to solve this issue and understand the impact of length in detail.

2) “The manuscript talks about different density from the various surfactants used to wrap the CNTs. Are there any data supporting these assessments?”

Response: There is experimental evidence in the literature that different surfactants give distinct buoyant densities to SWCNTs, which allows for their separation through

ultracentrifugation.[20,21] Experimental studies have measured the number of surfactant molecules per nanometer for various chiralities, demonstrating that sodium cholate and sodium dodecyl sulphate (SDS) result in different levels of coverage and consequently different densities.[20,22] The surfactant composition changes the assembly's density, which has implications for separation protocols.[22] As explained in answer 1, different surfactants lead to a different corona, which affects the hydrodynamic radius and thus absolute diffusion constants. We therefore focused on the relative change of diffusion (Figure 3b) and normalized the data.

3) “(6,5) and (6,4) CNTs are both semiconducting species, so the results presented in the manuscript that argue that CNT chirality does not affect the observed effects are not such a great test. Using a metallic CNT sample would have been much more revealing.”

Response: Metallic SWCNTs can unfortunately not be used in our experimental approach because they do not fluoresce (no excitons), which simply means we cannot observe them in a microscope. Thus, our observation is limited to semiconducting SWCNTs that fluoresce. Given that the simulations show the importance of the dynamic charge fluctuations by the excitons, we also believe that metallic SWCNTs would not show the same (light-induced) effect. We agree that (6,4) and (6,5) SWCNTs have similar diameters. For those two SWCNT chiralities, we did not find strong differences, but one cannot rule out that for larger diameters they exist. The reason for choosing these SWCNT chiralities is the limited sensitivity of the detectors in our FCS setup in the NIR region > 1000 nm, corresponding to larger diameter SWCNTs. Thus, we are at the moment experimentally limited to these chiralities (that emit around or below 1000 nm). We refined this part of the text.

4) “Why was Figure S7b normalized to the diffusion constant at 10uW power? Should not it be normalized to D_0 (i.e. extrapolated value at zero power)?”

Response: Thank you for pointing this out. We agree that normalizing to the extrapolated diffusion constant at zero power (D_0) is more appropriate for assessing power dependent trends. We have corrected the figure, and the updated figure (Figure S15b in the revised manuscript) is included in the supplementary information.

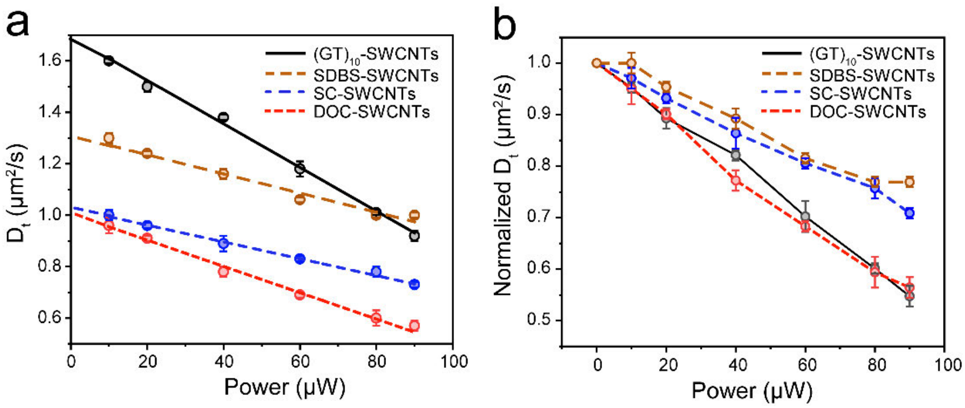

Figure S15**. **Impact of surface chemistry/corona shielding on diffusion. *a) Diffusion constants as a function of excitation power, the diffusion constants were fitted to a linear equation (Table ST14). b) Normalized diffusion constants as a function of power for $(GT)_{10}$-SWCNTs (black), SDBS-SWCNTs (brown), SC-SWCNTs (blue), and DOC-SWCNTs (red) (n=3, mean ± SD).*

5) “The results on chemical modification of the observed effects feel less impressive. Did the author check that ascorbic acid and riboflavin did not change association/dissociation of CNTs? For example, what would dynamic light scattering measurements show?”

Response: The use of SWCNTs for molecular sensing is an expanding field.[23] In general, this type of sensing is not based on the association or dissociation of CNTs, as evidenced by single nanosensor experiments by us and others.[24–26] Indeed, we have shown that single immobilized nanosensors respond to single analytes such as ascorbic acid.[25] We want to emphasize that this work is not about sensing. We just use our knowledge that DNA modified SWCNTs emit more (ascorbic acid) or less (riboflavine) fluorescence in the presence of these analytes. This fluorescence intensity is directly related to the exciton concentration (Figure 2), which we suggest is proportional to quantum friction. We believe that chemical manipulation adds further evidence to this model and serves as a complementary control.

We additionally checked the concern of association/dissociation by collecting absorption spectra, which are sensitive to aggregation. If there is something like association/dissociation happening, one would expect a shift in the spectrum.[27] There were no characteristic peak changes (Figure R16/(S13)), indicating that the SWCNT dispersion remained stable and that there was no significant aggregation or dissociation induced by the analytes. We agree that DLS measurements could be used, but at the very low (nM) concentrations we use in our experiments, DLS would be difficult. Overall, the absorption spectra prove the absence of aggregation effects.

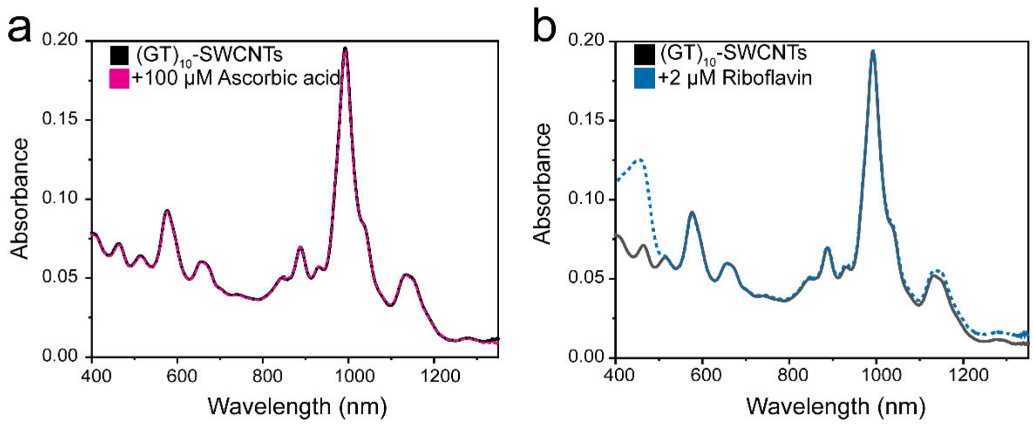

Figure R16 (S13). Absorbance spectra of (GT)$_{10}$-SWCNTs in response to ascorbic acid and riboflavin*. The absorbance of (GT)$_{10}$-SWCNTs demonstrates no change when a) ascorbic acid (100 µM) or b) riboflavin (2 µM) is added. Note that riboflavin has a colour and therefore the spectrum in b with riboflavin contains an additional peak in the blue region. The relevant feature of the (6,5)-SWCNTs at around 1000 nm is not affected by the analyte, which shows that the analytes do not induce aggregation. They only change the fluorescence quantum yield (Figure 2a).*

6) "On the same note, small compounds can affect hydrogen bonding in water (i.e. exhibit chaotropic or kosmotropic behavior). Those changes can plausibly affect the diffusion dynamics significantly even in absence of any quantum friction effects."

Response: Thank you for highlighting the important role of chaotropic and kosmotropic effects that small compounds have on water's hydrogen-bonding network. The concentrations of the compounds are relatively small (100 µM and 2 µM). At these concentrations, it is less likely that these effects play a significant role.

There are further lines of support for why we are convinced that the chemical manipulation directly affects quantum friction:

- The effect of the two analytes is inverse (Figure 2b) as expected from the inverse effect on fluorescence intensity. Additionally, it is concentration dependent and linear, as expected from a direct dependence on exciton concentration (Figure 2b, Figure S10a).
- We had presented in figure S12 that glycerol addition leads to a breakdown of the quantum friction power dependence. However, for up to 20 % volume percent, the effect is still observable, which suggests that such high concentrations would also be necessary for chaotropic/kosmotropic effects and our concentrations are much lower.
- A chaotropic or kosmotropic effect will most likely affect the effective hydrodynamic diameter. The diffusion constant of the very long (600 nm) and very thin (< 1 nm) SWCNTs is mainly dominated by the length, which should not be affected much.

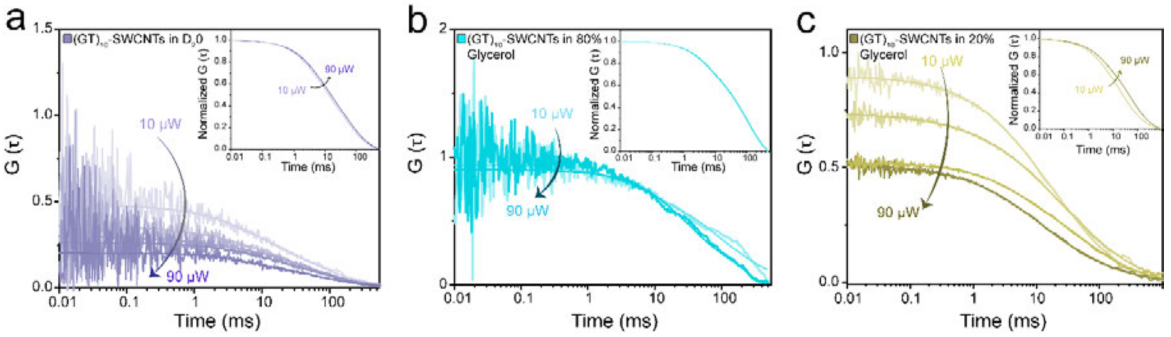

***Figure S12. Solvent effects on quantum friction**. Excitation power-dependent change (10 µW – 90 µW) of fluorescence autocorrelation functions of SWCNTs in different solvents. a) $(GT)_{10}$-SWCNTs in D_2O-based PBS (Table ST3), b) $(GT)_{10}$-SWCNTs in 80% glycerol in water (Table ST4), b) $(GT)_{10}$-SWCNTs in 20% glycerol in water.*

In summary, the results and these arguments support that chaotropic/kosmotropic effects should play a minor role in the chemical manipulation of quantum friction in our system.

7) “The most puzzling component of the manuscript is PS bead experiment, which frankly feels like it throws some sand in the otherwise interesting and well-constructed story. Does this experiment mean that all microscale object exhibit quantum friction? Do the PS beads exhibit the same exciton dynamics? If so, why? What about the drastic differences in size, geometry, or materials properties between CNTs and PS beads? What is the actual mechanism of ascorbic acid effect on the mobility of a relatively large particle?”

Response: Please also see response 5 to reviewer 1. The basic idea of this experiment was that if SWCNTs experience more friction, it could become more difficult for a larger object to move through a solution of such SWCNTs (= increase in viscosity). As we have shown in figure 2, QF (quantum friction) increases when ascorbic acid is added because it increases the fluorescence (= exciton concentration) in SWCNTs.

The movement of larger objects such as these beads is then affected by viscosity, which we observed in figure S27. Obviously, the connection between the viscosity of an (ensemble) SWCNT solution and QF is more complex than for a single SWCNT. It resembles indeed the question of how a solution of a semiflexible polymer (such as SWCNTs) changes its viscoelastic properties, which is a non-trivial problem in active matter physics. As the reviewer pointed out, the beads are indeed not expected to experience any quantum friction. We did these experiments in the first place because it was difficult to perform single SWCNT tracking. SWCNTs diffuse very quickly, and thus the tracks were very short. This was the initial consideration to image larger fluorescence labelled beads in a SWCNT solution instead of SWCNTs directly. However, tracking of SWCNTs is definitely a more direct way.

Consequently, we worked on tracking single SWCNTs in a wide field microscopy setup in the past months, but it was difficult for the following reasons:

- SWCNTs behave like single molecules and diffuse very quickly out of focus (z-direction), and thus tracks were short. There are elegant ways to extend the z resolution[28] or actively change the focus, but it is not a technique available to us at the moment.

- One can slow down diffusion by increasing viscosity by adding other molecules to the aqueous solution. However, adding glycerol reduces (20 % glycerol) or completely abolishes (80 %) the quantum friction effect we observed in the FCS experiments (Figure S12). It makes sense given that the water network, as well as the THz spectra, are affected by such compounds.[29] Therefore, these often used methods cannot be used in our case.

In the end, we finally found an experimental setting that works for us by using very thin flow chambers (Mylar spacer with a thickness of 2.3 micrometers) that reduce the defocusing in the z dimension (see Figure R17 (S28, S29), Figure R2/Figure 4d). This way, we could track single SWCNTs and study the impact of light excitation in a wide field setup (see also reviewer 1, question 5), which is complementary to the FCS experiments. We report the mean square displacements (MSDs) in the new Figure R2/Figure 4d (reviewer 1, response 2). This data set is a more direct way of showing a light intensity effect on SWCNT diffusion. We have now moved the bead experiments into the supplementary information. While we believe that they are very interesting, we realized that their interpretation is more complex and a topic for future studies.

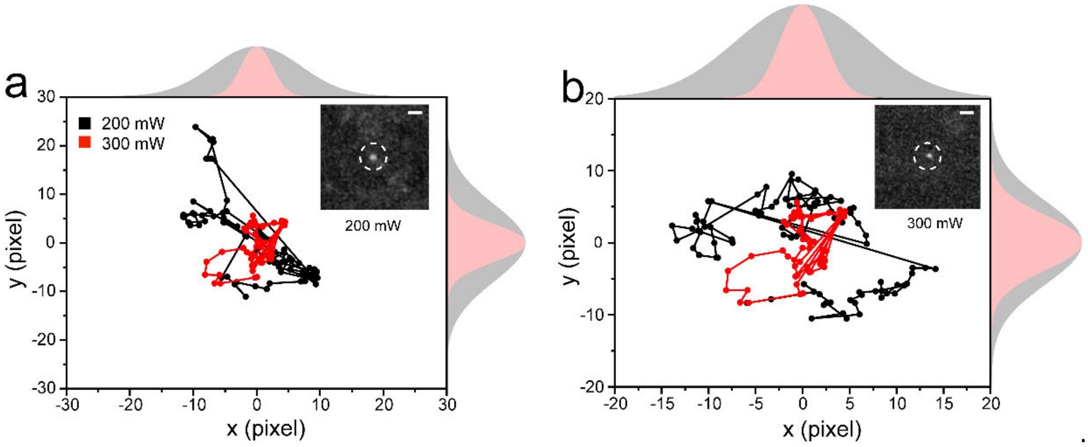

Figure R17 (S28). Wide field single SWCNT tracking. *a,b) Exemplary single-particle trajectories of DOC-SWCNTs diffusing in an aqueous solution with 200 and 300 mW laser excitation in a wide field microscopy setup. Gaussian functions are used to fit the position histograms (Scale bar: 1 µm). The inset shows the image of the single SWCNT (dashed circle).*

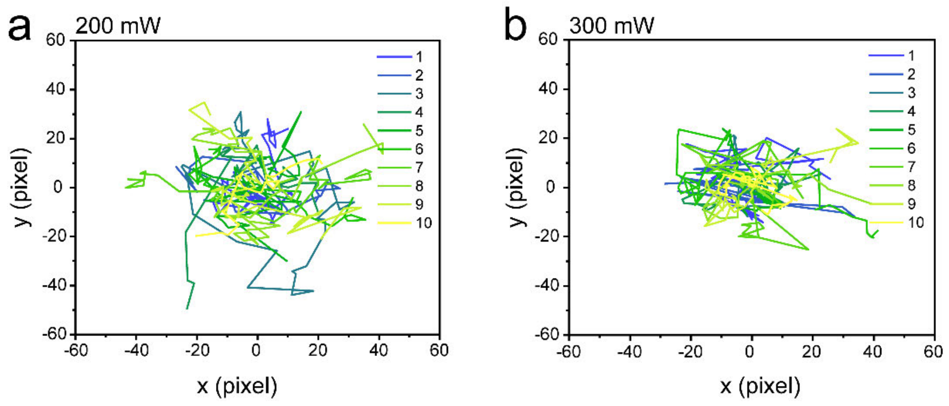

Figure R18 (S29). Particle trajectories: *Single-particle trajectories of DOC-SWCNTs diffusing in aqueous solution under (a) 200 mW and (b) 300 mW laser power are shown. The labels 1 to 10 indicate individual particle identifiers.*

Longer SWCNTs produced by the HiPCO process were also utilized. These samples allowed for imaging SWCNTs with lengths of several micrometers (Figure R19 (S30). In contrast, short SWCNTs rotate rapidly, resulting in rotational motion that is likely averaged during imaging. As a result, the measured diffusion constants for short SWCNTs primarily reflect translational motion. However, for longer SWCNTs, rotational motion is not averaged out, and both rotational and translational contributions are present more easily accessible. With this supplementary figure we want to communicate that it is possible to study long SWCNTs in which one can resolve their axis (length above the Abbe limit). It is highly interesting but a topic for future studies because the different axis, as well as the undulation of the whole SWCNT (as a semiflexible polymer), makes analysis and conclusions more complex.

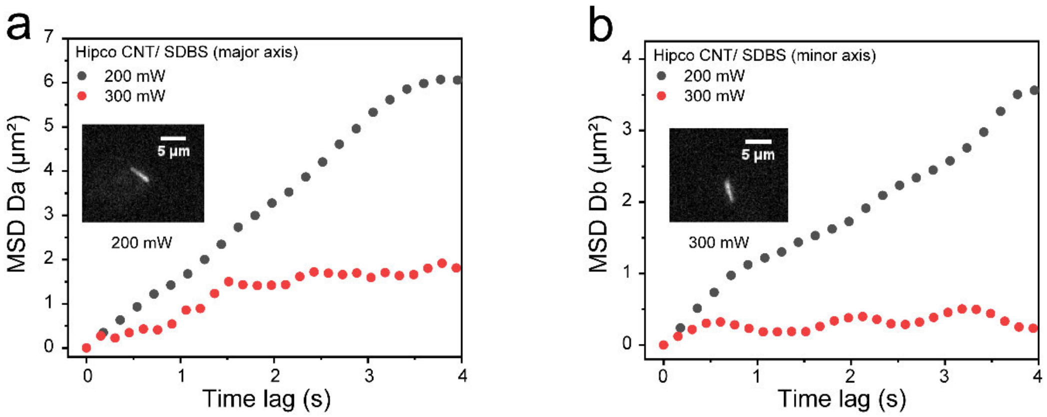

Figure R19 (S30). Anisotropic diffusion of HiPCO SWCNTs under varying excitation power*. a) Time average mean square displacement (TA-MSDs) of a 4.4 µm HiPco SWCNT in 0.5 % SDBS under linearly polarized excitation at 200 mW and 300 mW. a) TA-MSDs along major axis and b) TA-MSDs along minor axis diffusion. The insets show images of the SWCNTs. The center of mass was identified by fitting an ellipsoid to the SWCNT. Undulations of the SWCNT also affect the analysis of the trajectories.*

8) “I cannot help, but feel that more insights could be gained from MD simulations, which feel almost like a missed opportunity in the way the results are presented. The MD simulations section is painfully short and misses all the possible atomistic level insights that could potentially be gained from these studies.”

Response: Following the reviewer’s suggestion, we have expanded the analysis in the simulation results to gain more insights. See also responses to reviewer 1. The following points have been added:

- Analysis of the fluctuations related to the exciton dipole moment: the dynamical nature of the exciton is a key to explaining the exciton-water coupling. The exciton dipole fluctuations are reported in Figure R1b (4b)). The electron-hole excitation can be created both in the polarizable and non polarizable models; however, when the exciton is created in the non polarizable model, the charge remains more localized (distributed over the carbon atoms only), and the total dipole moment associated to the excitation is “static” with fluctuations (calculated from the standard deviation σ) in the order of 0.009 Åe. Instead, when the polarizable model is excited, the dipole fluctuations are much larger, namely σ = 0.45 Åe, namely 50 times larger.
- Analysis of the frequency spectrum of the cross correlation between the exciton dipole and water dipole. In figure R2/(S24), the FT spectra of the cross correlation between the dipole moment of CNT (exciton) and water are reported. Such spectra show large contributions in the frequency range below 1000 $cm^{-1,}$ suggesting a coupling to the collective modes of water. We would like to stress that the dynamical nature of the dipole is a key element to explain the observed slow down. To best illustrate this a “dynamical” exciton model (obtained using a polarizable model for the SWCNT) is compared to a “static” model where a dipole of the same size, is added to the system using charges which are localized to the carbon atoms and not allowed to fluctuate (this obtained with a non polarizable model of the SWCNT, here dipole fluctuations are reduced by three orders of magnitude). The “static dipole” minimally couples to water collective modes (no contributions to the cross correlation is visible in the spectra in figure R2/(S24) on the same scale as the polarizable model).
- Comparison of the friction coefficient with polarizable (“dynamical” dipole) and non polarizable model (non-dynamical dipole): In the case of the polarizable model (“dynamical” exciton), the friction increases by about a factor of 3 with respect to the non polarizable (static dipole) due to the exciton-water coupling (Figure R13/Figure S26). In the first case, where a “dynamical” dipole is present, the friction is the result of a quantum phenomenon, although classically described by the simulation model,

namely the coupling of the charge fluctuations to the water low frequency modes. In the second case ("static" dipole), only dielectric friction is generated as a result of the electrostatic interaction between the "static" dipole (less dynamic) and the water charges. Additionally, we now modelled the role of phonons by comparing the cross correlation with water dipoles when vibrational coupling is possible or not (Figure R3(S25)).

9) "Finally, some additional comments on the manuscript structure. First the introduction feels meandering. It talks about using CNTs for biosensing, which is highly irrelevant to the topic of the manuscript. I suggest streamlining it. One the same token, the topic of quantum friction, which is central to this work, is not addressed in any depth. "Energy and momentum transfer to a surrounding solvent" can easily describe standard non-radiative relaxation, so a description that pays more attention to the physics of quantum friction will be very welcome. Finally, as I mentioned before, MD simulations are described in a very cursory way, even though they provide an important validation and perhaps the only way to see what is happening in this system at the atomic level."

Response: In the revised version, we followed these comments and suggestions and

a) We streamlined in the introduction the discussion of SWCNTs as biosensors. We still believe that mentioning this topic is important because the fluorescence changes to analytes (=biosensing) is used for chemical manipulation of quantum friction in figure 2.
b) We extended the discussion of quantum friction.
c) We added novel simulation data and embedded the simulations (polarizable versus non-polarizable models) much better into the whole story.

Referee 3:

1) "All the experiments were well done, and the details of methods were sufficiently described in the text. The data and results were well presented with appropriate descriptions of errors except for Figures S7 and S9".

Response: Thank you for the positive assessment. We added the description of the error bars in those supplementary figures.

2) "This paper provides an interesting concept of quantum friction on one-dimensional materials induced by exciton-water coupling, which is the outstanding feature of the work and should be of significance and broad interest in the field of physics and chemistry on

nanomaterials…The concept of exciton-water coupling induced friction is new and should be immediately recognized, examined and used by the researchers in the field. Thus, I think the paper potentially deserves publication. However, before publication, I recommend the revision concerning the following points to strengthen the paper."

Response: We thank the reviewer for the positive assessment of our work and highlighting the novelty.

3) "In the second paragraph, the authors rejected the possibility of sample heating by a control experiment using the dye Atto 488. Is the fluorescence quantum yield of this dye similar to that of SWCNT? If the quantum efficiency of the dye is much higher than that of SWCNT, the laser-induced heating of dye molecules and surrounding water molecules is low. The relaxation by fluorescence does not rise temperature."

Response: Thank you for your thoughtful comment. Atto 488 has a significantly higher fluorescence quantum yield compared to SWCNTs. As a result, we would expect its non-radiative relaxation pathways and associated local heating to be lower. To investigate this concern, we conducted direct temperature measurements on $(GT)_{10}$-SWCNTs under both low and high excitation power conditions (Figure R8/(S11)). See also answer 5 to reviewer 1

Our measurements confirmed that the sample temperature remained stable across the entire power range used in our experiments. We believe these complementary temperature measurements provide strong evidence that sample heating does not significantly influence our observations. It does not rule out local heating, for example, only in the confocal volume, which is difficult to measure. However, as discussed above, such effects would only reduce the effect of quantum friction but not exaggerate it. Local heating could cause convection or a decrease in water viscosity that would both increase the movement/diffusion of SWCNTs, but not the (observed) opposite decrease in diffusion.

The Atto 488 control experiment was conducted primarily to determine whether changes in excitation intensity affect the confocal volume or cause sample heating. The results indicated that diffusion remained constant across all power levels, thereby excluding these potential issues.

4) "I guess that the Coulomb interaction between a dipole of water molecule and polarization of trapped/localized exciton on diffusive (moving) SWCNT could affect friction. It

is helpful to make a physical explanation why the trapped/localized exciton does not affect friction. This comment is related to the comment 5 below."

Response: We agree that Coulomb interactions between water dipoles and localized excitons on SWCNTs influences friction. Excitons in normal SWCNTs are highly mobile and diffuse on the order of 100 nm during their lifetime.[16] This mobility allows excitons to interact dynamically with their environment, potentially perturbing nearby water molecules and contributing to frictional effects through continuous dipole moment fluctuations.

A localized exciton is confined to the quantum defect/local SWCNT region, which is on the order of 1 nm in size. The physical picture would be that the exciton behaves more like a static dipole rather than a moving one. Although a static dipole can still interact electrostatically with surrounding water molecules, it would not induce the dynamic polarization changes in the solvent that a moving dipole would.

In summary, while trapped/localized excitons can interact with water dipoles through Coulomb forces, their lack of mobility prevents them from inducing the dynamic solvent reorganization needed to affect friction. Therefore, mobile excitons, capable of diffusing along the SWCNT, can significantly enhance friction more through their dynamic interactions with the surrounding medium. The newly added simulations of a polarizable versus non polarizable excitons support this physical picture (Figure R3/(S25)). See also answer 3 to reviewer 1. We added this physical picture to the text.

5) "The dielectric constant of surfactants could affect the interaction between exciton and water molecule (screening effect). Is it possible to discuss the screen effect of surfactants used in this study?"

Response: Yes, we completely agree. It is known that the dielectric environment surrounding SWCNTs affects the exciton. Reduced surface coverage and more exposure to water affects exciton screening, resulting in more pronounced redshifts in the spectra[30]. The used surfactants create differently dense layers on the SWCNT surface,[31] which affects the screening of excitons.

In Figure 3b and Figure S14, we studied exactly this screening effect. The impact on the diffusion constant/quantum friction is higher if the spectra are more red-shifted (= "see" more water to exchange energy/momentum via quantum friction). Thus, the corona around the SWCNTs shields quantum friction, and its chemical composition and structure determine the magnitude. Even though we see this correlation (Figure 3b), one could, in the future, perform

MD simulations that focus on the water structure and local dielectric properties for these different surfactants and coatings to fully understand the nature of the correlation.

6) “In the optical-pump THz-probe measurements (Figures 3c and 3d), the transient signal lasts at least 300 ps. Were the excitons long-lived in this time scale? It is helpful to show fluorescence lifetime of excitons.”

Response: Thank you for your thoughtful question and for highlighting an important aspect of our OPTP measurements. Indeed, in figure 3d, the transient THz signal persists for at least 300 ps, which raises the question of whether this indicates the presence of long-lived exciton populations. The THz signal in our measurements primarily probes either photoinduced conductivity or the solvent response. In the case of SWCNTs, excitons typically have very short radiative lifetimes, ranging from tens to hundreds of picoseconds.[32]

The fluorescence lifetimes of such samples have been measured by us before and are on the order of 100 ps and we refer to this work in the manuscript.[33] We used the same setup before to measure the slow lifetime component (370 ps). Note that the shorter and more prominent lifetime component was too fast for our optical setup. Nevertheless, we believe that the complex exciton dynamics and dark excitons could make photophysics more complex, which could be studied in the future.

In summary, the slow fluorescence lifetime component is on the same order of magnitude as the slow decay time scales of the THz spectra. We interpret it in such a way that the energy and momentum dissipate from the SWCNT through the exciton water interaction into the water THz modes, and thus, the time scales are similar. We have clarified this connection in the manuscript.

7) “The Drude-like oscillators are suitable for describing classical plasmons, but not for an electron and hole consisting of exciton, because the exciton is a bound state of them. Which modes (excitations) in exciton are used to couple the collective translational modes of water molecules?”

Response: The modified charge on the Drude-like oscillators describes here the delocalized negative charge (electron) and the delocalized positive charge (hole) in a bound state. Here, the exciton is not allowed to diffuse along the SWCNT axis or to annihilate with another exciton. Namely, it can be thought to be in a stationary state in analogy with the experimental conditions. The electron and the hole will maintain a constant average distance. However, the

exciton dipole will have a dynamical nature with fluctuations in the order of 0.45 Åe. These fluctuations of the exciton dipole moment couple to the water modes. Such a coupling is visible in the frequency spectrum of the cross-correlation between the exciton dipole and water dipole, which is reported in figure R2 of the reply and figure S24 of the revised Supplementary Material. In the polarizable model (where the Drude particles are present), coupling between the excitons mode and water is clearly visible in the range <1000 cm^{-1}, which also corresponds to the delocalized water modes, while in the case of the non polarizable (or of "static" dipole) such coupling is negligible and not visible on the same scale (Figure R2, zoomed in view displayed in Figure R2d-f and Figure R3d-f).

References

(1) Kavokine, N.; Bocquet, M.-L.; Bocquet, L. Fluctuation-Induced Quantum Friction in Nanoscale Water Flows. *Nature* **2022**, *602* (7895), 84–90.

(2) Bui, A. T.; Thiemann, F. L.; Michaelides, A.; Cox, S. J. Classical Quantum Friction at Water–Carbon Interfaces. *Nano Lett.* **2023**, *23* (2), 580–587.

(3) Nalige, S. S.; Galonska, P.; Kelich, P.; Sistemich, L.; Herrmann, C.; Vukovic, L.; Kruss, S.; Havenith, M. Fluorescence Changes in Carbon Nanotube Sensors Correlate with THz Absorption of Hydration. *Nat. Commun.* **2024**, *15* (1), 6770.

(4) Ma, M.; Grey, F.; Shen, L.; Urbakh, M.; Wu, S.; Liu, J. Z.; Liu, Y.; Zheng, Q. Water Transport inside Carbon Nanotubes Mediated by Phonon-Induced Oscillating Friction. *Nat. Nanotechnol.* **2015**, *10* (8), 692–695.

(5) Marbach, S.; Dean, D. S.; Bocquet, L. Transport and Dispersion across Wiggling Nanopores. *Nat. Phys.* **2018**, *14* (11), 1108–1113.

(6) Yu, X.; Principi, A.; Tielrooij, K.-J.; Bonn, M.; Kavokine, N. Electron Cooling in Graphene Enhanced by Plasmon–Hydron Resonance. *Nat. Nanotechnol.* **2023**, *18* (8), 898–904.

(7) Coquinot, B.; Bocquet, L.; Kavokine, N. Quantum Feedback at the Solid-Liquid Interface: Flow-Induced Electronic Current and Its Negative Contribution to Friction. *Phys. Rev. X* **2023**, *13* (1), 011019.

(8) Conti Nibali, V.; Pezzotti, S.; Sebastiani, F.; Galimberti, D. R.; Schwaab, G.; Heyden, M.; Gaigeot, M. P.; Havenith, M. Wrapping up Hydrophobic Hydration: Locality Matters. *J. Phys. Chem. Lett.* **2020**, *11* (12), 4809–4816.

(9) Schienbein, P.; Schwaab, G.; Forbert, H.; Havenith, M.; Marx, D. Correlations in the Solute-Solvent Dynamics Reach Beyond the First Hydration Shell of Ions. *J. Phys. Chem. Lett.* **2017**, *8* (11), 2373–2380.

(10) Pezzotti, S.; Sebastiani, F.; van Dam, E. P.; Ramos, S.; Conti Nibali, V.; Schwaab, G.; Havenith, M. Spectroscopic Fingerprints of Cavity Formation and Solute Insertion as a Measure of Hydration Entropic Loss and Enthalpic Gain. *Angew. Chem. Int. Ed. Engl.* **2022**, *61* (29), e202203893.

(11) Sneh, T.; Corsetti, S.; Notaros, M.; Kikkeri, K.; Voldman, J.; Notaros, J. Optical Tweezing of Microparticles and Cells Using Silicon-Photonics-Based Optical Phased

Arrays. *Nat. Commun.* **2024**, *15* (1), 8493.

(12) Thongam, D. D.; Chaturvedi, H. Functionalization of Pristine, Metallic, and Semiconducting-SWCNTs by ZnO for Efficient Charge Carrier Transfer: Analysis through Critical Coagulation Concentration. *ACS Omega* **2022**, *7* (17), 14784–14796.

(13) Nair, N.; Usrey, M. L.; Kim, W. J.; Braatz, R. D.; Strano, M. S. Estimation of the (n,m) Concentration Distribution of Single-Walled Carbon Nanotubes from Photoabsorption Spectra. *Anal. Chem.* **2006**, *78* (22), 7689–7696.

(14) Lamb, D. C.; Schenk, A.; Rocker, C.; Scalfi-Happ, C.; Nienhaus, G. U. Sensitivity Enhancement in Fluorescence Correlation Spectroscopy of Multiple Species Using Time-Gated Detection. *Biophys. J.* **2000**, *79* (2), 1129–1138.

(15) Dertinger, T.; Pacheco, V.; Von Der Hocht, I.; Hartmann, R.; Gregor, I.; Enderlein, J. Two-Focus Fluorescence Correlation Spectroscopy: A New Tool for Accurate and Absolute Diffusion Measurements. *ChemPhysChem* **2007**, *8* (3), 433–443.

(16) Lüer, L.; Hoseinkhani, S.; Polli, D.; Crochet, J.; Hertel, T.; Lanzani, G. Size and Mobility of Excitons in (6, 5) Carbon Nanotubes. *Nat. Phys.* **2009**, *5* (1), 54–58.

(17) Berends, A. C.; Rabouw, F. T.; Spoor, F. C. M.; Bladt, E.; Grozema, F. C.; Houtepen, A. J.; Siebbeles, L. D. A.; De Mello Donegá, C. Radiative and Nonradiative Recombination in CuInS2 Nanocrystals and CuInS2-Based Core/Shell Nanocrystals. *J. Phys. Chem. Lett.* **2016**, *7* (17), 3503–3509.

(18) Nißler, R.; Kurth, L.; Li, H.; Spreinat, A.; Kuhlemann, I.; Flavel, B. S.; Kruss, S. Sensing with Chirality-Pure Near-Infrared Fluorescent Carbon Nanotubes. *Anal. Chem.* **2021**, *93* (16), 6446–6455.

(19) Nißler, R.; Mann, F. A.; Chaturvedi, P.; Horlebein, J.; Meyer, D.; Vuković, L.; Kruss, S. Quantification of the Number of Adsorbed DNA Molecules on Single-Walled Carbon Nanotubes. *J. Phys. Chem. C* **2019**, *123* (8), 4837–4847.

(20) O'Connell, M. J.; Bachilo, S. M.; Huffman, C. B.; Moore, V. C.; Strano, M. S.; Haroz, E. H.; Rialon, K. L.; Boul, P. J.; Noon, W. H.; Kittrell, C.; Ma, J.; Hauge, R. H.; Weisman, R. B.; Smalley, R. E. Band Gap Fluorescence from Individual Single-Walled Carbon Nanotubes. *Science* **2002**, *297* (5581), 593–596.

(21) Bachilo, S. M.; Strano, M. S.; Kittrell, C.; Hauge, R. H.; Smalley, R. E.; Weisman, R. B. Structure-Assigned Optical Spectra of Single-Walled Carbon Nanotubes. *Science (80-.).* **2002**, *298* (5602), 2361–2366.

(22) Nair, N.; Kim, W. J.; Braatz, R. D.; Strano, M. S. Dynamics of Surfactant-Suspended Single-Walled Carbon Nanotubes in a Centrifugal Field. *Langmuir* **2008**, *24* (5), 1790–1795.

(23) Ackermann, J.; Metternich, J. T.; Herbertz, S.; Kruss, S. Biosensing with Fluorescent Carbon Nanotubes. *Angew. Chem. Int. Ed. Engl.* **2022**, *61* (18), e202112372.

(24) Kruss, S.; Salem, D. P.; Vuković, L.; Lima, B.; Ende, E. Vander; Boyden, E. S.; Strano, M. S. High-Resolution Imaging of Cellular Dopamine Efflux Using a Fluorescent Nanosensor Array. *Proc. Natl. Acad. Sci. U. S. A.* **2017**, *114* (8), 1789–1794.

(25) Gretz, J.; Ma, C.; Hill, B.; Schrage, C. A.; Kruss, S. Kinetic Insights into Carbon Nanotube-Based Neurotransmitter Sensors by Single Molecule Experiments. September 9, 2025, pp 1–27.

(26) Jin, H.; Heller, D. A.; Kalbacova, M.; Kim, J. H.; Zhang, J.; Boghossian, A. A.; Maheshri, N.; Strano, M. S. Detection of Single-Molecule H2 O2 Signalling from Epidermal Growth

Factor Receptor Using Fluorescent Single-Walled Carbon Nanotubes. *Nat. Nanotechnol.* **2010**, *5* (4), 302–309.

(27) Koh, B.; Cheng, W. Mechanisms of Carbon Nanotube Aggregation and the Reversion of Carbon Nanotube Aggregates in Aqueous Medium. *Langmuir* **2014**, *30* (36), 10899–10909.

(28) Lee, A.; Cognet, L. Length Measurement of Single-Walled Carbon Nanotubes from Translational Diffusion and Intensity Fluctuations. *J. Appl. Phys.* **2020**, *128* (22), 224301.

(29) Das Mahanta, D.; Brown, D. R.; Pezzotti, S.; Han, S.; Schwaab, G.; Shell, M. S.; Havenith, M. Local Solvation Structures Govern the Mixing Thermodynamics of Glycerol–Water Solutions. *Chem. Sci.* **2023**, *14* (26), 7381–7392.

(30) Choi, J. H.; Strano, M. S. Solvatochromism in Single-Walled Carbon Nanotubes. *Appl. Phys. Lett.* **2007**, *90* (22), 223114.

(31) Fagan, J. A.; Zheng, M.; Rastogi, V.; Simpson, J. R.; Khripin, C. Y.; Silvera Batista, C. A.; Hight Walker, A. R. Analyzing Surfactant Structures on Length and Chirality Resolved (6,5) Single-Wall Carbon Nanotubes by Analytical Ultracentrifugation. *ACS Nano* **2013**, *7* (4), 3373–3387.

(32) Amori, A. R.; Hou, Z.; Krauss, T. D. Excitons in Single-Walled Carbon Nanotubes and Their Dynamics. *Annu. Rev. Phys. Chem.* **2018**, *69* (1), 81–99.

(33) Sistemich, L.; Galonska, P.; Stegemann, J.; Ackermann, J.; Kruss, S. Near-Infrared Fluorescence Lifetime Imaging of Biomolecules with Carbon Nanotubes. *Angew. Chem. Int. Ed. Engl.* **2023**, *62* (24), e202300682.

Referee 1

Questions by the reviewer are shown in blue. Figures are numbered and e.g. labelled as R1. If the figure also appears in the manuscript or the SI the numbering from the revised version is used e.g. Figure R1/S25. New figures and new text cited from the revised manuscript is highlighted in yellow.

1) ”The authors made significant revisions to the manuscript. In particular, they have carried out additional single nanotube tracking experiments that support their conclusions. “

Response: We thank the reviewer for appreciating our efforts to further strengthen our conclusions.

2) “However, in many places, the physical interpretations are still very fuzzy. Particularly, when it comes to interpreting OPTP measurements and MD simulations, it looks like conceptual misunderstandings lead the authors to draw conclusions that are not supported by the data.”

Response: In response to the thorough review, we revised the manuscript. We believe that the changes made in this latest version will help clarify and strengthen the overall message of our work. We identify a new form of friction (measured by changes in diffusion), which we link to the coupling of electronic excitations within the solid to charge fluctuations in water. In contrast to the systems investigated before theoretically by Kavokine et al. (Nature 2022) the (electronic) excitation is not a plasmonic excitation, as in graphite or in graphene but best represented as electron-hole pairs (excitons) in the carbon nanotubes. This is substantiated by solid experimental evidence (FCS and particle tracking experiments/Figure 1, optical and chemical manipulation of the effect/Figure 2), differences in the effect depending on the organic corona around the SWCNTs (Figure 3) and THz spectroscopy that shines light on the mechanism (Figure 3) as recognised by the three referees.
A full theoretical description for the presented experimental phenomenon is not yet available and beyond the scope of this current work. We want to point out that it would involve a full rigorous description of the coupling of a moving exciton with the solvent fluctuations, which is a challenge that still needs to be addressed. The simulations presented here (Figure 4) aim to provide a simple classical model for the phenomenon, in the spirit of the classical theoretical model for quantum friction introduced by Cox et al. (Nano Letters, 2023). In that model Drude particles were attached to the atoms to mimic the electron density. In a similar way, the excitons are modelled (classically) by us with a (dynamic) electron-hole charge distribution, that mimics electron-hole charge fluctuations. Based on this simulation we propose an

explanation/ mechanism for the experimentally observed effect: The charge fluctuations of the exciton couple to water and increase the water-SWCNT friction, which slows down the diffusion of the SWCNTs in water.

We believe our experiments point to a new phenomenon of quantum mechanical origin with implications for nanofluidics, active matter, sensing, nanorobotics, etc. We have improved the manuscript to distinguish better between experimental results, their interpretation and limitations of the classical simulation. For details we refer to the responses below.

3) “The main issue is summed up in the very first sentence of the abstract, which has barely changed since the last version, although I explicitly mentioned it in my previous report. The authors state: "Quantum friction describes direct transfer of energy and momentum between electronic states in a material and a surrounding solvent". Quantum friction refers specifically to the transfer of momentum between a liquid and a solid's electrons. This momentum transfer is not necessarily accompanied by a net energy transfer. A similar microscopic mechanism is involved in the direct energy transfer between an electronically excited solid and a surrounding liquid, which is referred to as near-field radiative heat transfer (NFRHT). However, the observation of NFRHT does not imply that quantum friction is the dominant mechanism for solid-liquid momentum transfer, nor does the observation of quantum friction imply that NFRHT is the dominant mechanism for solid-liquid heat transfer. Nevertheless, the authors use these two concepts interchangeably. “

Response: We agree with the reviewer that this difference should be distinguished better. We have corrected the abstract as follows:

“Quantum friction describes the direct transfer of momentum between a liquid and a solid’s electrons”.

As the reviewer states quantum friction does not necessarily imply energy transfer, although energy transfer can accompany momentum transfer. In general, we streamlined the flow of arguments in the manuscript. We first present experimental evidence for changes in diffusion of SWCNTs and which parameters change it (Figure 1-3). We then propose a mechanistic picture and the link to quantum friction, based on the THz experiments (Figure 3c,d) and the classical simulations (Figure 4). We respond on the relevance and potential contribution of NFRHT in the specific question below (question 4).

4) “For instance, they conclude: "On the 0.7 ps time scale a rapid response is observed. Similar to (hot electron cooling in) graphene, we attribute this fast decay to quantum friction between SWCNTs and water". If the decay can be attributed to anything it is to NFRHT, not quantum friction! Even if the spectra represent indeed the energy transfer pathways suggested by the authors, this does not support the interpretation of the diffusion slowdown in terms of quantum friction, unless a quantitative analysis of the timescales is made as in Ref. 7. At present, I feel that the THz measurements can be an interesting side note, but they cannot support the overall argument.”

Response: We agree that experimentally, both classical friction and quantum friction will be present and difficult to separate from NFRHT. We want to emphasize that the diffusion experiments are the experimental proof for a new kind of light/exciton related friction. We use THz spectroscopy and MD simulations to propose a mechanism that links it to quantum friction. In the following we want to explain why we see the THz measurements as very important spectroscopic support for our proposed mechanism.

Short response:

First, we compare our OPTP decay (in water) with literature values and find faster decay in water similar to the arguments in reference 7 (detailed response 4.1). This first and fastest process (< 1 ps) is interpreted as interaction with water during the relaxation of the exciton (after excitation at 400 nm) to its bandgap energy (around 1000 nm emission). However, given that we cannot use other solvents we do not want to overinterpret this feature. Next, we extended our THz analysis and present our main argument. We had found and described that optical excitation of SWCNTs increases THz absorption in general. Now, we have used the spectral resolution and dissect the spectrum into a thermal component and a special THz feature (below 50 cm^{-1}). This feature is unique both in terms of the wavenumber and time evolution and differs from the THz response of thermal heating (detailed response 4.2). It also fits the exciton decay lifetimes and thus we propose that this THz feature represents direct coupling of excitonic charge fluctuations in the SWCNT with water, providing a potential pathway for quantum friction. Additionally, we outline in greater detail (detailed response 4.3) why the chemical manipulation of diffusion (Figure 2), the chemical shielding (Figure 3b) and FCS diffusion measurements in D_2O (Figure R9/S12) do not align with a pure thermal/NFRHT pathway.

Detailed response:

4.1) In Reference 7 the time scale of electron cooling of graphene after optical excitation in different solvents was compared. Time scales were faster in water than in other solvents, which led the authors to conclude that quantum friction contributes to cooling of hot electrons:

"We are thus led to hypothesize, as anticipated above, that the liquid provides the electrons with a supplementary cooling pathway, which, in the case of water, has an efficiency comparable to the intrinsic cooling pathway via phonons. We then interpret the faster cooling as a signature of 'quantum' electron–liquid energy transfer."

Our samples are only water soluble, and therefore we cannot directly test other solvents. In solvents such as methanol/ethanol, all functionalized SWCNTs (DNA, SC, SDS) would precipitate. We thus compared previously reported THz time scales for SWCNTs in the absence of water with the time scales from our OPTP measurements of SWCNTs in water.

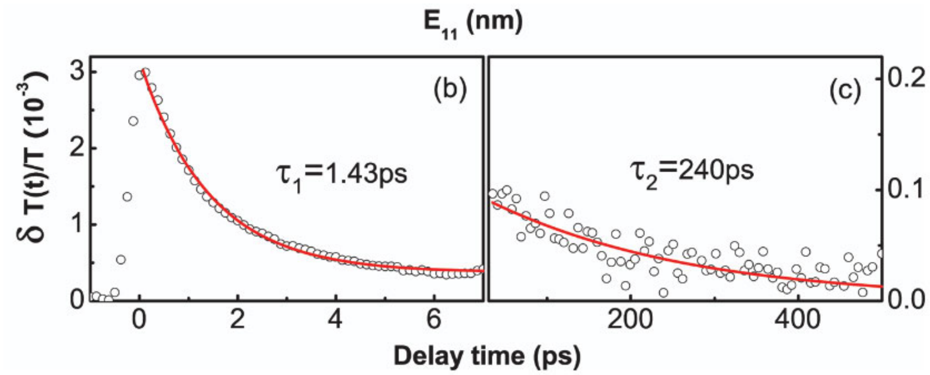

Figure R1: *Differential THz transmission (of a dried SWCNT-gelatin film) as a function of time after optical excitation. Two exponential decays were observed. From Xu et al. (Terahertz Excitonic Response of Isolated Single-Walled Carbon Nanotubes, J. Phys. Chem. C, Vol. 113, No. 42, 2009[1]).*

In Figure R1 the cooling time (upon photoexcitation at 400 nm) of SWCNTs in a different environment than water is shown (Xu et al. JPC C 2009[1]). They performed an OPTP experiment probing the change in conductivity (or amplitude of the electric THz probe field) upon photoexcitation at 400 nm at the same pump frequency we used. In their experiment, a SWCNT-gelatin solution was deposited on a substrate, desiccated in air, and then lifted off to provide free-standing SCWNT-gelatin films. They deduced two time scales for relaxation, and the fast time scale showed a lifetime of around 1.4 ps. Below (Figure R2), you find maximum

amplitudes of Δ mOD (optical density) of DOC-SWCNTs measured by us at short time delays using a free-standing water jet as solvent environment.

The extracted decay times were 0.7 ps (at a pump fluence of 120 or 200 μJ/cm^2) but in all cases shorter than the 1.43 ps as reported in the literature above. It shows that initial decay is faster in water compared to air, which is in line with the argument and the time constant in the mentioned paper (Reference 7, Yu et al. Nature Nano. 2023). We interpret this first fast (< 1 ps) process ("component 0") as interaction with water during the relaxation process (of the exciton) after photon absorption (400 nm) to the bandgap energy level (around 1000 nm). However, we agree that it only indicates energy dissipation and the matching time scales with Reference 7 could be a coincidence.

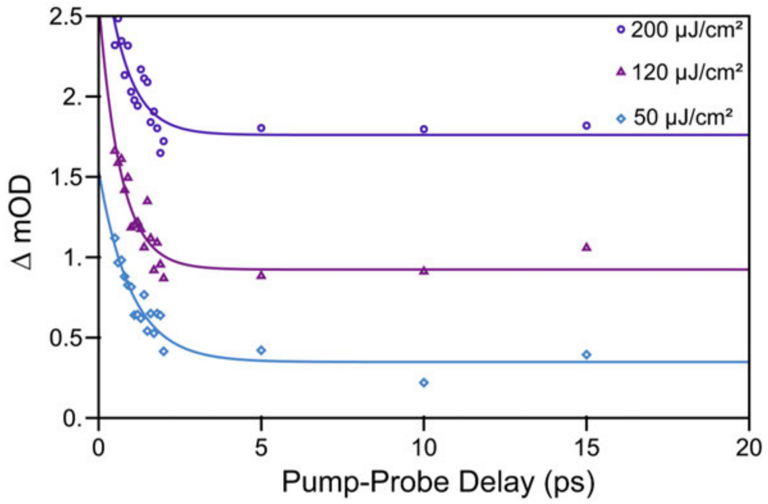

Figure R2/S26: Pump–probe optical density changes: *Change of optical density of DOC-SWCNTs at a frequency of 26 cm^{-1} as a function of pump-probe delays. Plotted are the results for three distinct optical pump powers. The amplitudes were fitted (solid line) to the exponential function ΔmOD = c + A exp(-t/τ). The extracted decay times are τ = 1.0 ps for a flux of 50 μJ/cm^2 and 0.7 ps for a flux of either 120 or 200 μJ/cm^2.*

4.2) We agree that the interpretation of the THz spectra could be very informative. In the last month we have developed a better understanding and provide a physics-based interpretation in the main text. We want to point out that in our experimental set-up we can map the phase and the amplitude at each delay position and thereby retrieve the change in optical density as a function of frequency at each time delay, i.e. we obtain the full spectral THz response as a function of time after optical excitation. We found that the observed THz spectrum (on time scales > 1 ps) can be dissected into two additional distinct spectral

responses: An initial response centred in the frequency range below 50 cm^{-1} and a subsequent response > 100 cm^{-1}, which increases with time and becomes dominant for pump probe delays above 200 ps (Figure R3). These two partial contributions can be distinguished by their different spectral responses, dependence on pump probe time delays.

The referee pointed out that the changes in the THz spectra could be due to energy transfer to the water via NFRHT. Therefore, we have also plotted (Figure R3) for comparison the spectral response as expected in case of heating of bulk water by 0.11K (black line). Heating implies excitation of translational modes, which are centred around or above 80-100 cm^{-1}. Only at larger time delays, i.e. at a few hundred ps the change in optical density resembles the spectral changes as expected for heating.

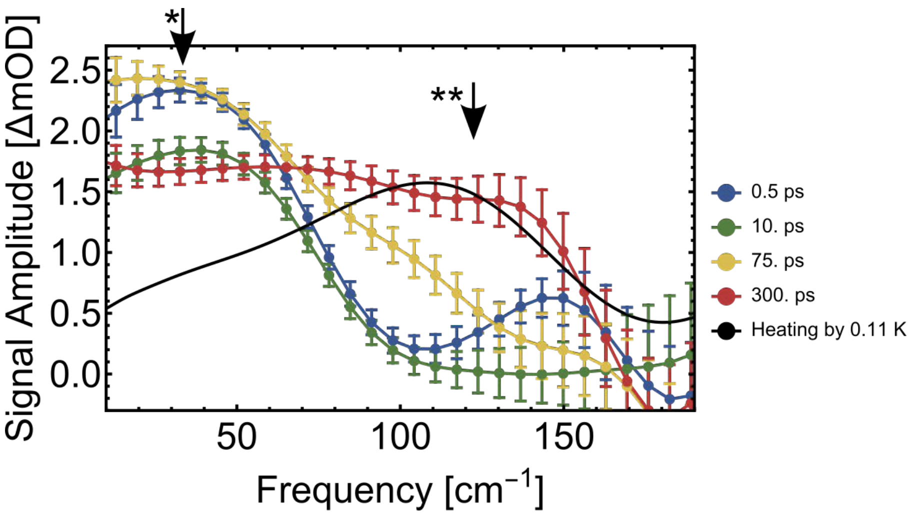

Figure R3/S29: Optical Pump THz Probe (OPTP) spectroscopy of SWCNTs in water*. Plotted is the difference in THz absorption of an aqueous DOC-SWCNT solution as a function of time delay after optical excitation. One can compare the changes to the changes when heating a sample of bulk water e.g. by 0.11 K (from 10°C to 10.11°C, black line and modelled based on experimental steady-state spectra reported before[2,3]). The two arrows indicate the features that we interpret as exciton-water coupling (*) and thermal heating (**) (mean ± SE, n=22).*

We want to point out that the spectral OPTP response of SWCNTs differs significantly from the spectral response of other dyes such as 8-Hydroxypyrene-1,3,6-trisulfonic acid (HPTS) (Figure R4a,b) or [Tris(bipyridine)ruthenium(II) chloride] (Ru(bpy) (Figure R4c, unpublished data). In both cases the spectral response resembles heating but there is no distinct feature below 50 cm^{-1}.

Thus, there is a new feature below 50 cm^{-1} that is not present in other fluorophores/dyes and decays on the same time scale as excitons decay in SWCNTs (10 ps – 100 ps)[4–6]. The 300 ps spectral response resembles that of water experiencing a temperature increase of water by around 0.1 K. On this time scale most excitons will have already recombined or dissipated their energy as heat into phonon modes.

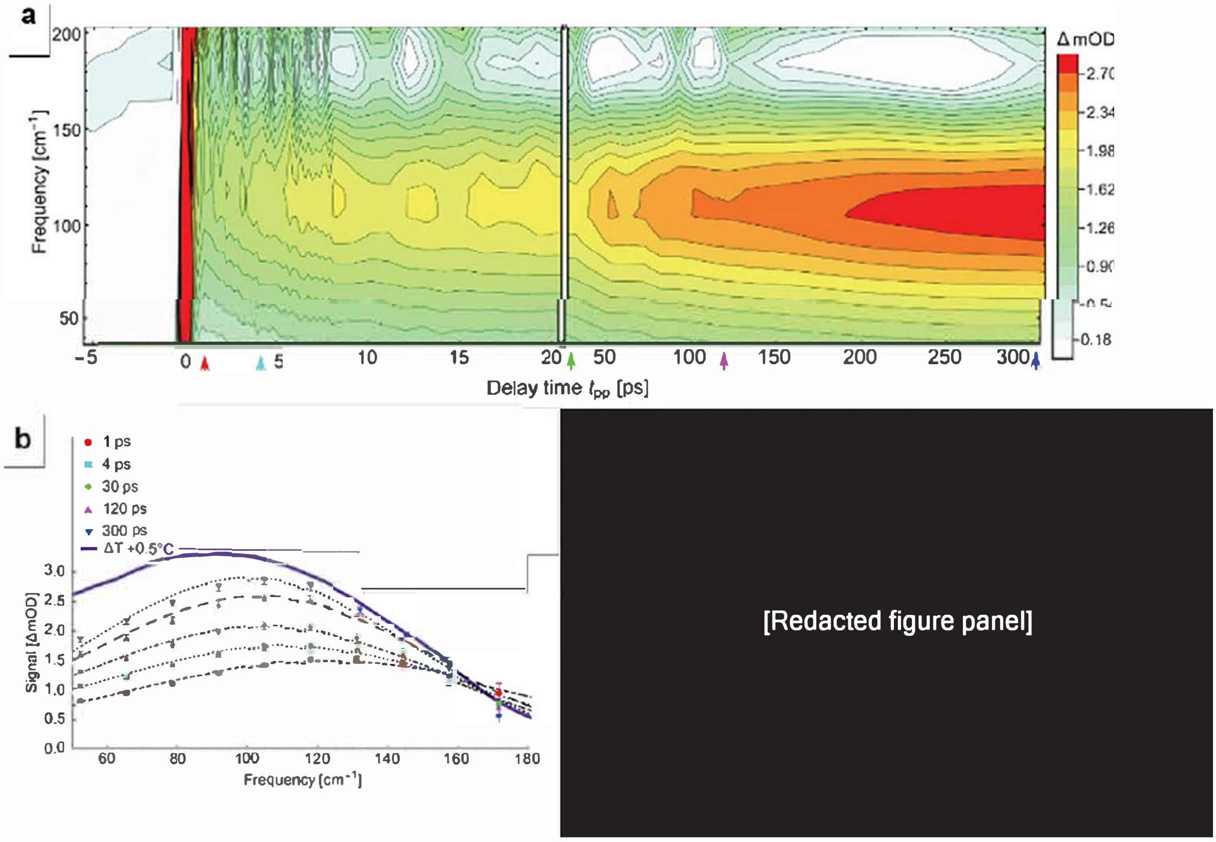

Figure R4: OPTP spectrum of dyes subsequent to photoexcitation. *a) 2D spectrum and selected 1D spectra (b) of 8-Hydroxypyrene-1,3,6-trisulfonic acid (HTPS) in water. The spectral feature at around 100* cm^{-1} *was attributed to propagating photoacoustic modes in the solvent. From Ockelmann et al. JPC B 2023*[7]*. c) Spectrum of* Ru(bpy) in water. *We compare the changes to the THz changes (black line, model based on experimental steady-state spectra and reported before*[2,3]*) expected when heating a sample of bulk water by 0.17 K (own unpublished data).*

We then performed single value decomposition (for all spectra and time points) and found two major components (Figure R5):

a) "Component 1" centred below 50 cm^{-1} with a maximum at around 30 cm^{-1} (= 1 THz) is dominant at short pump probe time delays (ps to several 10 of ps), remains persistent up to 200 ps but decreases with time.
b) "Component 2" > 100 cm^{-1} is present from the beginning but increases with time (Figure R6).

This spectral feature (component 1) is only present for (excited) SWCNTs and not observed for other fluorophores (Figure R4), that are expected to dissipate energy as heat into the solvent.

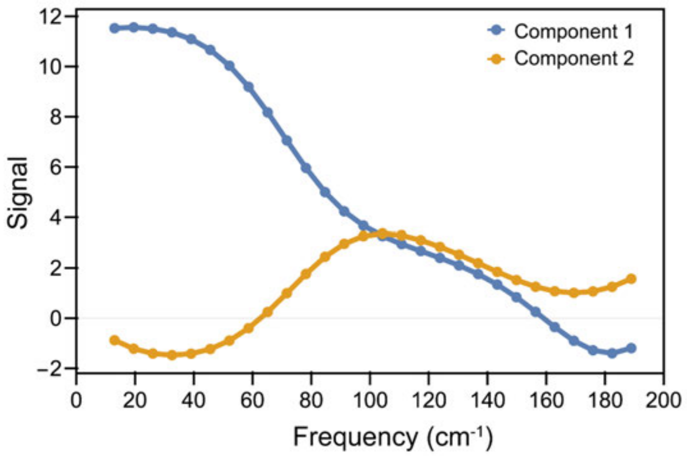

Figure R5/S27: Single value decomposition (SVD) of OPTP spectra of DOC-SWCNTs in water. *The first component (blue) has a maximum below 30 cm^{-1} (around 1 THz). The maximum of the second feature is centred at 100-120 cm^{-1}, resembling the spectrum of translational modes of the hydrogen bond network of water similar to heating of bulk water. SVD was performed (in Mathematica 14) with the spectra from 0.5 to 300 ps for a total of 31 time points from 13 cm^{-1} to 190 cm^{-1} (28 points each). The first two components represent 78% of the total weight of all components in the SVD.*

Thus, we interpret this spectral feature and response as interaction of the exciton with the water. The spectral response of component 1 is instantaneously present (< 0.5 ps) but cannot be distinguished in the period < 1 ps from the exciton relaxation to the bandgap (component 0) discussed in figure R2 but exists during the lifetime of the exciton (10 ps – 100 ps). Please note that any reorientation or motion of nuclei would require longer time delays. Typical time constants for any response in the water network requiring a reorientation of water molecules are on the order of 2-3 ps[2].

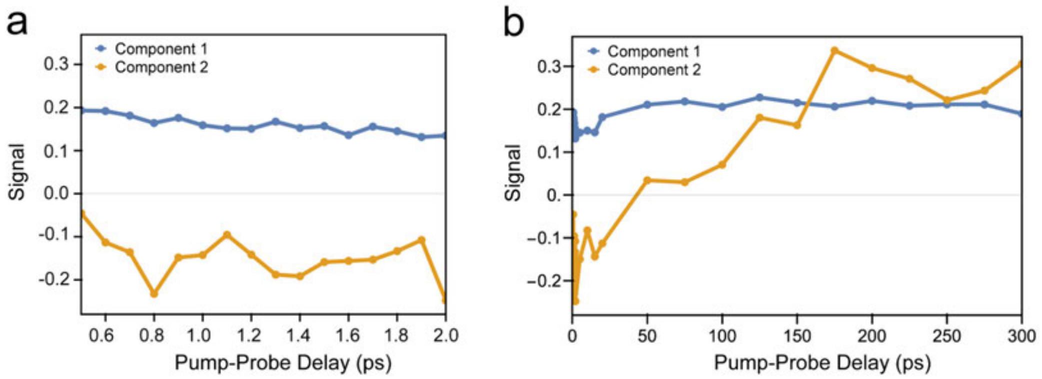

Figure R6/S28: Temporal and spectral contributions of the two THz features. *Partial contribution of component 1 (blue) and component 2 (orange) from SVD as a function of time delay between the optical pump and the THz probe.*

It is interesting to note that SWCNTs itself have a (static) THz spectrum that increases (Figure R7) around 50 cm^{-1}. It is in the range of component 1 in excited SWCNTs and overlaps with the Debye relaxation modes of water. However, this could be a coincidence and in the OPTP spectra (difference between excited and not excited samples) the static THz spectrum should be cancelled out. In contrast component 2 has a maximum around 100 - 120 cm^{-1} and resembles the spectrum expected in case of energy transfer from the solute to the solute, via phonons from SWCNTs to the water hydrogen bond network. This component increases over time (Figure R6) as expected for energy dissipation after an excitation pulse.

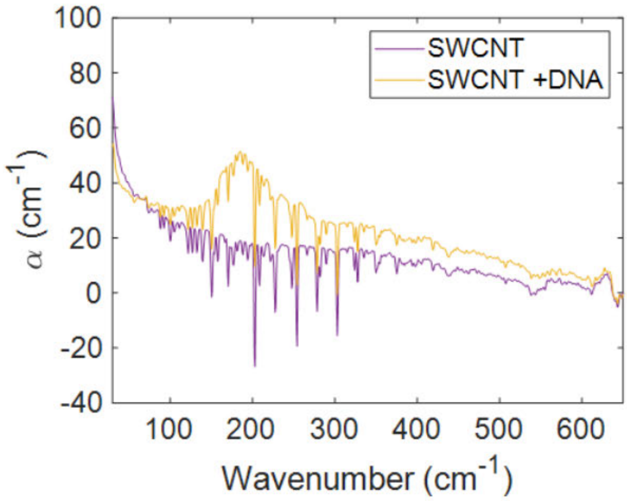

Figure R7: THz spectra of dry SWCNTs with and without DNA modification. *Note that these spectra were collected without optical excitation (i.e. no excitons are present). Own work from*[8]*.*

Thus, we interpret the OPTP THz experiments in the following way:

We found three distinct processes (in time decay and frequency):

Component 0: THz response, which decays < 1 ps and thus much faster than the exciton lifetime. It is interpreted as energy decay after excitation to the bandgap level.
Component 1: < 50 cm^{-1}, which decreases over the time course of ps to a few 100 ps similar to the exciton lifetime. We interpret it as direct exciton-water coupling.
Component 2: >100 cm^{-1}, which increases with time and resembles the effect of heating of water in an OPTP spectrum.

We understand that this is not a direct proof of quantum friction and coupling via phonons will be possible as well. However, we want to point out that the spectral response and the time evolution of component 1 has not been observed so far in other systems by heat transfer into water. It also fits the exciton decay lifetimes and thus we propose that this THz feature represents direct coupling of excitonic charge fluctuations in the SWCNT with water.

4.3) We additionally want to pinpoint to three important experimental results/parameters that support the observed effects and its interpretation as friction caused by excitons. They have so far been less discussed, but we believe they are an important part of our line of evidence:

a) **Chemical manipulation of the diffusion constant** means that a change of the fluorescence quantum yield of the SWCNT has a strong impact on its diffusion. SWCNTs like other NIR fluorophores have very low quantum yields of typically < 1% (e.g. 0,5 % for DNA-SWCNTs (see e.g. Spreinat et al JPC 2021[9]). Most absorbed light energy will dissipate in the end as heat (>99 %). If one takes into account the Stokes shift it would be even higher. Only a tiny fraction (< 1 %) will be emitted as light. Adding analytes can decrease/increase fluorescence (around 50 % for the maximum concentration, Figure 2a) and increase/decrease the diffusion constant by >100 %. This finding implies that even though the dissipated heat is changing very little i.e. < 1 %, the change of the diffusion constant is exceeding this change by two orders of magnitude. It pinpoints to a mechanism in which direct frictional forces between the excitons and the surrounding water exist, which are distinct from the overall heat transfer that stays basically the same in all the experiments in Figure 2 (see illustration in Figure R8). Of course the effect could be highly non-linear but the power-dependence (Figure 1) indicates a linear (exciton concentration) dependence.

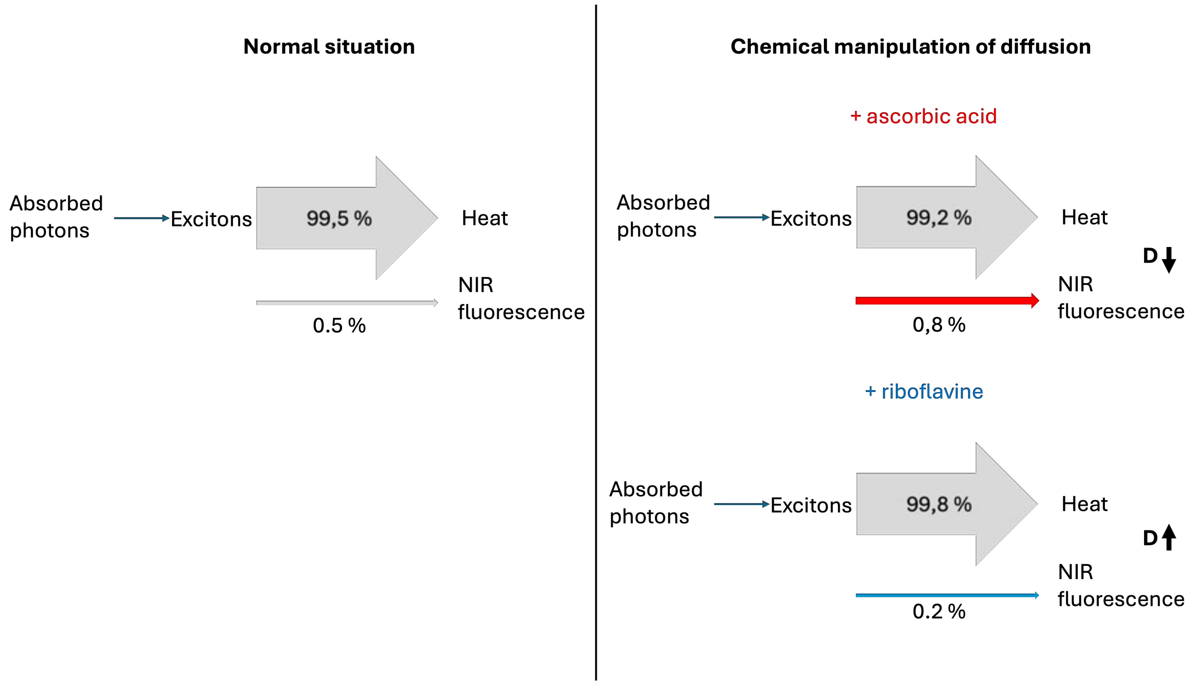

Figure R8: Schematic to illustrate that chemical manipulation affects mainly the effective exciton concentration but not heat dissipation. *The main argument is that because of the low quantum yield of SWCNT fluorescence (0,5 % for DNA/SWCNTs in water) nearly all energy is dissipated into the liquid as heat. Adding analytes changes the quantum yield and thus the lifetime*[5] *of the excitons (= concentration), which leads to a strong difference in diffusion D. The approximated changes in quantum yield (to around 0,8 % and 0,2 %) are based on the changes in the fluorescence spectra in Figure 2a.*

b) If only total heat transfer were the reason for the observed effects one would not expect an impact of **chemical shielding** (Figure 3 b). The impact of light on the diffusion constant should be the same for all organic corona phases (DNA, SDBS, SC, DOC) but it is clearly not (Figure 3a, Figure S18). Moreover, the reduction of the diffusion constant correlates qualitatively with the expected screening effect of the exciton by the water (red-shift of emission spectra). These results further support that the effect it is not only related to total energy transfer.

c) In Figure S12 we showed that for **D_2O-based buffer** the diffusion constant does decrease much less, which is in agreement with the mentioned experiment in reference 7. It is not a proof for quantum friction but supports an exciton induced quantum friction pathway.

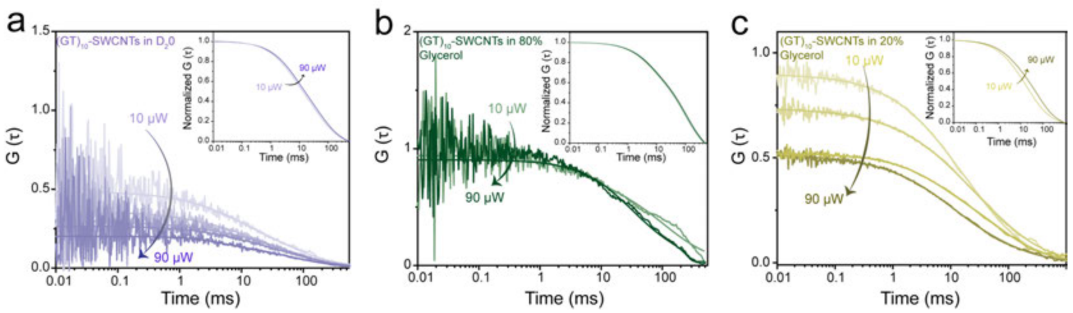

Figure R9/S12: Solvent effects on quantum friction. *Excitation power-dependent change (10 µW – 90 µW) of fluorescence autocorrelation functions of SWCNTs in different solvents. a) $(GT)_{10}$-SWCNTs in D_2O-based PBS (Table ST2), b) $(GT)_{10}$-SWCNTs in 80% glycerol in water (Table ST3), b) $(GT)_{10}$-SWCNTs in 20 % glycerol in water (Table ST4).*

We have now changed the text about the THz part as follows:

"The observed OPTP spectrum can be dissected into three distinct responses. An initial, instantaneous signal below 60 cm^{-1} (Figure 3c) increased linearly in amplitude with increasing optical pump power (Figure S25d) and decayed within < 1 ps (Figure S26). We interpret it as exciton relaxation to the bandgap energy level and energy dissipation to water. The decay times were faster than reported for dry gelatine/SWCNT films[1], which is similar to the faster cooling of hot electrons in graphene via quantum friction in water[10]. This process should not involve reorientation of the nuclei in the water network, which would take a longer response time. The OPTP spectrum (on time scales > 1 ps) can be further dissected into two additional responses (Figure 3d): A decreasing signal below 50 cm^{-1} and a subsequent increasing signal > 100 cm^{-1}, which becomes dominant for pump probe delays above 200 ps (Figure S29).

For a quantitative description, we carried out Single Value Decomposition (SVD) of the time dependent spectra (Figure S27, S28). The first component had a maximum around 30 cm^{-1} (1 THz) and persisted during the lifetime of the excitons (10 ps up to around 100 ps[4–6]). Such a feature had not been observed in THz OPTP spectra of other dyes after excitation[7]. The second component (100-120 cm^{-1}) continuously increased with increasing pump-probe delay time. It resembles collective translational modes (or acoustic phonon modes in the hydrogen bond network of water[11]) and OPTP changes expected for heating of water (Figure S29). Thus, we interpret the 30 cm^{-1} feature as direct coupling between the exciton and water, which appears instantaneously and decreases with decreasing exciton concentration. It is followed by a heat signature (>100 cm^{-1}). Therefore, we propose that the 30 cm^{-1} feature provides the pathway to exchange momentum via quantum friction. Near field radiative heat transfer (NFRHT) is another possible mechanism for the exciton to exchange energy with water but would rather contribute to the second (thermal) component (>100 cm^{-1}). This mechanistic picture aligns best with the experimental results, which showed that the change in the diffusion constant is proportional to the exciton concentration (Figuer 1c and Figure 2d)."

5) "Further confusion is generated by the implied equivalence between diffusion and friction. In its original formulation, quantum friction refers to a contribution to the friction force experienced by a liquid when flowing along a solid surface or through a nanotube. Here, the authors measure the diffusion coefficient of nanotubes in water, which is in principle linked to the water-nanotube friction coefficient through the Stokes-Einstein relation, but the link is far from obvious. This link should be made explicit in order to attribute the observed changes in diffusion to quantum friction."

Response: For Brownian motion the diffusion coefficient D is inversely proportional to the friction coefficient ζ via the Stokes-Einstein relation ($D=k_BT/\zeta$). Details on the inverse proportionality coefficient depend on the geometry, e.g. specific object length etc. In particular, for cylinders of length L and diameter d a theoretical relation between D and ζ has been derived and also applied to SWCNTs.[12] Therefore, from this formalism it follows directly that the increase of the friction coefficient is connected to a slowdown of SWCNT diffusion. However, we agree that the situation is distinct from water-flow over a solid because both water molecules and SWCNTs are moving due to Brownian motion. It makes understanding the impact of quantum friction on the SWCNT diffusion more difficult, but the MD simulation results translate these movements into friction coefficients and consequently diffusion properties via the Green-Kubo formalism (Figure S34). If the reviewer refers to understanding how the friction coefficient quantitatively depends on the light excitation, we believe that a quantitative theory of (optical) excited state-based quantum friction would be required.

Our experimental system is also more complex than in the MD simulation because the coating layer (corona) shows an additional effect (Figure 3b). However, due to the large aspect ratio of SWCNTs we expect that the cylinder approximation should be still valid so that the inverse relation between diffusion and friction coefficient holds[13]. In the manuscript, we calculate the friction coefficient from force correlation functions extracted from the MD simulations, and we discuss how it changes when a polarizable exciton is present in the SWCNT. We find that the exciton causes an increase of the friction coefficient with respect to the case where the exciton is not present. (Figure R10/ S34 in answer 6). In the simulations, we also calculated the diffusion coefficient for the SWCNT from the MSD plot (Figure 4c) and diffusion slowed down in the presence of a polarizable exciton. On the other hand, no slowdown of the diffusion was observed when the exciton is described by fixed charges (Figure 4d).

In the revised version we checked and streamlined the wording: We

a) clearly distinguish experiments that provide information only about diffusion

b) and suggest a mechanistic model in which we link the experiments (=diffusion measurements) with the interpretation and the role of friction via THz spectroscopy and MD modelling.

We hope that this way it becomes clear which information can be obtained from which method.

6) “The are more issues in the analysis of the MD simulations.
i) On the technical side: the authors use charges of 0.2e for the model electron and hole because the simulation is unstable for higher charges, but this information is buried in the methods section. This is an important piece of information that should be in the main text.”

Response: The simulations may become unstable for very high charges, which we had already mentioned in the main text:

“This initial choice was motivated to introduce a moderate perturbation and to maintain the stability of the simulation system and avoid crashing by over-polarization. Exact ±1 e would correspond to exactly one exciton present always throughout the experiment. However, smaller values are more likely because of lower exciton density (due to the average of excited and nonexcited time periods in pulsed as well as CW excitation schemes and the longer SWCNT length in experiments).”

Additionally, we added new controls to Figure R10/S34. It shows that the friction coefficient changes for charges of +1/ -1 e compared to +0.22/-0.22 e slightly increases but the difference in magnitude between non polarizable and polarizable remains.

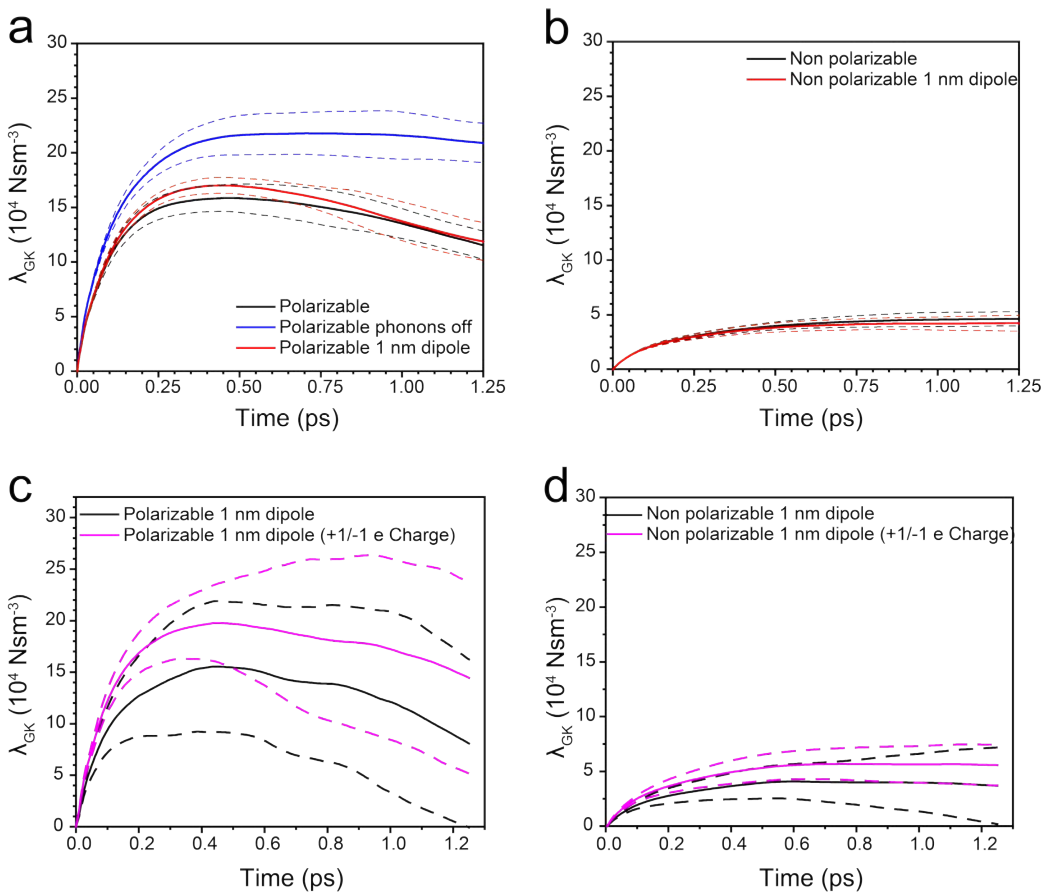

Figure R10/ S34: Comparison of Green-Kubo friction coefficient for polarizable and non-polarizable SWCNTs and different exciton charges. *a) Friction coefficient for polarizable SWCNTs without excitation, with excitation (1 nm dipole) and with excitation (1 nm dipole, fixed carbon atoms = switched off phonons). b) Friction coefficient for non polarizable SWCNTs with (1 nm dipole) and without excitation. The average friction coefficient is depicted with solid lines, and the uncertainty region, based on the standard deviation calculated from >n=1600 replicas, is denoted by dashed lines. In (a), (b) the excitons had a charge of ± 0.22 e. c) Friction coeffecient of polarizable and d) non polarizable SWCNTs with an exciton of ± 0.22 e (black) or ± 1.0 e (magenta) charge. Dotted lines show the uncertainty regime based on the standard deviation (n=500 trajectories). The simulated friction coefficient in water is around four times higher for the polarizable (a) compared to the nonpolarizable model (b). The friction coefficient increases with increasing charge either in the polarizable (c) or nonpolarizable (d) model. Eliminating lattice vibrations in the polarizable model increases the friction coefficient (a), which is in line with the increase in the cross-correlation shown in Figure S33 d and e.*

7) "ii) The authors imply that the correlation between the CNT dipole moment and the overall dipole moment of the water in the simulation box is somehow related to water-CNT momentum transfer. It is quite unclear to me why this would be the case. In fact, the polarizable force field

allows the CNT dipole moment to almost instantaneously adapt to the water configuration - it is then obvious that the water and CNT dipole moments should be more correlated than in the non-polarizable case, without any implications for CNT diffusion."

Response: The dipole-dipole correlation is only one part of the analysis and was performed to find out if there is spectral overlap. It is indeed related to the question whether energy transfer is possible in the system. We also compare in the manuscript the friction coefficient of the polarizable model (in which charge fluctuations are permitted) with the friction coefficient of the non-polarizable model (very little charge fluctuations). The results show that in the polarizable case, friction is much larger (Figure R10/S34 and question 6 above).

We have now directly compared the diffusion coefficient for a polarizable SWCNTs (with and without an exciton, original data from Figure 4b) with the diffusion coefficient for a non-polarizable SWCNT (with and without excitation). In the case of non polarizable SWCNTs, excitation (=addition of an exciton) did not change the diffusion coefficient (see Figure R12/4c,d below in response 9). It shows that – at least in this classical simulation –diffusion is only reduced by an exciton-like charge distribution if there is polarizability. Therefore, and also based on the quantum defect experiments we propose that charge fluctuations are required to observe the effect. We clarified this aspect in the discussion.

8) "iii) The authors state, "These results further confirm that an electron-hole (dipole) is not a sufficient condition to enhance the friction. The key element to couple charge dynamics to water dynamics is the dynamic nature of the exciton in SWCNTs (polarizability), resulting in enhanced friction". This statement seems to draw an equivalence between exciton dynamics due to diffusion and exciton polarizability. A key experimental result is that the light-induced diffusion modulation is suppressed by defects in the CNT, which are assumed to pin the excitons. The simulations might show slower CNT diffusion when the model exciton is polarizable, but they do not explain why a pinned exciton would have no effect on CNT diffusion."

Response: Indeed, the simulations show slower diffusion in the presence of polarizable excitons (Fig. R12/4c, see below). For non polarizable excitons, namely a simple static dipole, the slowdown is not observed (Figure 4d, see question 6 and 7 above and Figure R10/S34).

Exciton diffusion along the SWCNT axis has been experimentally determined over lengths of 100 nm[14] and is beyond the capability of our model. In our simulation of a short SWCNT (around 4 nm length due to computational costs) we can only reproduce exciton polarization (local charge fluctuations) and not exciton mobility/diffusion over longer distances. Therefore,

the question remains if local (around 1 nm = diameter of the SWCNT) charge fluctuations are causing the effect or rather the diffusing dipole moment as the reviewer points out. It is important to note that a “pinned” exciton doesn’t necessarily mean that it is not polarizable.

We now also performed experiments with another type of quantum defects (guanine quantum defects). They are less deep exciton traps and are expected to only slow down exciton diffusion/mobility[15] (Figure R11/S19). The power-dependence of diffusion decreases with increasing quantum defect density.

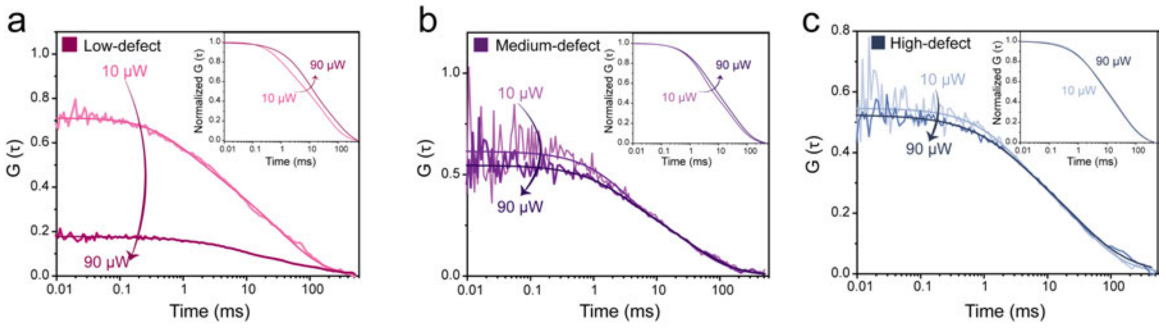

Figure R11/ S19: FCS experiments of $(GT)_{10}$-SWCNTs with less deep (guanine) quantum defects. *Comparison between a) low (reaction with 5 µM Rose Bengal), b) medium (10 µM Rose Bengal) and c) high (20 µM Rose Bengal) guanine defect density (samples prepared as described before[16]). The insets show the normalized and fitted autocorrelation functions.*

These results indicate that exciton diffusivity/mobility is more important than local polarizability. We added this aspect to the interpretation and discussion in the main manuscript.

9) “Results for the MSD with a non-polarizable force field are not shown, despite the question in my previous report.”

Response: We followed the suggestion of the reviewer (see last question below) and moved the particle tracking MSD data set (Figure 4d) to Figure 1. We added to Figure 4 the requested non polarizable control of the classical MD simulation. The results show that in the simulations polarizability is required to see a difference in diffusion between excited and non-excited states (Figure R12, 4c,d).

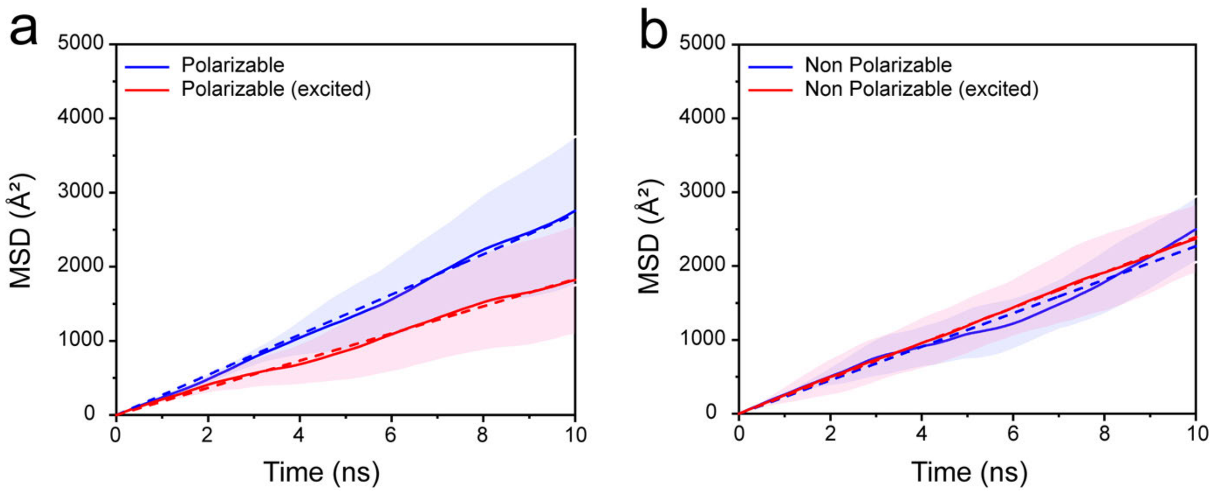

Figure R12/4c,d MD simulation of SWCNT diffusion for (a) polarizable and (b) nonpolarizable models and without excitation. *MD simulation of SWCNT diffusion for polarizable (c) and nonpolarizable (d) models and without excitation. The mean square displacement (MSD) is plotted versus time (solid lines). Dashed lines = linear fits. Shaded region = standard deviation (n=6 for excited and n=3 for non-excited). Here, the mean of 1 nm dipole and 2 nm dipole simulations is shown.*

10) "Could the role of defects be more complex than exciton pinning? Overall, I urge the authors to acknowledge uncertainties in interpretation rather than to sweep them under the rug."

Response: The experimental results show clearly that the diffusion constant of SWCNTs with defects are much less affected by the excitation intensity (Figure 3a). The same is observed for a different type of quantum defects (new Figure R11/S19, see question 8). We do not expect that the defects are affecting the surface chemistry because there are only a few quantum defects (at least for the defect type in figure 3a) per SWCNTs (see e.g. Ma et al. ACS Nano 2023[17] or Zaumseil et a. JPC 2022[18]), which corresponds to one changed carbon atom in >10 000 carbon atoms.

We think that the main difference is the very high mobility/diffusivity of free excitons (1-D) compared to trapped excitons (0-D), which is known from the literature and discussed in response 8 above.[19] What we currently do not understand is how dark versus bright excitons and thermal detrapping could affect the phenomenon (see e.g. He et al. Nature Materials 2018[20]). The distribution and location of quantum defects could also have an impact and even cause very interesting anisotropy effects on diffusion. We believe that these questions will motivate follow-up work by groups in the SWCNT/photonics field. We discuss these topics now in the revised version to pinpoint better to open questions in the future.

11) “iv) The analysis of the phonon effect in the framework of the MD simulation makes no sense to me as it is based, again, on the water-CNT dipole correlation.”

Response: The idea of this analysis is to show that our simulation model includes a direct coupling between the exciton (as classically modelled here) and water, and not only an indirect coupling mediated by the phonons. When we constrain the carbon positions, we eliminate the SWCNT lattice vibrations (phonons), which could mediate the interaction between electrons modes (here exciton fluctuations) and water collective modes. In this case, we show that the coupling between water and the SWCNT exciton dipoles is even more evident (Figure 4b). We agree with the reviewer that the analysis of the dipole-dipole cross correlation is addressing the direct energy exchange between the SWCNT and water and is not a proof for momentum transfer. It only shows that there is - under the limitations of the simulation - an interaction path beyond phonons but we cannot say anything about its importance in the experiment. We clarified this aspect in the revised version and added the simulation of the phonon effect (directly) on the friction coefficient (see question 7 and figure R10).

12) “v) Given all the above, "the MD simulations validated the experimental findings" is a gross overstatement. I urge the authors to be more careful in distinguishing fact from guesswork.”

Response: We rephrased this sentence to make clear that the MD simulations support the findings and provide an intuitive classical physics picture but are not a quantum mechanical proof. The simulations presented here aim to provide a simple classical model for the phenomenon, in the spirit of the model introduced by Bui et al. (Nano Letters 2023[21]). Similar to the Drude particles in this model, the exciton is modelled by us classically simulating a dynamical electron-hole charge distribution, which mimics electron-hole fluctuations. Our simulations show that charge fluctuations by an exciton couple to water and increase the water-SWCNT friction, as well as slow down diffusion of SWCNTs. What we discuss here is not a modified or new theory of quantum friction but serves as a classical model for the (quantum) friction effects caused by excitons.

The rephrased sentence is: “Therefore, these simplistic MD simulations support the experimental findings and pinpoint to the parameters that slow down SWCNT diffusion upon excitation. ”

13) “Finally, I think that the overall presentation and clarity are not on the level that can be expected from a Nature paper. The introduction still feels “meandering” – in the words of Reviewer 2 – despite the revision. The figures could be much better organized. For instance, the single CNT tracking is an important result that could be in Figure 1, and not in Figure 4 which is mainly about simulations. Some of the data in the supplementary figures would be welcome in the main figures (e.g., the single CNT tracking trajectories; note the label issue in Fig. S29). The main figures could be a lot more information-dense to avoid a constant back-and-forth between main paper and SI. The conclusion makes some statements with no logical sense, e.g. “These findings show that exciton-mediated quantum friction directly affects the motion of a nanoscale object and that there is momentum transfer”. What are the entities involved in the momentum transfer? The conclusion feels overall very weak and misses the fundamental implications of the findings for nanofluidic transport.“

Response: We thank the reviewer for the constructive comments. We made the following changes in the manuscript and SI (highlighted in yellow):

- We followed the suggestion and moved the wide-field particle tracking data into Figure 1. Thus, there is a clear structure with Figure 1 (diffusion measurements with varying power), Figure 2 (diffusion measurements under chemical manipulation), Figure 3 (Role of chemical shielding and THz spectroscopy), and Figure 4 (Classical simulations to support the proposed mechanism).
- Given the large number of supplementary figures, we did not want to make the main manuscript longer/less concise, and did not add further SI figures to the main manuscript.
- Figure S15 (previous Figure S29) is raw data and part of the analysis shown in the MSD plots of Figure 1d, and thus, we thought it might be redundant to show it twice. We checked Figure S15 (previous Figure S29) but could not identify an issue with the legend or label but we added an additional description to the caption.
- Overall, as outlined above in response 5, we made an effort to streamline and clarify the flow of arguments from diffusion measurements to a proposed mechanism via light induced quantum friction using THz spectroscopy and classical simulations.
- We extended the conclusion part to point to open questions as well as implications for nanofluidics and other fields.

References

(1) Xu, X.; Chuang, K.; Nicholas, R. J.; Johnston, M. B.; Herz, L. M. Terahertz Excitonic Response of Isolated Single-Walled Carbon Nanotubes. *J. Phys. Chem. C* **2009**, *113* (42), 18106–18109.

(2) Hoberg, C.; Talbot, J. J.; Shee, J.; Ockelmann, T.; Mahanta, D. Das; Novelli, F.; Head-gordon, M. Chemical Science Solvent Response That Promotes Excited-State Proton Transfer in Pyranine †. *Chem. Sci.* **2023**, *14*, 4048–4058. https://doi.org/10.1039/d2sc07126f.

(3) Lunkenheimer, P.; Emmert, S.; Gulich, R.; Köhler, M.; Wolf, M.; Schwab, M.; Loidl, A. Electromagnetic-Radiation Absorption by Water. *Phys. Rev. E - Stat. Nonlinear, Soft Matter Phys.* **2017**, *96* (December), 062607. https://doi.org/10.1103/PhysRevE.96.062607.

(4) Hertel, T.; Himmelein, S.; Ackermann, T.; Stich, D.; Ḱ, J. C. Diffusion Limited Photoluminescence Quantum Yields in 1-D Semiconductors : *ACS Nano* **2010**, *4* (12), 7161–7168.

(5) Sistemich, L.; Galonska, P.; Stegemann, J.; Ackermann, J.; Kruss, S. Near-Infrared Fluorescence Lifetime Imaging of Biomolecules with Carbon Nanotubes**. *Angew. Chemie - Int. Ed.* **2023**, *62* (24). https://doi.org/10.1002/anie.202300682.

(6) Jones, M.; Engtrakul, C.; Metzger, W. K.; Ellingson, R. J.; Nozik, A. J.; Heben, M. J.; Rumbles, G. Analysis of Photoluminescence from Solubilized Single-Walled Carbon Nanotubes. *Phys. Rev. B* **2005**, *71*, 115426. https://doi.org/10.1103/PhysRevB.71.115426.

(7) Ockelmann, T.; Hoberg, C.; Buchmann, A.; Novelli, F.; Havenith, M. Energy Dissipation into the Solvent during Proton Transfer Occurs via Acoustic Phonons. *J. Phys. Chem. B* **2023**, *127* (44), 9560–9565.

(8) Nalige, S. S.; Galonska, P.; Kelich, P.; Sistemich, L.; Herrmann, C.; Vukovic, L.; Kruss, S.; Havenith, M. Fluorescence Changes in Carbon Nanotube Sensors Correlate with THz Absorption of Hydration. *Nat. Commun.* **2024**, *15* (1), 6770. https://doi.org/10.1038/s41467-024-50968-9.

(9) Spreinat, A.; Dohmen, M. M.; Lüttgens, J.; Herrmann, N.; Klepzig, L. F.; Nißler, R.; Weber, S.; Mann, F. A.; Lauth, J.; Kruss, S. Quantum Defects in Fluorescent Carbon Nanotubes for Sensing and Mechanistic Studies. *J. Phys. Chem. C* **2021**, *125* (33), 18341–18351. https://doi.org/10.1021/acs.jpcc.1c05432.

(10) Yu, X.; Principi, A.; Tielrooij, K. J.; Bonn, M.; Kavokine, N. Electron Cooling in Graphene Enhanced by Plasmon–Hydron Resonance. *Nat. Nanotechnol.* **2023**, *18* (8), 898–904. https://doi.org/10.1038/s41565-023-01421-3.

(11) Heyden, M.; Sun, J.; Funkner, S.; Mathias, G.; Forbert, H.; Havenith, M.; Marx, D. Dissecting the THz Spectrum of Liquid Water from First Principles via Correlations in Time and Space. *Proc. Natl. Acad. Sci. U. S. A.* **2010**, *107* (27), 12068–12073.

(12) Tirado, M. M.; Martínez, C. L.; de la Torre, J. G. Comparison of Theories for the Translational and Rotational Diffusion Coefficients of Rod-like Macromolecules. Application to Short DNA Fragments. *J. Chem. Phys.* **1984**, *81* (4), 2047–2052.

(13) Lee, A.; Cognet, L. Length Measurement of Single-Walled Carbon Nanotubes from Translational Diffusion and Intensity Fluctuations. *J. Appl. Phys.* **2020**, *128* (22), 224301. https://doi.org/10.1063/5.0031194.

(14) Lüer, L.; Hoseinkhani, S.; Polli, D.; Crochet, J.; Hertel, T.; Lanzani, G. Size and Mobility of Excitons in (6, 5) Carbon Nanotubes. *Nat. Phys.* **2009**, *5* (1), 54–58.

(15) Zheng, Y.; Weight, B. M.; Jones, A. C.; Chandrasekaran, V.; Gifford, B. J.; Tretiak, S.; Doorn, S. K.; Htoon, H. Photoluminescence Dynamics Defined by Exciton Trapping Potential of Coupled Defect States in DNA-Functionalized Carbon Nanotubes. *ACS Nano* **2021**, *15* (1), 923–933. https://doi.org/10.1021/acsnano.0c07544.

(16) Galonska, P.; Mohr, J. M.; Schrage, C. A.; Schnitzler, L. Guanine Quantum Defects in Carbon Nanotubes for Biosensing. **2023**, 1–26. https://doi.org/10.1021/acs.jpclett.3c00358.

(17) Ma, C.; Schrage, C. A.; Gretz, J.; Akhtar, A.; Sistemich, L.; Schnitzler, L.; Li, H.; Tschulik, K.; Flavel, B. S.; Kruss, S. Stochastic Formation of Quantum Defects in Carbon Nanotubes. *ACS Nano* **2023**, *17* (16), 15989–15998. https://doi.org/10.1021/acsnano.3c04314.

(18) Sebastian, F. L.; Zorn, N. F.; Settele, S.; Lindenthal, S.; Berger, F. J.; Bendel, C.; Li, H.; Flavel, B. S.; Zaumseil, J. Absolute Quantification of Sp 3 Defects in Semiconducting Single-Wall Carbon Nanotubes by Raman Spectroscopy. *J. Phys. Chem. Lett.* **2022**, *13* (16), 3542–3548. https://doi.org/10.1021/acs.jpclett.2c00758.

(19) Miyauchi, Y.; Iwamura, M.; Mouri, S.; Kawazoe, T.; Ohtsu, M.; Matsuda, K. Brightening of Excitons in Carbon Nanotubes on Dimensionality Modification. *Nat. Photonics* **2013**, *7* (9), 715–719. https://doi.org/10.1038/nphoton.2013.179.

(20) He, X.; Htoon, H.; Doorn, S. K.; Pernice, W. H. P.; Pyatkov, F.; Krupke, R.; Jeantet, A.; Chassagneux, Y.; Voisin, C. Carbon Nanotubes as Emerging Quantum-Light Sources. *Nat. Mater.* **2019**, *18* (7), 770–770. https://doi.org/10.1038/s41563-019-0406-4.

(21) Bui, A. T.; Thiemann, F. L.; Michaelides, A.; Cox, S. J. Classical Quantum Friction at Water–Carbon Interfaces. *Nano Lett.* **2023**, *23* (2), 580–587. https://doi.org/10.1021/acs.nanolett.2c04187.

Referee 1

Questions by the reviewer are shown in blue. We highlighted the changes in response to the reviewer comments in yellow in the main manuscript.

1) „The authors have carried out substantial revision work, including additional experiments and data analysis, largely addressing the points raised in my previous report. In particular, I am happy to see that the revised manuscript is more careful in drawing conclusions, ensuring that these are better supported by the data. Below are the few points that I still find somewhat unclear or inaccurate. „

Response: We thank the reviewer very much for the time to review our manuscript and the constructive input that improved it.

2) „In the Introduction "Optical pump Terahertz (THz) probe experiments showed very short time scales of cooling via quantum friction in water, which was attributed to the coupling of graphene surface plasmon modes and water charge fluctuations in the frequency range of the librational mode of water in the THz region" – there is still a confusion between energy transfer and quantum friction. In Ref. 7, the very fast electron cooling could be attributed to direct energy transfer (near-field radiative heat transfer) between the graphene electrons and water, which is not quantum friction. Indeed, by design, OPTP can only probe energy transfer, and not momentum transfer. But because OPTP probes energy transfer between the degrees of freedom that are potentially involved in quantum friction, it can provide indirect indication of the latter."

Response: Yes, we agree that OPTP only reports about changes in absorption (i.e. energy changes). We checked the text to make sure that this interpretation is consistent everywhere. Of note, indirectly one could interpret the features in our THz spectra > 100 cm^{-1} (based on the literature) as acoustic phonon modes linked to momentum changes but we agree that the direct interpretation of THz changes is only related to energy absorption.

We modified the mentioned sentence:

"Optical pump Terahertz (THz) probe experiments showed faster cooling in water compared to other solvents, which was attributed to Near Field Radiative Heat Transfer (NFRHT) between graphene surface plasmon modes and water charge fluctuations in the frequency range of the librational mode of water in the THz region.[7]"

3) „The discussion of the OPTP experiments has become significantly clearer and I now agree that they qualitatively support the light-induced quantum friction mechanism put forward by the authors. But there is still a conceptual confusion, because quantum friction and NFRHT are presented as two distinct mechanisms that correspond to two different features in OPTP spectra: "Therefore, we propose that the 30 cm-1 feature provides the pathway to exchange momentum via quantum friction. Near field radiative heat transfer (NFRHT) is another possible mechanism for the exciton to exchange energy with water but would rather contribute to the second (thermal) component (>100 cm-1)". However, I insist that the only thing that OPTP measurements can reveal is NFRHT between electrons and water. I think that the reported measurements are likely indicating exactly that, and this comes in qualitative support of the importance of quantum friction in the CNT diffusion. Indeed, as discussed in Ref. 7, the usual ("classical") pathway of electron-liquid energy transfer goes through phonons, and I expect that the energy transfer from thermalized phonons to water should indeed look like water heating on 10-100 ps timescales. The faster dynamics in the low-frequency part of the spectrum could thus indeed correspond to direct energy transfer (NFRHT) between the excitons and water. NFRHT is not "just heating": it can occur between non-thermalized degrees of freedom. I strongly suggest rephrasing the statement quoted above."

Response: We interpret the reviewer's point the following way: There are electronic fluctuations of the solid (SWCNT) and the liquid (water) that can couple to each other. In the OPTP spectra we find evidence for energy transfer via NFRHT (for the short time scales). It is a hint that momentum exchange of SWCNTs with water could take place via the same coupling pathway. We believe that the reviewer wants to clarify that quantum friction and NFRHT are not mutually exclusive processes. They can share the same pathway (coupling of electronic fluctuations) but transfer different properties (momentum versus energy) and depend on different parameters (velocity differences or statistical differences in occupied states). Thus, we modified the respective sentence accordingly and hope that it matches the explanation of the reviewer:

"Such a spectral response is expected for energy transfer from thermalized phonons to water via translational modes or propagating acoustic phonons[52]. Thus, we interpret the 30 cm^{-1} feature as direct coupling between the exciton and water, which appears instantaneously and decreases with decreasing exciton concentration. We propose that the 30 cm^{-1} feature reports NFRHT via a coupling that could also serve as a pathway to exchange momentum via quantum friction. It is followed by a heat signature (>100 cm^{-1})."

3) „I am still puzzled by the cross-correlation analysis of the exciton dipole and total water

dipole in the MD simulations. The response letter states “The dipole-dipole correlation is only one part of the analysis and was performed to find out if there is spectral overlap. It is indeed related to the question whether energy transfer is possible in the system”. I assume that the authors refer to spectral overlap between the water and the dipole’s charge fluctuations. If so, then I am still convinced that this cross-correlation analysis brings no additional information regarding the possibility of energy transfer or quantum friction between CNT excitons and water. Indeed, the polarizable force field used by the authors is such that the dummy charges adapt almost instantaneously to the water configuration: the dummy charges are much faster than water, so there is no overlap between the bare charge fluctuations spectra. This is different from the setup of Bui et al, where the Drude charges evolve on timescales that are similar to water timescales. Now, because the force field is polarizable, there will necessarily be a cross-correlation between the exciton dipole and the water dipole, and I still do not see how this cross-correlation is indicative of anything. What is indeed interesting with regard to the experiment is that a polarizable dipole added to the CNT has a much greater effect on diffusion than a frozen dipole. The paper is already dense and information-rich: I suggest dropping the cross-correlation analysis (and the discussion of phonons that comes with it) and only presenting the MSD analysis of the simulations – which is sufficient to make the point!“
Response: We agree that the cross-correlation analysis is not necessary for the claims of the manuscript. Given that it only caused confusion we removed it from the manuscript along with the discussion of the phonons.

4) “It very interesting that dipole moment fluctuations impact diffusion, while a static dipole does not – and I agree that this is qualitatively in line with the experimental observation that pinning the exciton suppresses the effect on diffusion. Thus, one may wonder whether the average dipole moment is at all important. Indeed, excitons in pristine CNTs should rather have no net dipole moment. I would suggest carrying out a simulation with a dipole-free polarizable exciton (e.g. three charged rings, +,-,+) to definitively confirm the role of fluctuations versus static charge distribution.”
Response: This is a very interesting suggestion. We performed a simulation to further check this aspect. In particular, we have compared the friction coefficients for the 2-ring model (normal exciton) and the 3-ring model (exciton with zero average dipole). As suspected by the reviewer the dipole moment fluctuations have a larger impact than the average value of the dipole itself (new Figure S7).
The friction coefficient for the 3-ring system was similar (or slightly higher) as the friction coefficient for the normal exciton (polarizable model) and both are much larger than the friction coefficient for a static dipole (non-polarizable model). We want to note that the small simulation

box (relative to exciton mobility) of around 4 nm cannot (fully) model the impact of exciton diffusion (around 100 nm expected diffusion length).

This additional simulation further confirms the importance of charge fluctuations versus static charge distributions. We want to note that this simulation approach should be expanded in the future to other modifications and geometries. For example, trions (2 electrons + one hole or vice versa) are also experimentally accessible, which could reveal exciting novel phenomena.

In summary, the static dipole moment itself seems to be less relevant for slowing down diffusion than the movement of the exciton/charges along the axis.

5) “Single value decomposition” – shouldn’t it be “singular value decomposition”?

Response: Yes, thank you for the hint. We changed it accordingly.
